# A specific fluorescent probe reveals compromised activity of methionine sulfoxide reductases in Parkinson's disease†
†Electronic supplementary information (ESI) available: The characterization of probes, experimental procedures, supporting data and original spectra (^1^H NMR, ^13^C NMR, and MS) of the final 23 compounds. See DOI: 10.1039/c6sc04708d



**DOI:** 10.1039/c6sc04708d

**Published:** 2017-01-27

**Authors:** Liangwei Zhang, Shoujiao Peng, Jinyu Sun, Juan Yao, Jie Kang, Yuesong Hu, Jianguo Fang

**Affiliations:** a State Key Laboratory of Applied Organic Chemistry , College of Chemistry and Chemical Engineering , Lanzhou University , Lanzhou , Gansu 730000 , China . Email: fangjg@lzu.edu.cn

## Abstract

Oxidation of methionine residues to methionine sulfoxide (MetSO) may cause changes in protein structure and function, and may eventually lead to cell damage. Methionine sulfoxide reductases (Msrs) are the only known enzymes that catalyze the reduction of MetSO back to methionine by taking reducing equivalents from the thioredoxin system, and thus protect cells from oxidative damage. Nonetheless, a lack of convenient assays for the enzymes hampers the exploration of their functions. We report the discovery of Msr-blue, the first turn-on fluorescent probe for Msr with a >100-fold fluorescence increment from screening a rationally-designed small library. Intensive studies demonstrated the specific reduction of Msr-blue by the enzymes. Msr-blue is ready to determine Msr activity in biological samples and live cells. Importantly, we disclosed a decline of Msr activity in a Parkinson's model, thus providing a mechanistic linkage between the loss of function of Msrs and the development of neurodegeneration. The strategy for the discovery of Msr-blue would also provide guidance for developing novel probes with longer excitation/emission wavelengths and specific probes for Msr isoforms.

## Abstract 2

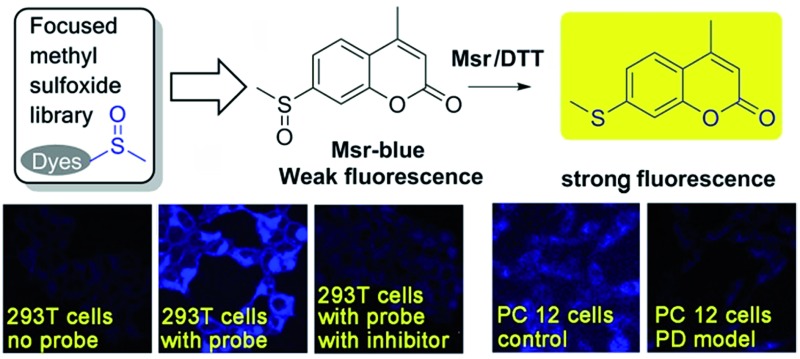
A general strategy for designing probes of methionine sulfoxide reductases was reported and a first turn on probe was disclosed.

## Introduction

Methionine side chains in proteins/peptides are among the most susceptible to oxidation by reactive oxygen/nitrogen species, leading to the formation of methionine sulfoxide (MetSO) residues. Methionine sulfoxide reductases (Msrs), the only characterized enzymes known to reduce the MetSO residues in oxidatively damaged proteins/peptides to methionine residues by taking reducing equivalents from the thioredoxin system, are virtually universal among all aerobic organisms.^1–5^ Msr A and Msr B are two major isoforms in mammals and specifically reduce the *S*-MetSO epimer and *R*-MetSO epimer, respectively.^6,7^ The structure of the Msr-substrate complex shows that the methyl sulfoxide moiety fits in the surface-exposed active site of the enzyme, where the oxygen of the sulfoxide forms an intensive hydrogen bonding network with several polar residues. However, the other parts of the substrate are flexible.^8–10^ Thus, Msrs have broad substrate specificity and can reduce a variety of methyl sulfoxide-containing molecules, ranging from protein bound MetSO residues to several small molecules, such as dimethyl sulfoxide, methionine sulfoxide, sulindac and sulforaphane.^11–13^ Despite the important roles of Msrs in regulating multiple cellular events,^1,4,14,15^ the lack of convenient tools to detect their activity hampers the exploration of the function of the enzymes under physiological or pathophysiological conditions.

**Scheme 1:**
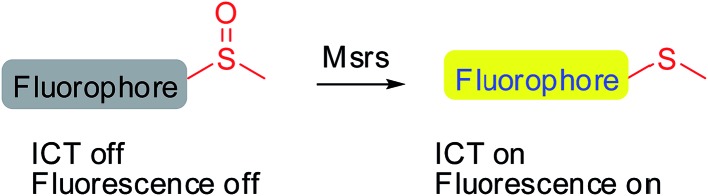
Design of potential probes for Msrs.

Developing specific probes for a particular biomolecule is of great importance to unveil its function in physiological/pathophysiological processes. Among various detection techniques, fluorescence-based assays are widely adopted due to their high sensitivity, operational simplicity, and super biocompatibility. Therefore, the past years have witnessed an increase in the number of studies that focus on developing diverse fluorescent probes for studying various protein activities^16–27^ as well as small biomolecule functions.^28–35^ One of the outstanding and well-recognized strategies in designing fluorescent probes is the manipulation of the intramolecular charge transfer (ICT) process, as the alteration of such a process is usually accompanied with a pronounced change in the absorbance/fluorescence profiles of a molecule, which forms the basis of the fluorescence sensing. Manipulation of the ICT process could be easily achieved by tuning the electronic character of the substituents attached to a fluorophore by exchanging electron donating groups with electron withdrawing groups or *vice versa*.^36,37^ The reaction that is catalyzed by Msr converts sulfoxides to the corresponding sulfides, which enables the transformation of an electron withdrawing group to an electron donating group. Taking into consideration the broad substrate scope of Msrs, we thus hypothesized that attachment of the methyl sulfoxide moiety to certain fluorophores might generate turn-on probes for the enzymes (Scheme 1).

**Fig. 1:**
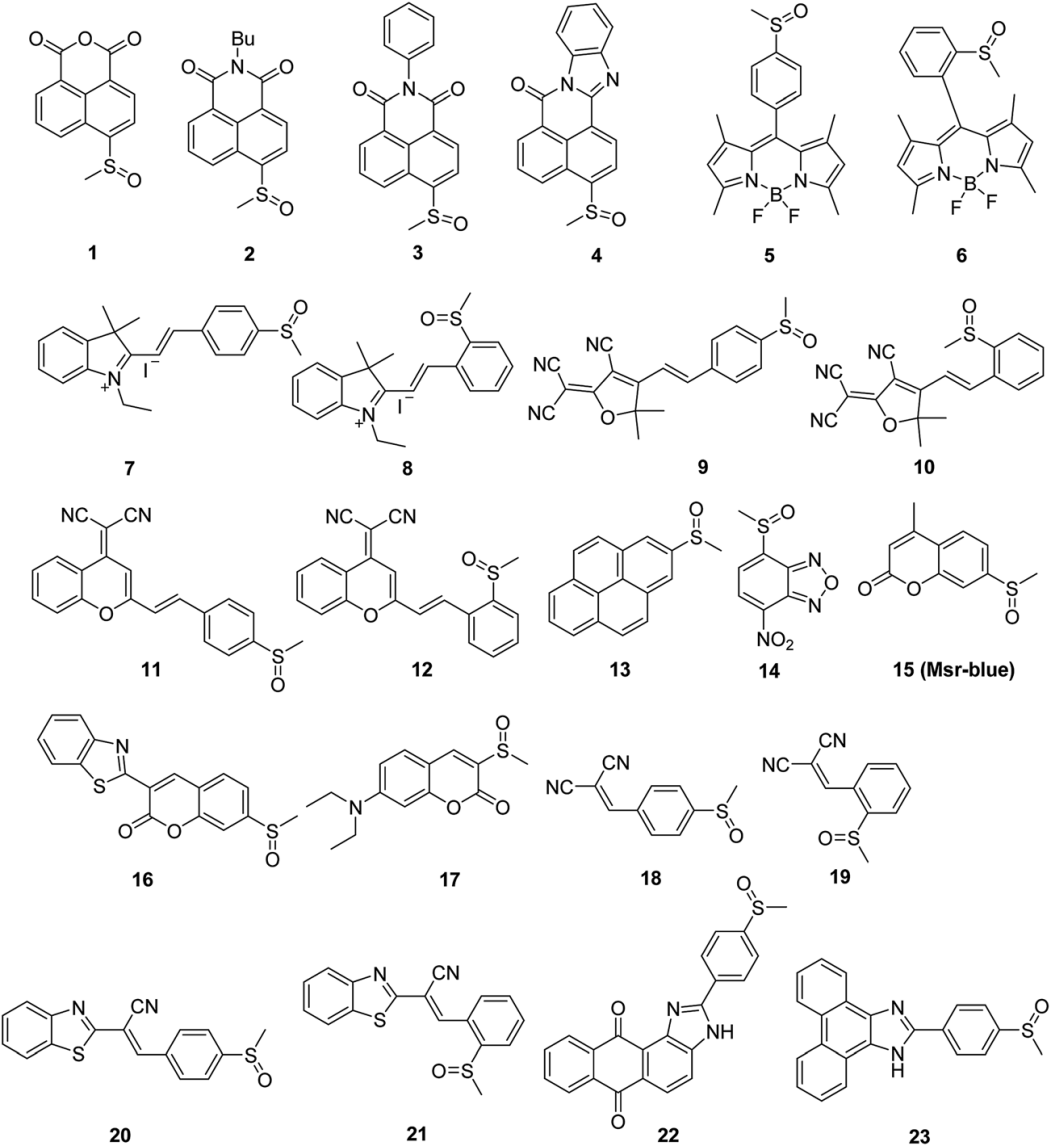
The sulfoxides library.

We created a focused small library containing 23 dyes uniformly decorated with a methyl sulfoxide group (**1–23**, Fig. 1), and evaluated their reduction by Msr. The synthetic routes are illustrated in the ESI† (Schemes S1–3). Characterizations and the original spectra (^1^H NMR, ^13^C NMR, and MS) of the final compounds are included in the ESI† (Fig. S4–71). Compound **15** (Msr-blue) was identified as the first turn-on fluorescent probe for Msr with a more than 100-fold fluorescence increment. The specific activation of Msr-blue by Msr was further demonstrated. Msr-blue is ready to image enzyme function in live cells and to measure enzyme activity in biological samples. With the aid of Msr-blue, a decline in enzyme activity in a cellular model of Parkinson's disease (PD) was disclosed for the first time. We expect the probe would facilitate an understanding of the physiological/pathophysiological functions of Msrs. In addition, the concept of the probe design would also advance the development of novel Msr probes with longer excitation/emission wavelengths as well as specific Msr A and Msr B probes.

## Results and discussion

### Probe library synthesis

Diverse fluorophores, such as naphthalimide, BODIPY, cyanine and coumarin, were selected to construct the target probe library. A uniform methyl sulfoxide group was introduced by oxidation of the corresponding precursor methyl sulfides under mild conditions to furnish the final 23 potential probes (Fig. 1). The straightforward synthetic routes are illustrated in the ESI† (Schemes S1–3). All of the final 23 compounds were fully characterized by MS and NMR, and their original spectra (^1^H NMR, ^13^C NMR, and MS) are included in the ESI† (Fig. S4–71). The purity of the probes **15** and **16** was determined to be 98% and 99%, respectively (Fig. S72†).

### Probe screening

After constructing a small library of methyl sulfoxide dyes, we next screened the library for potential Msr fluorogenic substrates (Table 1). As we employed DTT as the electron donor for the reduction of sulfoxides under Msr catalysis, we thus firstly investigated whether DTT causes interference in the assay. As shown in Table 1, fluorescence signals of five compounds (**1**, **2**, **3**, **14** and **17**) were efficiently activated by DTT (*F*/*F*_0_ > 10). This is not surprising as sulfoxides are prone to be reduced by thiols.^36,38^ In addition, compounds **10** and **22** and their corresponding sulfides (**10′** and **22′**) have very weak fluorescence in aqueous solutions. Consequently, these seven compounds were excluded in the next studies using both DTT and Msr A. To our delight, two (**15** and **16**) out of the sixteen DTT-negative compounds showed a 32.3- and 11.6-fold increase in their fluorescence signal, respectively. We therefore picked up compounds **15** and **16** for the follow-up validation studies.

**Table 1:** Screen of the probes^*a*^

Probes	*λ*_ex_/*λ*_em_ (nm)	DTT (*F*/*F*_0)_	DTT + Msr A (*F*/*F*_0_)
**1**	395/505	11.2	
**2**	390/500	14.5	
**3**	385/505	216	
**4**	425/570	1.7	2.0
**5**	508/528	1.2	1.3
**6**	508/520	1.7	1.7
**7**	440/570	0.97	2
**8**	430/640	1.0	1.0
**9**	450/625	0.22	1.51
**10**	—^*b*^	—^*b*^	—^*b*^
**11**	390/550	1.92	2.32
**12**	410/550	0.81	0.53
**13**	370/497	1.32	1.47
**14**	375/550	10.7	
**15** (Msr-blue)	335/438	1.08	32.33
**16**	420/490	2.0	11.6
**17**	390/505	24.9	
**18**	375/480	1.0	1.1
**19**	380/580	1.0	1.0
**20**	400/510	0.37	2.38
**21**	340/490	0.57	1.42
**22**	—^*b*^	—^*b*^	—^*b*^
**23**	330/450	2.1	1.87

^*a*^The assays were performed by incubating probes (10 μM) with DTT (5 mM) or DTT (5 mM)/Msr A (3 μg ml^–1^, 120 nM) for 1 h at 37 °C in TE buffer (50 mM Tris–HCl, 1 mM EDTA, pH 7.4).

^*b*^There is only very weak fluorescence in the aqueous solution. All compounds were dissolved in DMF to prepare stock solutions, and the organic solvent in the final assay mixture is no more than 0.1% (v/v). The folds of fluorescence changes were expressed as *F*/*F*_0_.

### Probe validation

**Fig. 2:**
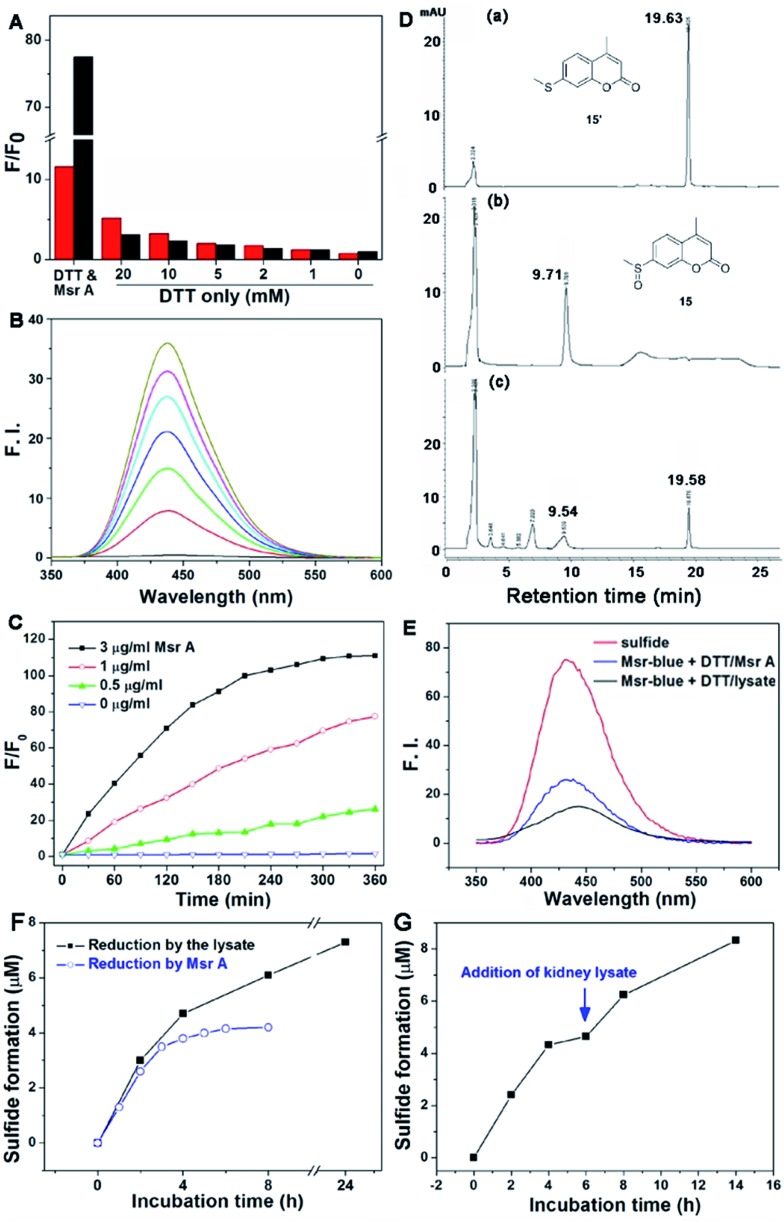
Reduction of Msr-blue by Msr. (A) Effects of DTT in the reduction of **16** and Msr-blue. The probes (10 μM) were incubated with DTT (20 mM) and Msr A (3 μg ml^–1^, 120 nM) or DTT only (0–20 mM) for 3 h, and the fold of the fluorescence increment (*F*/*F*_0_) was determined. The excitation/emission wavelengths for Msr-blue and **16** are 335/438 nm and 420/490 nm, respectively. (B) Time-dependent activation of Msr-blue by Msr A. Msr-blue (10 μM) was incubated with DTT (5 mM) and Msr A (3 μg ml^–1^, 120 nM). The emission spectra (*λ*_ex_ = 335 nm) were recorded every 30 minutes. (C) Time- and dose-dependent increase of the fluorescence of Msr-blue. The probe was incubated with DTT (5 mM) and Msr A (0–3 μg ml^–1^, 0–120 nM), and *F*/*F*_0_ was determined (*λ*_ex_ = 335 nm, *λ*_em_ = 438 nm). (D) Exclusive conversion of Msr-blue to the sulfide **15′**. The probe (10 μM) was incubated with DTT (5 mM) and Msr A (3 μg ml^–1^, 120 nM) for 6 h, and the mixture was analyzed by HPLC. (E) Fluorescence spectra of the pure sulfide **15′**, the reaction mixture from (D) and the reaction mixture of Msr-blue (10 μM) incubated with DTT (5 mM) and mouse kidney protein extract for 2 h (*λ*_ex_ = 335 nm). (F) Reduction of Msr-blue by Msr A and mouse kidney lysate. The probe was incubated with DTT (5 mM) in the presence of Msr A (3 μg ml^–1^, 120 nM) or mouse kidney lysate (29.2 mg protein per ml), and the formation of the sulfide **15′** was determined by HPLC. (G) Reduction of Msr-blue by a combination of Msr A and mouse kidney lysate. Msr-blue was incubated with DTT (5 mM) and Msr A (6 μg ml^–1^, 240 nM) for 6 h. Then, the mouse kidney lysate (29 mg protein per ml) was added and the incubation continued. The formation of the sulfide **15′** was determined by HPLC. All reactions were performed at 37 °C in TE buffer.

Firstly, we performed experiments to confirm the response of both probes to DTT by using varying concentrations of the reductant. As shown in Fig. 2A, high concentrations of DTT cause a significant elevation in the fluorescence signal of **16**, while DTT appears to affect **15** much less. In addition, compound **16** shows only about a maximum 10-fold increase in emission under Msr A catalysis (Fig. S1†). Considering the potential interference of DTT with **16** and the lower fluorescence increase of **16** under enzyme catalysis, we thus decided to focus on the probe **15** in the following experiments. The DTT concentration was fixed at 5 mM in the following assays, as DTT at this concentration causes negligible interference in the fluorescence signal. Since the probe **15** emits blue fluorescence after activation by Msr A, we termed it as Msr-blue. Msr-blue responded to Msr A in both a time- and dose-dependent manner (Fig. 2B and C), and more than a 100-fold increase in the emission was observed. HPLC analysis of the reaction demonstrated that Msr-blue was converted to its corresponding sulfide (**15′**) under catalysis by either the purified Msr A or a cell lysate (Fig. 2D and S2†). When incubation of Msr-blue (10 μM) with DTT and Msr A in TE buffer (50 mM Tris–HCl, 1 mM EDTA, pH 7.4) took place for 6 h at 37 °C, the desired sulfide **15′** was found in the reaction mixture with ∼40% conversion. At the time point of analysis, the remaining amount of Msr-blue (6.2 μM) plus the product **15′** (3.8 μM) was equal to the amount of the starting Msr-blue (10 μM), indicating that the probe was exclusively converted to the expected sulfide **15′**. It should be noted that Msr-blue is a racemic mixture containing both *S*-epimers and *R*-epimers, and Msr A should only convert the *S*-epimer to the sulfide **15′**. Incubation of Msr-blue with mouse kidney lysate gave similar results as those for the purified enzyme, yet with a lower conversion rate of the probe (∼14% conversion, Fig. S2†). The sulfide **15′** and the reaction mixtures which were catalyzed by purified Msr A and by the cell lysate gave similar fluorescence profiles (Fig. 2E), supporting the conversion of Msr-blue to the sulfide **15′** under Msr catalysis. Taken together, we firmly concluded herein that the probe Msr-blue could be reduced by Msr to the corresponding sulfide, leading to a more than 100-fold increase in the fluorescence signal.

To further demonstrate the specific reduction of one epimer in the racemic mixture of Msr-blue, we further compared the reduction of the probe by the pure Msr A enzyme and the mouse kidney lysate, which contains both Msr A and Msr B enzymes. As shown in Fig. 2F, the reaction catalyzed by Msr A could be finished within 6 h, and the maximal formation of the sulfide was less than 50% (∼42% after 8 h). In contrast, the kidney extract reduces more Msr-blue: the yield of the corresponding sulfide is >60% at 8 h, and could generate >70% of the sulfide after incubating the probe for 24 h. After the Msr A-catalyzed reaction was complete, we added the kidney extract to the reaction mixture. Again, formation of the sulfide continues (Fig. 2G), and ∼80% total sulfide was generated after incubating the mixture with the lysate for an additional 8 h. Our results support the conclusion that Msr A could specifically reduce one epimer (likely the *S*-isoform) of Msr-blue, and that preparation of enantiomers of Msr-blue would yield specific probes for Msr A/B enzymes.

### Specific activation of Msr-blue by Msrs

**Fig. 3:**
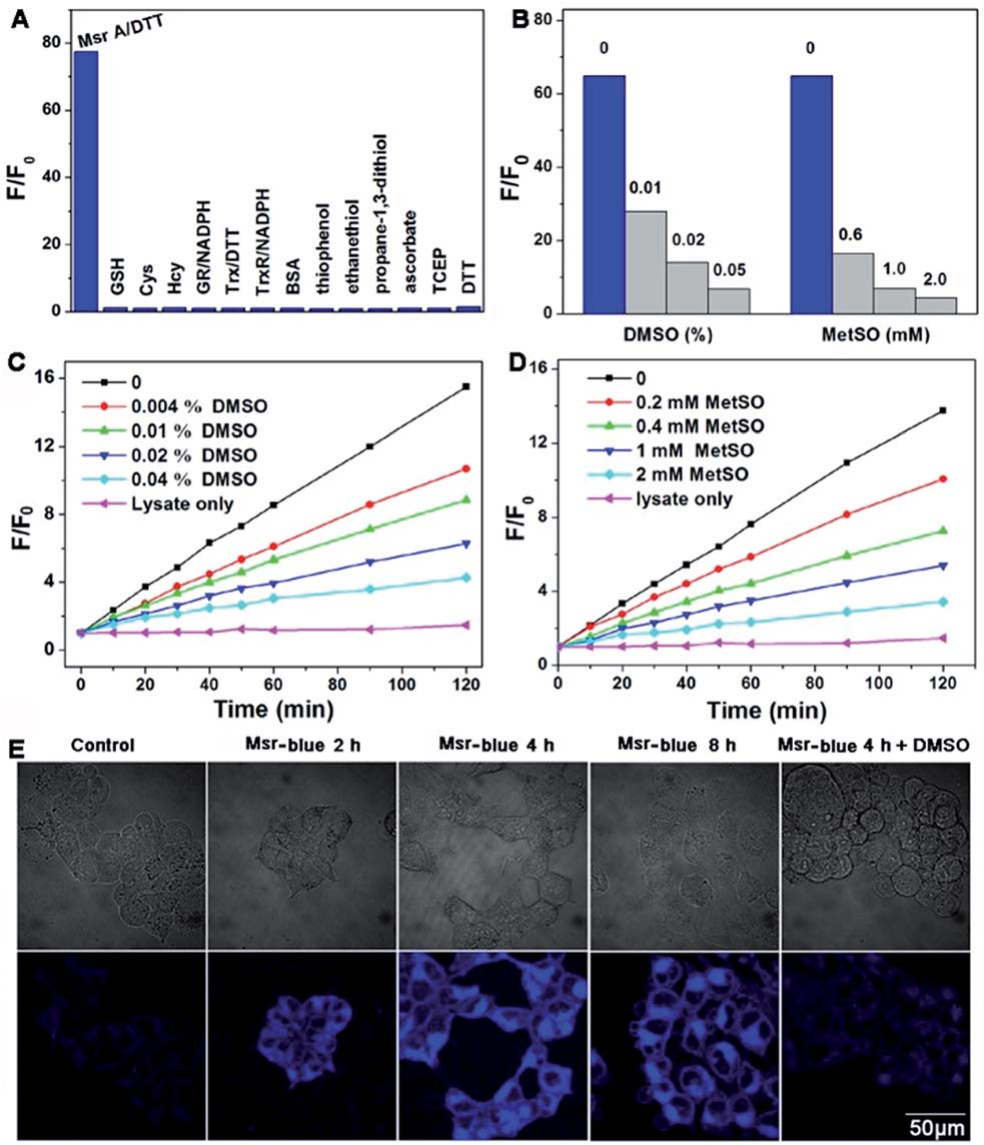
Selective reduction of Msr-blue by Msr. (A) Activation of the fluorescence signal by different analytes, including Msr A (3 μg ml^–1^, 120 nM) with DTT (5 mM) and small molecules (1 mM) or proteins: 20 μg ml^–1^ (380 nM) for GR, 1 mg ml^–1^ (15 μM) for BSA, 0.2 mM for NADPH, 80 μg ml^–1^ (6.7 μM) for Trx and 11 μg ml^–1^ (200 nM) for TrxR. After addition of the probe (10 μM) and incubation for 3 h, *F*/*F*_0_ was determined (*λ*_ex_ = 335 nm, *λ*_em_ = 438 nm. (B) Inhibition of the pure Msr A-mediated reduction of Msr-blue by DMSO and MetSO. The probe (10 μM) was incubated with DTT (5 mM) and Msr A (1.8 μg ml^–1^, 72 nM)) with or without DMSO and MetSO for 2 h, and *F*/*F*_0_ was determined (*λ*_ex_ = 335 nm). Inhibition of the mouse kidney lysate-mediated reduction of Msr-blue by DMSO (C) and MetSO (D). The probe (10 μM) was incubated with DTT (5 mM) and mouse kidney protein extract, and *F*/*F*_0_ was determined (*λ*_ex_ = 335 nm). All reactions were performed at 37 °C in TE buffer. (E) Imaging Msr activity in live cells. 293T cells were incubated with Msr-blue (10 μM). To inhibit Msr activity, the cells were first treated with DMSO (0.2%) followed by incubation with Msr-blue for 4 h. The cells were washed with PBS and photographed under a fluorescent microscope.

**Fig. 4:**
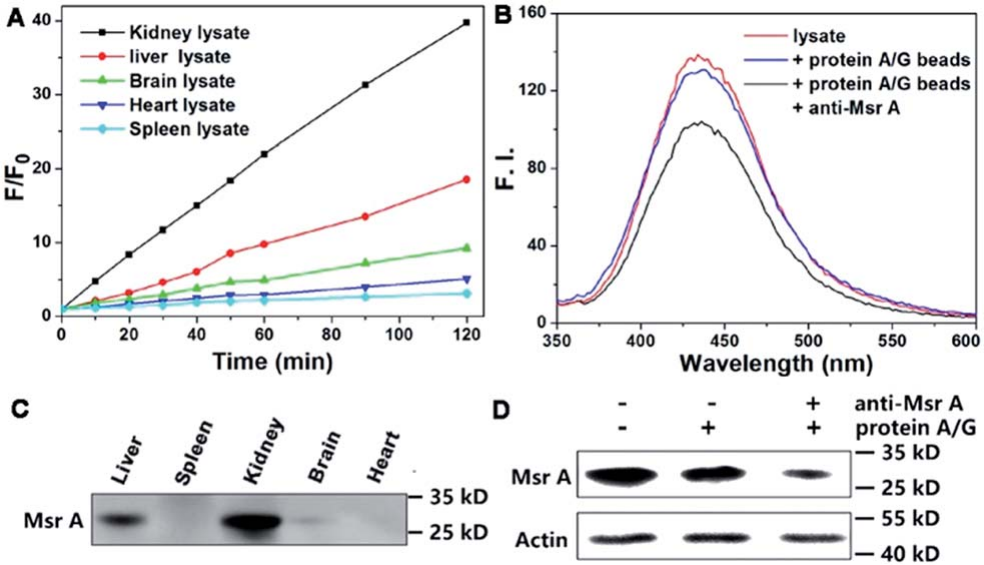
Specific reduction of Msr-blue by Msr. (A) Determination of Msr activity in mouse organs. (B) Reduction of Msr-blue by mouse kidney lysate before and after IP of Msr A. (C) Determination of Msr A protein content in mouse organs by Western blots. The IP efficiency was confirmed by Western blots (D).

Next, we performed experiments to answer whether the fluorescence signal of Msr-blue could be specifically turned on by Msrs. Firstly, the response of Msr-blue to a panel of potential reducing agents and enzymes was tested (Fig. 3A). Under our experimental conditions, Msr A turns on the fluorescence with nearly 80-fold increase, while none of the tested species, such as various small molecule thiols, glutathione reductase (GR), thioredoxin (Trx), thioredoxin reductase (TrxR), bovine serum albumin (BSA), ascorbate, and tris(2-carboxyethyl)phosphine (TCEP), turn on the fluorescence. We then measured the reduction of Msr-blue in the presence of the Msr inhibitors dimethyl sulfoxide (DMSO) and MetSO. As shown in Fig. 3B–D, both inhibitors suppressed the catalytic reduction of Msr-blue by either the purified enzyme or the protein extract from mouse kidneys. We further examined the reduction of the probe by live cells. As shown in Fig. 3E, the live 293T cells have a weak background signal. After incubation with Msr-blue, the blue fluorescence signal increases time-dependently, and it appears to reach saturation after incubation for 4 h. Again, the fluorescence signal could be inhibited by pretreatment of the cells with DMSO. Imaging Msr activity in HL-60 cells and its inhibition by DMSO was also demonstrated (Fig. S3†). Next, we applied the probe to measure the Msr activity in different mouse organs, *i.e.* liver, spleen, kidney, brain and heart. As shown in Fig. 4A, the kidney appears to show the highest activity, followed by the liver, and then the brain. The heart and spleen show the lowest activity. Coincidentally, the protein level of Msr A follows the same trend: kidney > liver ≫ brain > heart ∼ spleen, which matches well with the results from the Msr-blue-based fluorescence assay (Fig. 4C). To further demonstrate the specific reduction of Msr-blue by Msr, we compared the reduction of the probe by mouse kidney lysates before and after an immunodepletion of Msr A. The depletion of Msr A from the lysate remarkably decreased the fluorescence intensity (∼25% decline, Fig. 4B). The efficiency of the immunoprecipitation (IP) was validated by Western blots (Fig. 4D). It was estimated by densitometry using ImageJ that more than 60% of Msr A was removed from the lysate. The Msr-blue reduction activity in the lysate after IP is likely from both Msr B and the remaining Msr A, since the Msr A antibody does not crossreact with Msr B. Taken together, we concluded herein that Msr-blue is a specific fluorogenic substrate of Msrs. The kinetics of the Msr A-catalyzed Msr-blue reduction indicated that Msr-blue is a good substrate for Msr A with a *K*_m_ of 120 μM and a *k*_cat_ of 0.4 s^–1^ (Fig. S73†), which are similar to the values of other MetSO-containing substrates.^15,39^

### Decline of Msr activity in a PD model

**Fig. 5:**
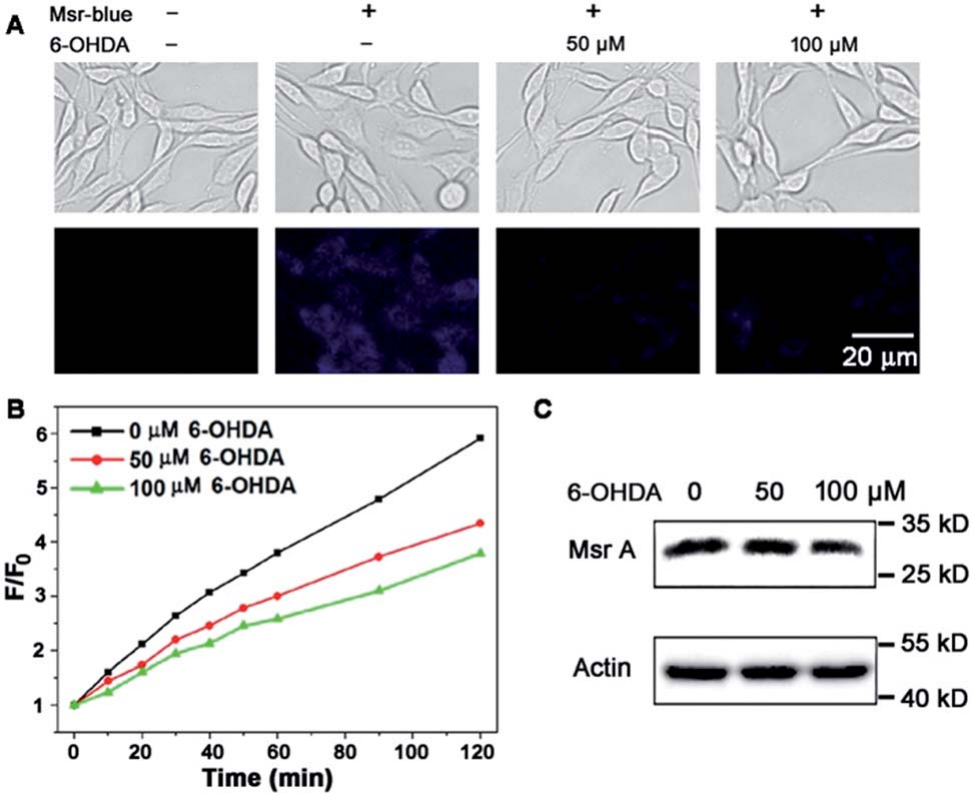
Decline of Msr activity in a PD model. (A) Imaging Msr activity in live PC 12 cells treated with or without 6-OHDA. (B) Determination of Msr activity in PC 12 cell lysates. PC 12 cells were treated as described in (A), and the time-dependent reduction of Msr-blue by the lysates was determined (*λ*_ex_ = 335 nm). (C) Determination of Msr A protein level in the lysates prepared in (B) by Western blots.

Due to the critical roles of Msrs in defending against oxidative stress and preventing accumulation of misfolded proteins, a compromised function of Msr has been linked to the development of aging-associated disorders like neurodegenerative diseases.^4,40–42^ In line with this observation, an overexpression of Msr A confers benefits against the development of PD,^40,41,43^ while knockout of the enzyme enhances the progression of neurodegeneration.^42^ Markesbery *et al.* reported the decrease of Msr A activity in brains that have been affected by Alzheimer's disease.^44^ However the activity of Msrs in PD tissues or models is unknown. 6-Hydroxydopamine (6-OHDA) is a well-established neurotoxin used to generate PD models in animals or cells.^45,46^ We employed the 6-OHDA-treated PC 12 cells as a cellular model of PD^47–49^ and applied Msr-blue to probe the function of Msrs in the cells. As illustrated in Fig. 5A, 6-OHDA treatment causes a remarkable decline in the enzyme activity, revealed by the decrease in the fluorescence intensity of the cells. As the activity of Msrs is supported by the thioredoxin system in cells, we thus further determined their activity in the lysate by supplying DTT as a reductant to exclude the possible alteration of function by the thioredoxin system. Consistent with the Msr-blue imaging results, the activity of Msr decreased by ∼30% in the drug-treated cells compared to the control groups (Fig. 5B). Analysis of the protein levels of Msr A indicated no significant alteration of the protein expression after 50 μM of 6-OHDA treatment, yet a slight decrease after 100 μM of 6-OHDA treatment (Fig. 5C). Taken together, with the aid of Msr-blue, a decline of the Msr activity in a PD model was disclosed for the first time.

The methionine side chain is highly susceptible to oxidation particularly under conditions of oxidative stress. Oxidation of methionine residues to MetSO, usually leading to a change of protein structure and alteration of protein function, could be reversed by taking reducing equivalents from the thioredoxin system catalyzed by Msr. *Via* catalyzing regeneration of methionine from MetSO, Msr regulates the function of a diverse variety of proteins, ranging from protein kinases to transcription factors and metabolic enzymes.^1,4,15^ However, the lack of convenient methods to assay the enzyme activity hampers the exploration of their functions. During preparation of the manuscript, we noticed that Dr Makukhin *et al.* reported the first ratiometric fluorescent probe, Sulfox-1, for Msr A.^50^ In this paper, the authors used a chiral starting material to construct the optical pure probe (*S*)-Sulfox-1, which guided us to prepare (*S*)- or (*R*)-epimers of the probe for selective Msr A and Msr B imaging. However, compared to Msr-blue, there are some weak points of Sulfox-1. Firstly, the authors demonstrated that Msr A could reduce the probe, giving a ∼7-fold decrease in emission at 542 nm and a very slight increase (less than 1.5-fold) in emission at 576 nm. In contrast, Msr-blue is almost non-fluorescent, and displays a >100-fold increase in fluorescence upon responding to Msr A. This off–on character and large magnitude elevation of the fluorescence signal provide the easiest way to measure the enzyme activity. Secondly, the application of Sulfox-1 is quite limited: the authors only performed live cell imaging using bacterial cells. The practical application of Msr-blue was demonstrated by various experiments. This probe is suitable to image Msr function in different types of mammalian cells and assay the enzyme activity in biological samples. More importantly, Msr-blue was used to probe the activity of Msr in a PD model, which reveals a remarkable decline in the enzyme activity in this disease for the first time. Thirdly (and most importantly), the specificity of Sulfox-1 for the enzyme is not well addressed as the paper lacks such data. It's well known that sulfoxides are prone to be reduced by thiols. Indeed, the authors found that DTT could reduce Sulfox-1. We performed intensive research to demonstrate that Msr-blue is activated by Msr specifically, which guarantees its application to explore the physiological/pathological function of Msrs. The disadvantage of Msr-blue is its short excitation/emission wavelengths. Based on the strategy described in this paper, we are currently preparing novel probes by conjugation of the methyl sulfoxide group to more fluorophores with green or red emission, and expect to discover specific probes with longer excitation/emission wavelengths.

## Conclusions

In summary, we have rationally designed a focused sulfoxide library, and identified Msr-blue as the first specific off–on probe of Msr with a >100-fold increase in the emission. The probe is ready to use and multiple practical applications of the probe have been demonstrated. With the aid of Msr-blue, we disclosed the compromised Msr activity in a PD model, highlighting the relevance of Msr dysfunction to the progress of neurodegeneration. We expect that Msr-blue would be a powerful tool to facilitate dissecting the functions of Msrs. In addition, the strategy for the discovery of Msr-blue also sheds light on the development of Msr probes with longer excitation/emission wavelengths as well as specific Msr A or Msr B probes.
