# Supplementary material for: A specific fluorescent probe reveals compromised activity of methionine sulfoxide reductases in Parkinson's disease
Source: Chem Sci. 2017 Jan 27;8(4):2966–72. doi: 10.1039/c6sc04708d (PMC5382841; doi:10.1039/c6sc04708d)
Supplement: Supplementary file 1 [file SC-008-C6SC04708D-s001.pdf]

Electronic Supplementary Material (ESI) for Chemical Science.
This journal is © The Royal Society of Chemistry 2017

Supporting Information

A specific fluorescent probe reveals compromised activity of methionine sulfoxide reductases in Parkinson's disease

Liangwei Zhang, Shoujiao Peng, Jinyu Sun, Juan Yao, Jie kang, Yuesong Hu, and Jianguo Fang*

State Key Laboratory of Applied Organic Chemistry and College of Chemistry and Chemical Engineering, Lanzhou University, Lanzhou, Gansu 730000, China

Scheme S1. Synthesis of compounds **1-6**.

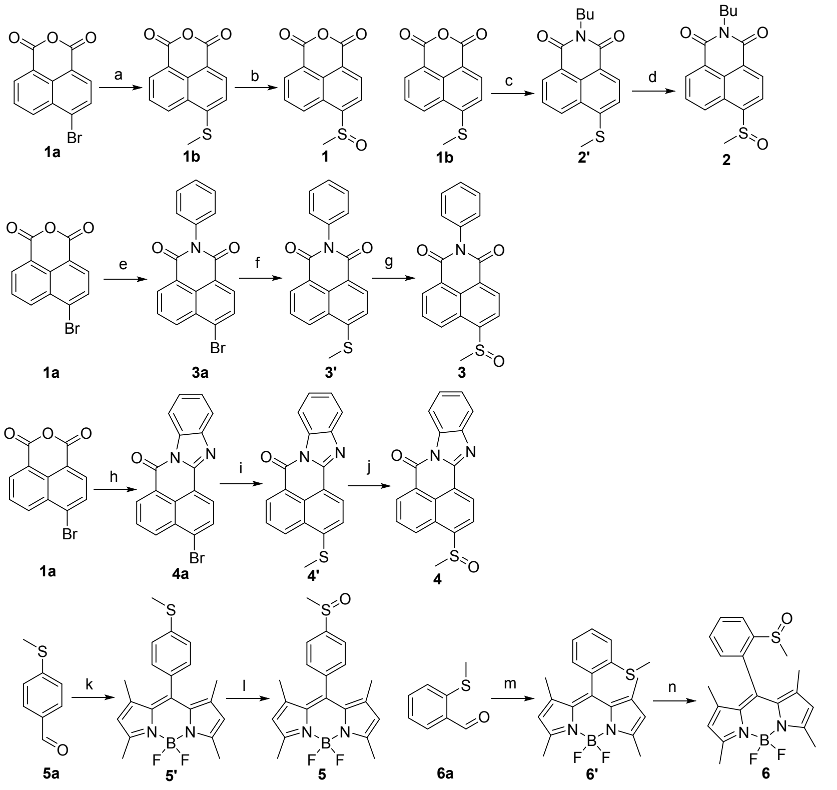

Reagents and conditions: (a) DMF/CH_3SNa, rt, 92%; (b) *m*-CPBA/DCM, rt, 73%;(c) $C_4H_9NH_2$/EtOH, reflux, 62%; (d) NaClO/THF, rt, 89%; (e) $C_6H_5NH_2$/AcOH, reflux, 51%; (f) DMF/CH_3SNa, rt, 89%; (g) *m*-CPBA/DCM, rt, 67%; (h) *o*-Phenylenediamine/AcOH, reflux, 56%; (i) DMF/CH_3SNa, rt,86%; (j) *m*-CPBA/DCM, rt, 78%; (k)TFA/DCM, DDQ, NEt_3/BF_3•Et_2O, 23%; (l) *m*-CPBA/DCM, rt, 70%; (m) TFA/DCM, DDQ, NEt_3/BF_3•Et_2O, 17%; (n) *m*-CPBA/DCM, rt, 63%.

Scheme S2. Synthesis of compounds **7-15**.

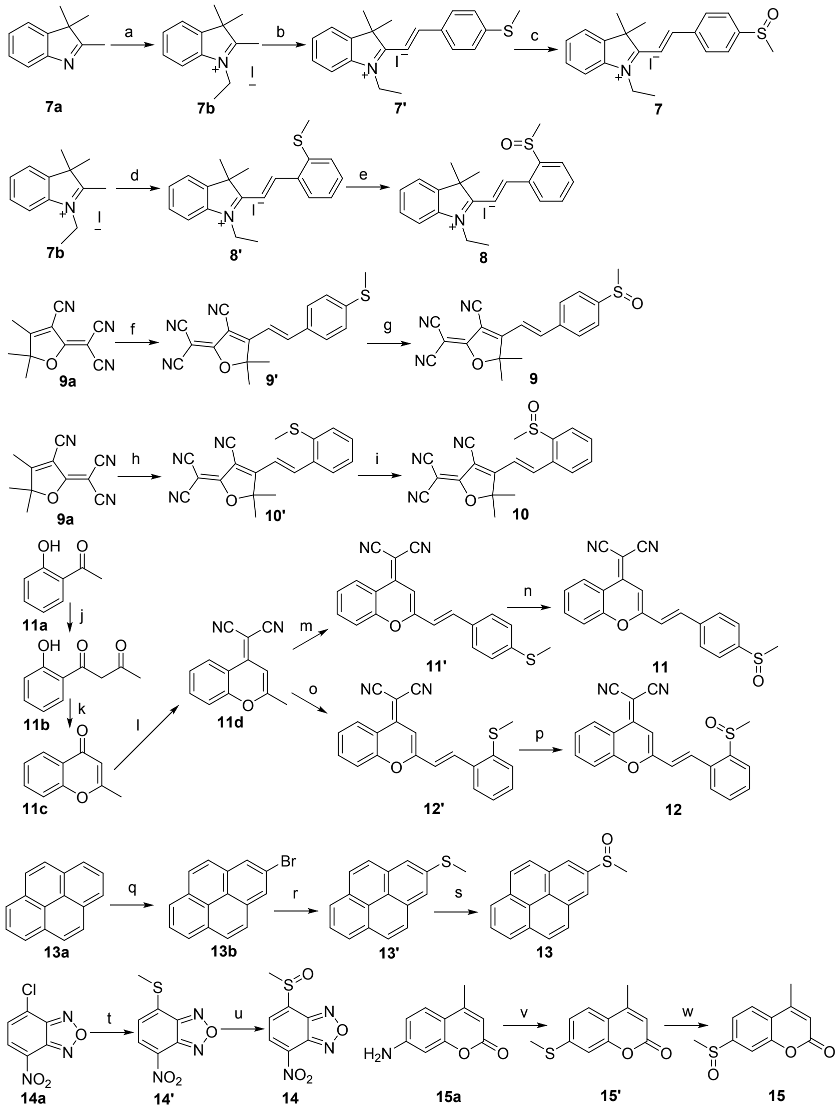

Reagents and conditions: (a) Toluene/EtI, rt, 87%; (b) Piperidine/EtOH, reflux, 69%;(c) *m*-CPBA/DCM, rt, 78%; (d) Piperidine/EtOH, reflux, 56%; (e) *m*-CPBA/DCM, rt, 76%; (f) Piperidine/EtOH, reflux, 43%; (g) *m*-CPBA/DCM, rt, 67%; (h) Piperidine/EtOH, reflux, 51%; (i) *m*-CPBA/DCM, rt, 72%; (j) Na/EtOH/$CH_3COOCH_2CH_3$, rt, 50%; (k) H_2SO_4/CH_3COOH, 67%; (l) Malononitrile/acetic anhydride, reflux, 30%; (m) Piperidine/HOAc/toluene, reflux, 41%; (n) *m*-CPBA/DCM, rt, 63%;(o) Piperidine/ HOAc/toluene, reflux, 39%; (p) *m*-CPBA/DCM, rt, 63%. (q) NBS/DCM, rt, 73%; (r) DMF/CH_3SNa, rt, 89%; (s) *m*-CPBA/DCM, rt, 78%; (t) Tris-HCl buffer (pH=8.0)/CH_3SNa, rt, 63%; (u) *m*-CPBA/DCM, rt, 68%; (v) $NaNO_2$/HCl/THF-H_2O, CH_3SNa, reflux, 21%; (w) *m*-CPBA/DCM, -78℃, 86%.

Scheme S3. Synthesis of compounds **16-23**.

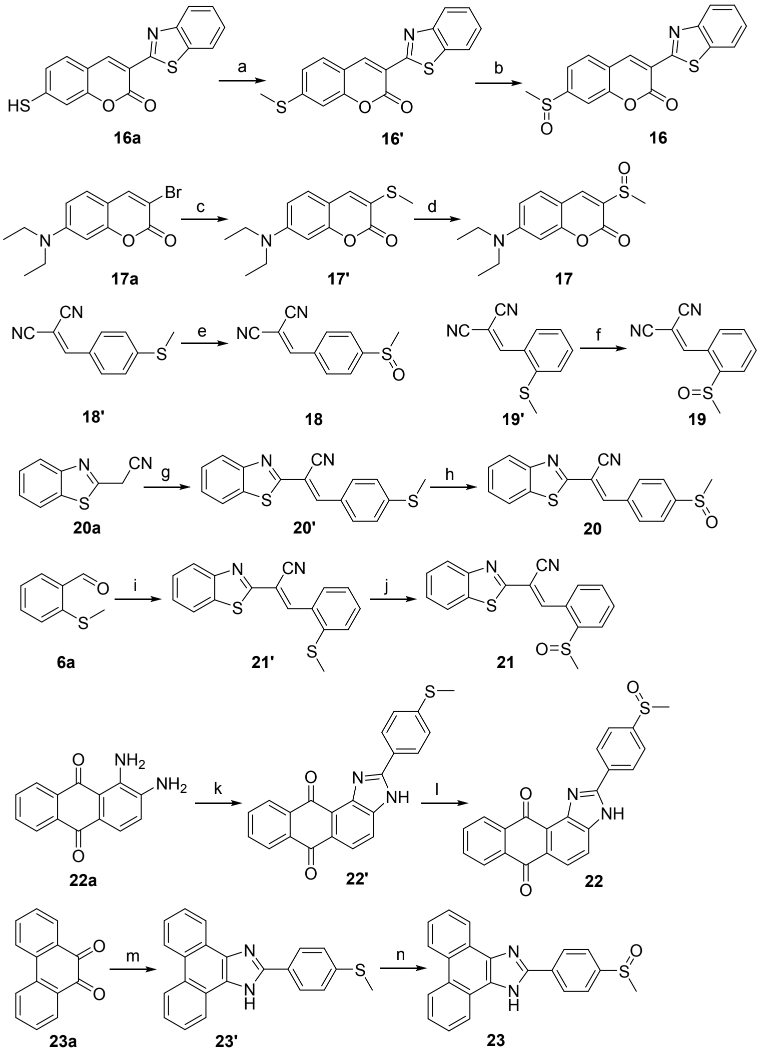

Reagents and conditions: (a) CH_3I/K_2CO_3/Acetone/, rt, 75%; (b) *m*-CPBA/DCM, rt, 86%; (c) DMF/CH_3SNa, rt, 62%; (d) *m*-CPBA/DCM, rt, 81%; (e) *m*-CPBA/DCM, rt, 87%; (f) *m*-CPBA/DCM, rt, 83%; (g) Piperidine/EtOH, rt, 80%; (h) *m*-CPBA/DCM, rt, 92%; (i) Piperidine/EtOH, rt, 78%; (j) *m*-CPBA/DCM, rt, 90%; (k) TFA/DMF, 46%; (l) *m*-CPBA/DCM, rt, 67%; (m) Ammonium acetate/HOAc, 100℃, 70%; (n) *m*-CPBA/DCM, rt, 89%.

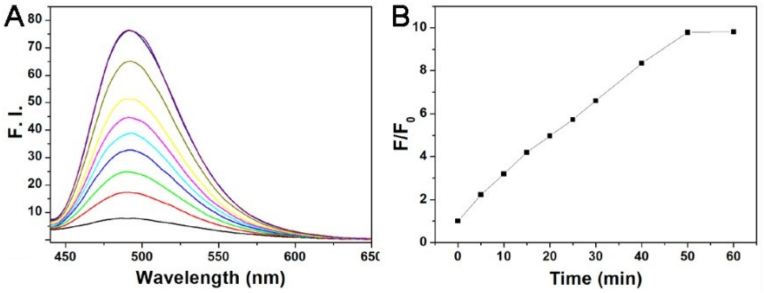

Figure S1. Time-dependent activation of the fluorescence signal of **16** by Msr A. (A) Compound **16** (10 μM) was incubated with DTT (5 mM) and Msr A (3 μg/ml) at 37 °C in TE buffer. The emission spectra (λ_{ex}=420 nm) were recorded for 1 h. (B) The fold of fluorescence increase of **16** at 490 nm from (A) was plotted against time.

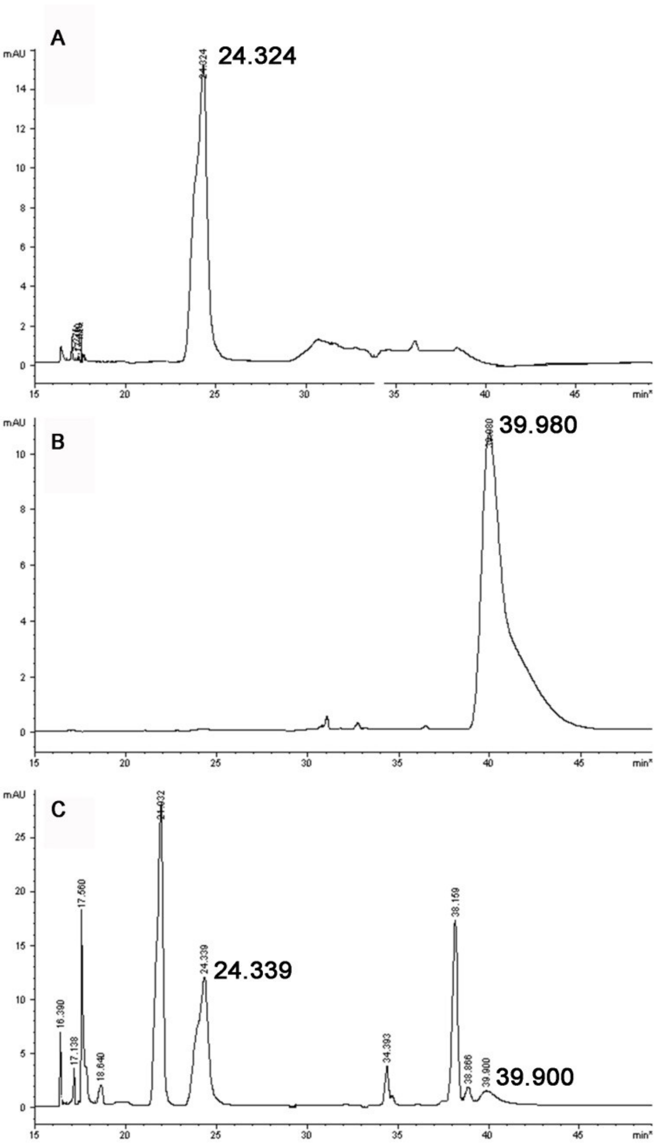

Figure S2. HPLC analyses of the reaction of mouse kidney lysate-mediated Msr-blue reduction. Chromatograms of (A) Msr-blue (10 μM, detected at 278 nm), (B) compound **15'** (10 μM, detected at 333 nm), and (C) the reaction mixture of Msr-blue (10 μM) with mouse kidney lysate (50 μL, 15 mg/mL) and DTT (5 mM) at 37 ^{o}C for 6 h (1-12min, detected at 278 nm; 12-27min detected at 333 nm). The reaction mixture was dried under reduced pressure, and the residue was extracted by ethyl acetate. The combined solvent was removed under vacuum and the residue was reconstituted in the small volume of methanol. All samples were passed through a 0.22 μm filter, and 20 μL of sample was loaded onto Agilent ZORBAX SB-C18, reversed-phase column (5 μm, 4.6×150 mm) on an Agilent 1100 series HPLC system. The column was eluted with methanol/water (1-10 min 35:65, 12-20min 60:40, 22-34 min 35:65). The flow rate was set at 0.6 mL min^{-1}.

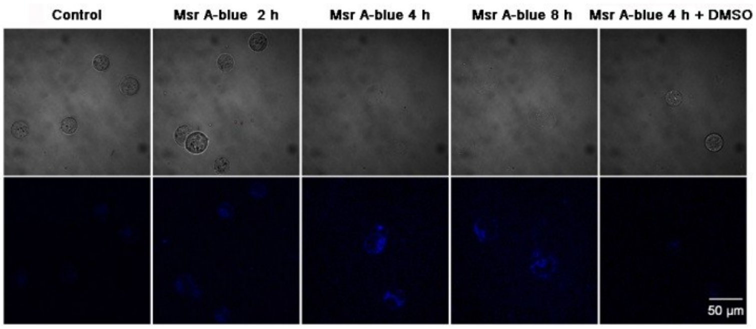

Figure S3. Imaging Msrs activity in live cells and its inhibition by DMSO. HL-60 cells were incubated with Msr-blue (10 μM) for the indicated times, and the pictures were acquired with a confocal fluorescent microscope. To inhibit Msrs activity, the cells was first treated with DMSO (0.2 %) and followed by incubation with the Msr-blue (10 μM) for 4 h.

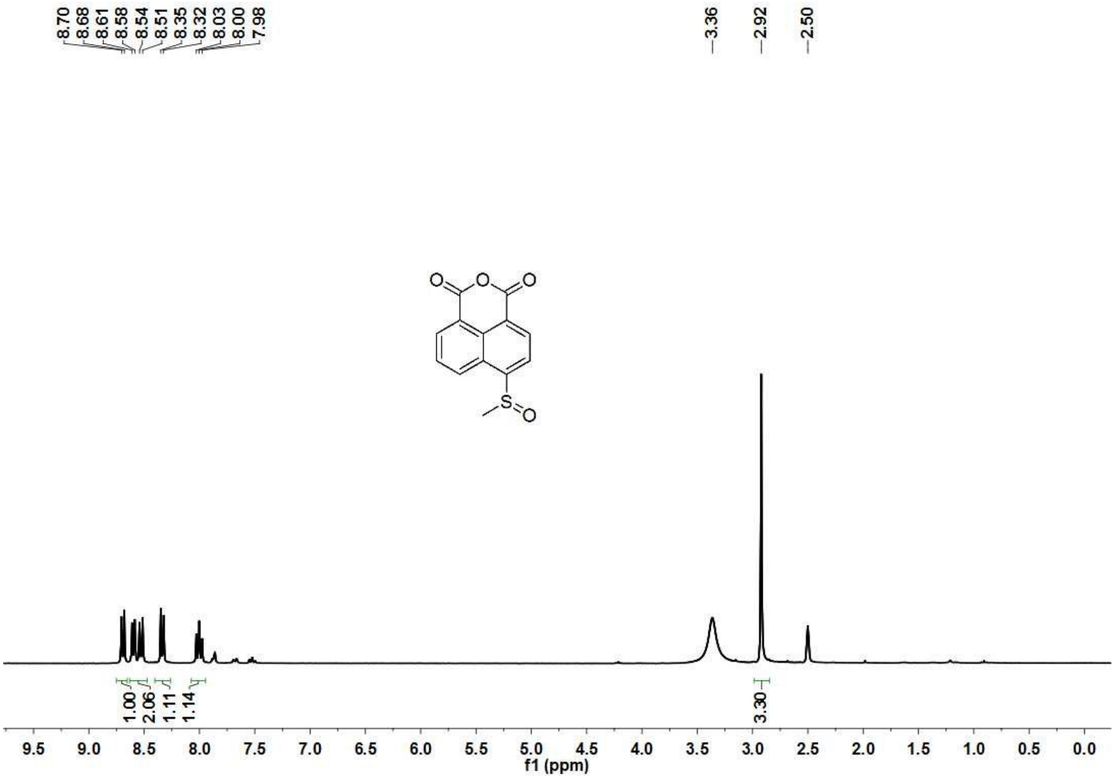

Figure S4. ^{1}H NMR spectra of compoud **1**.

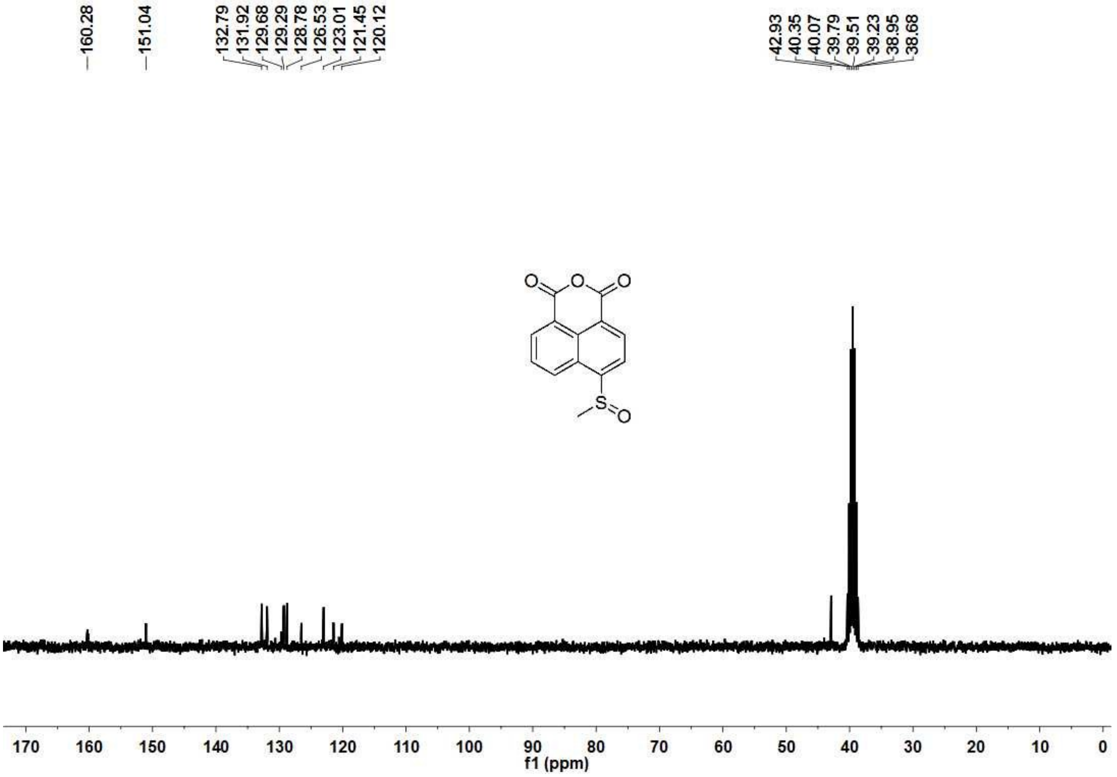

Figure S5. ^{13}C NMR spectra of compoud **1**.

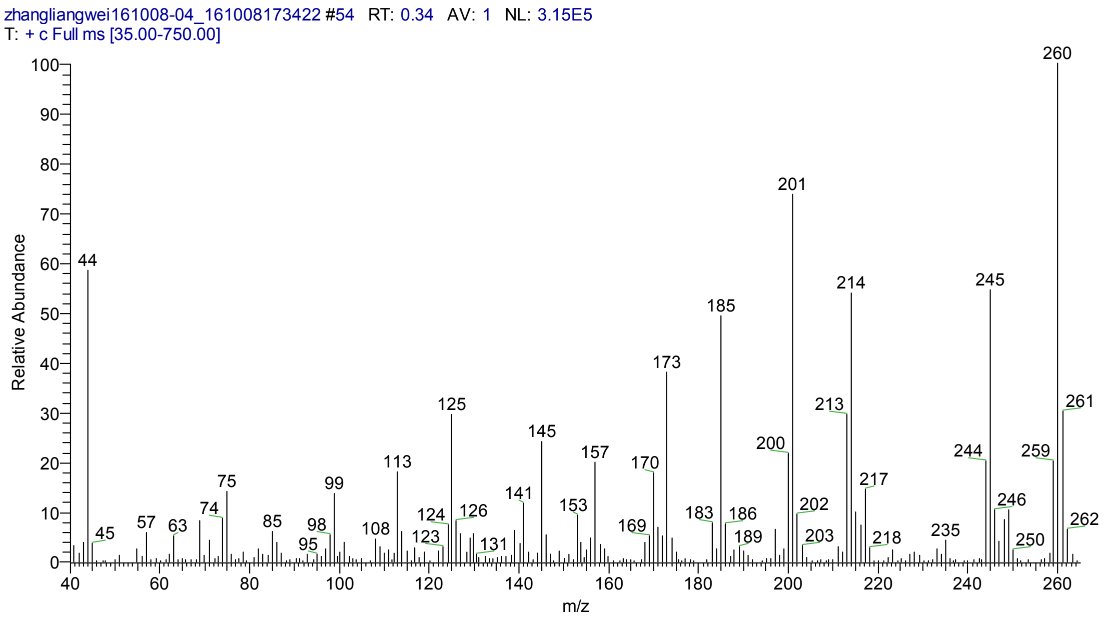

Figure S6. MS spectra of compound **1.**

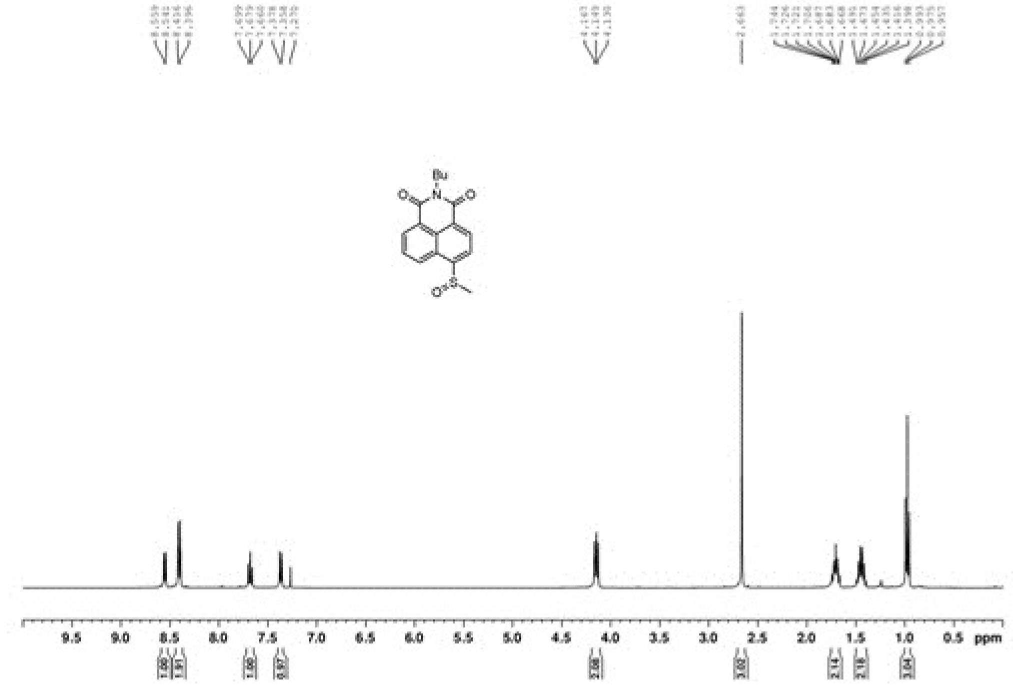

Figure S7. ^{1}H NMR spectra of compoud **2**.

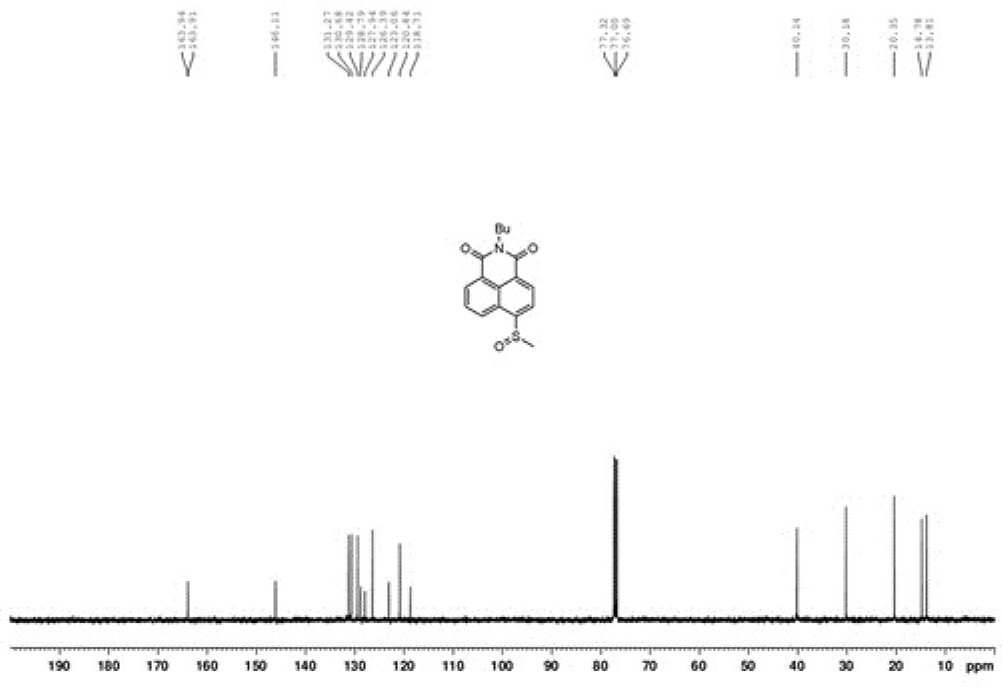

Figure S8. ^{13}C NMR spectra of compoud **2**.

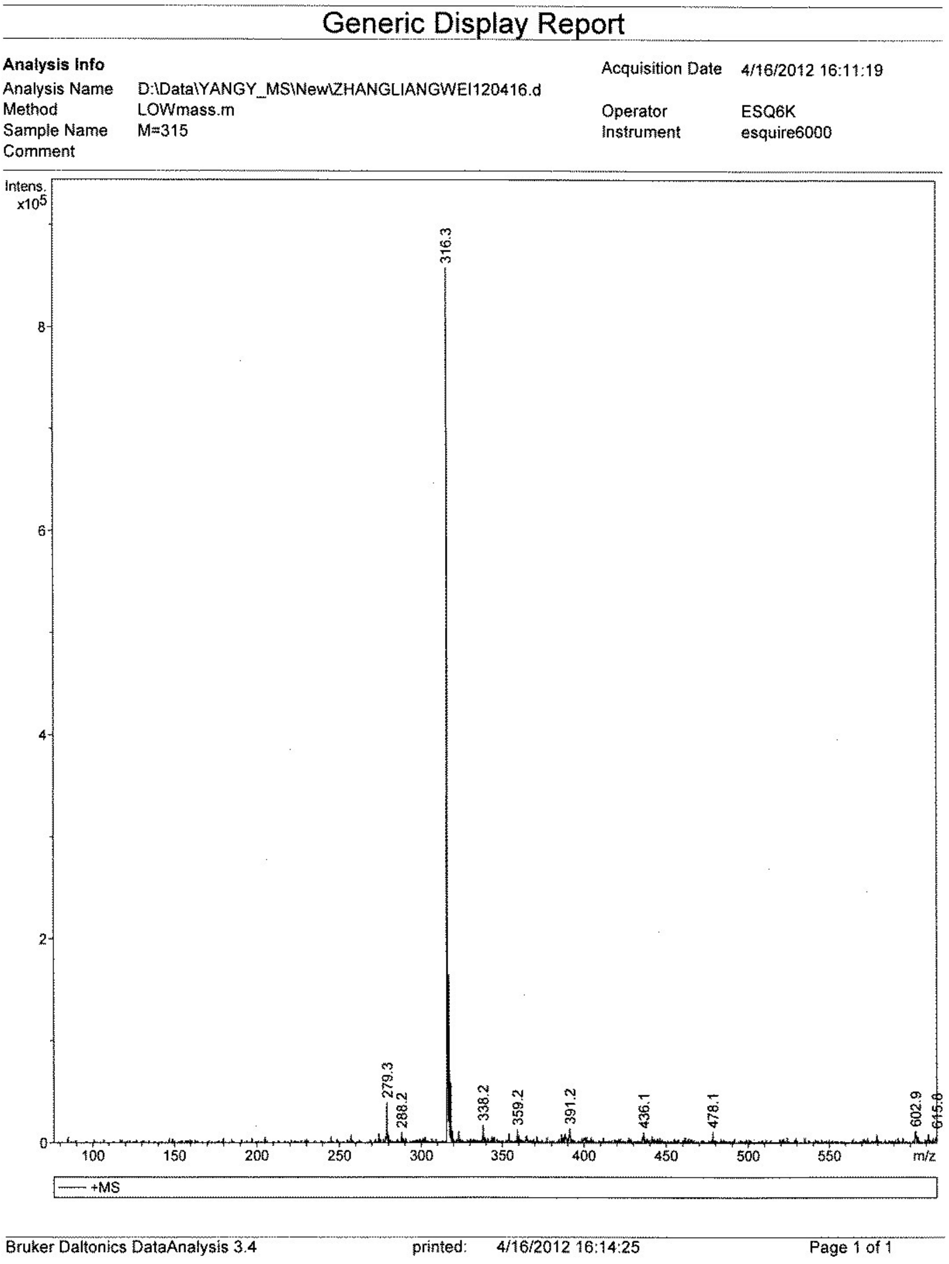

Figure S9. MS spectra of compoud **2**.

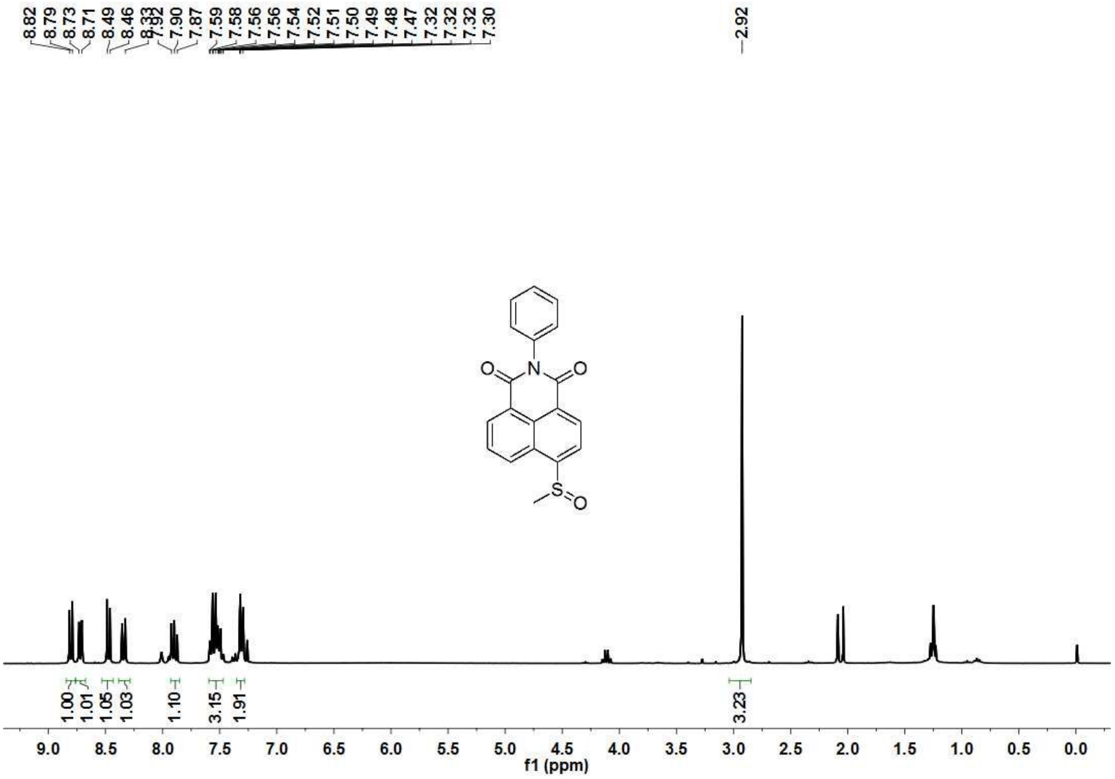

Figure S10. ^{1}H NMR spectra of compoud **3**.

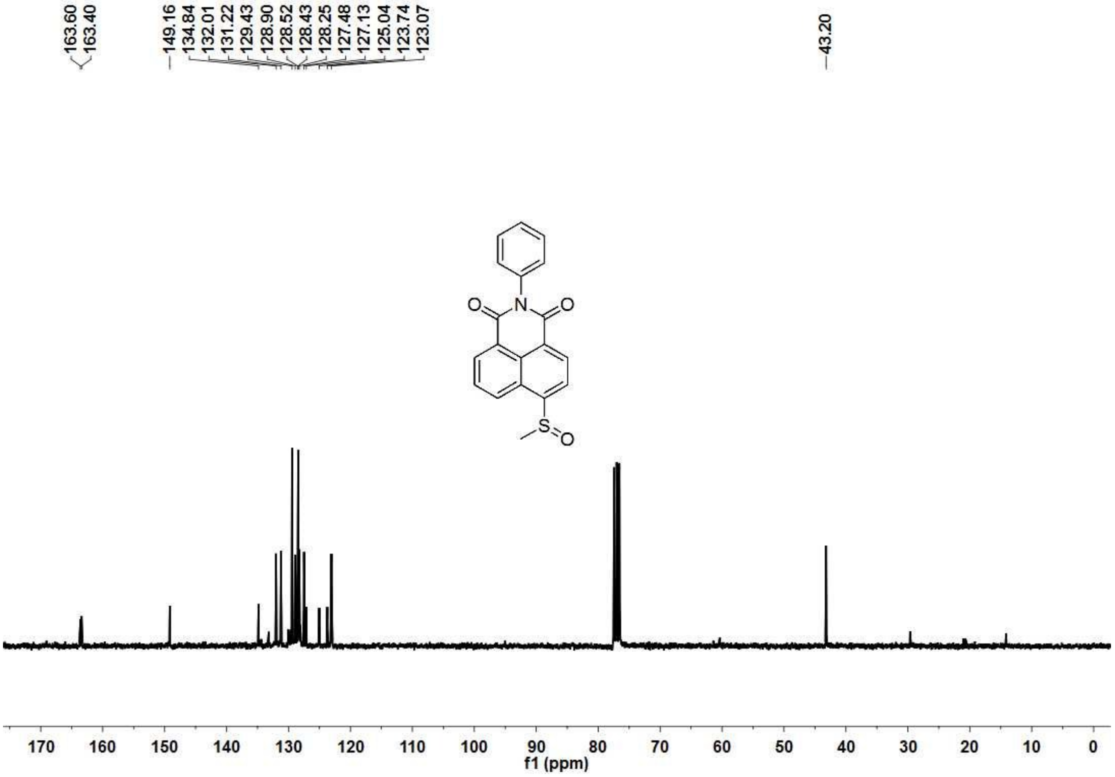

Figure S11. ^{13}C NMR spectra of compoud **3**.

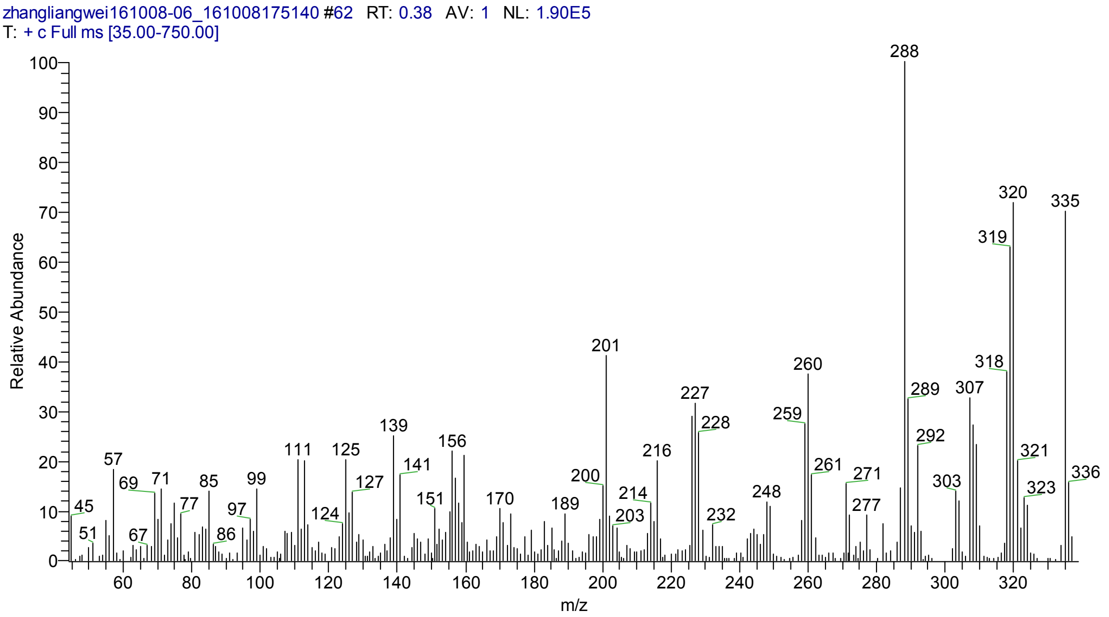

Figure S12. MS spectra of compound **3**.

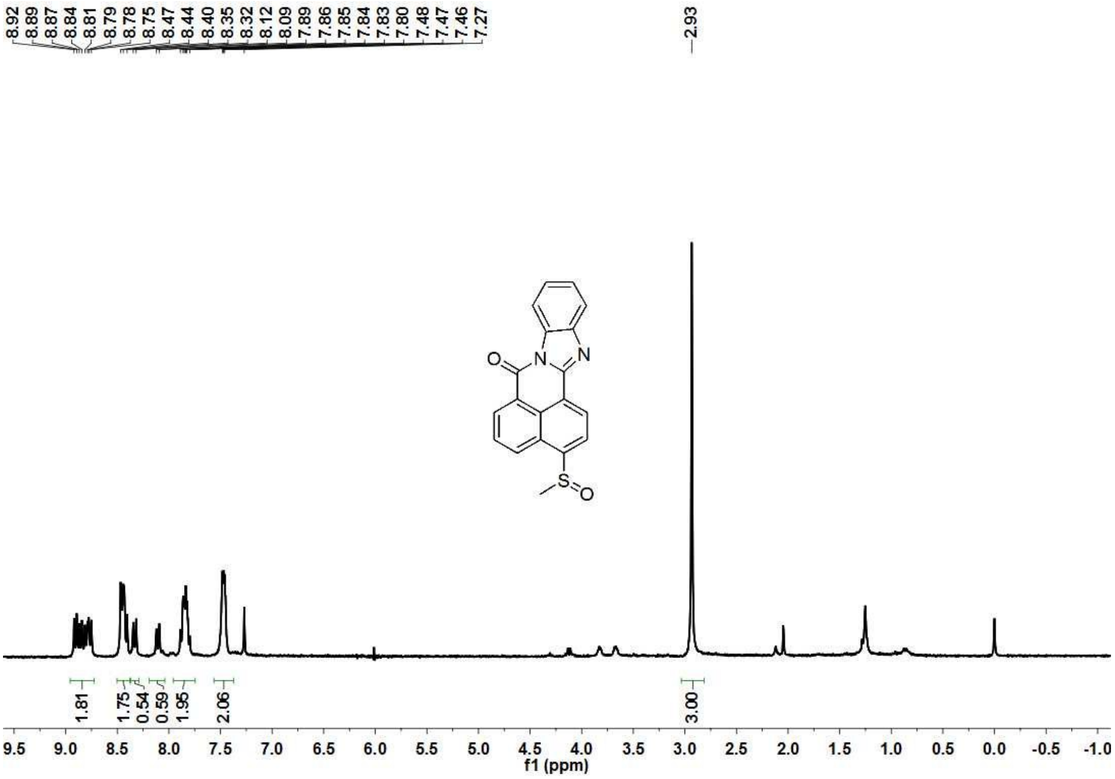

Figure S13. ^{1}H NMR spectra of compoud **4**.

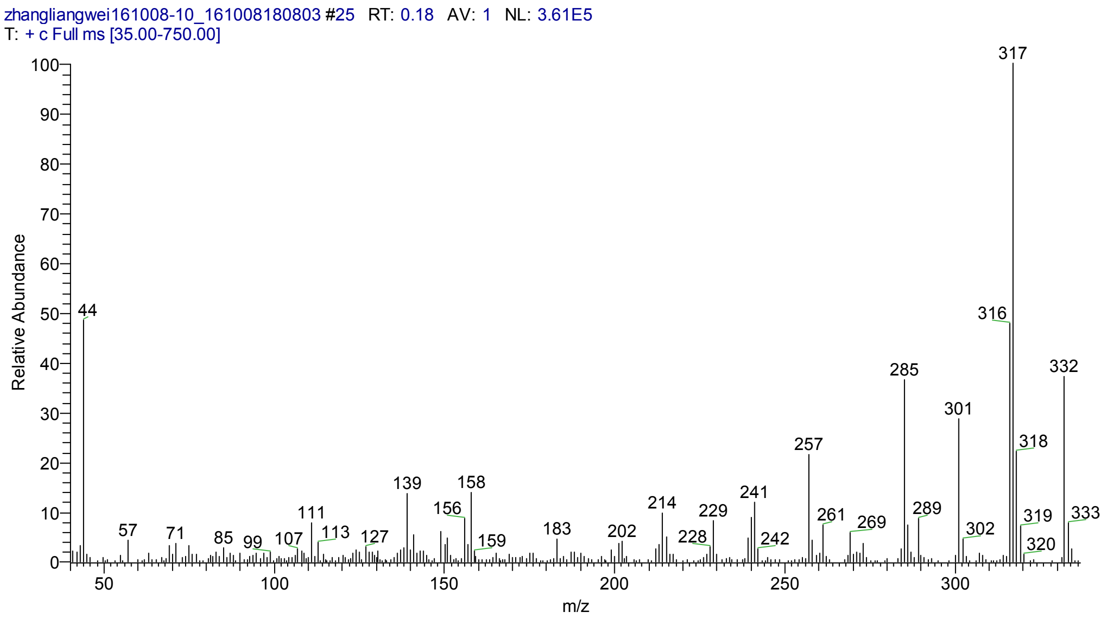

Figure S14. MS spectra of compound **4.**

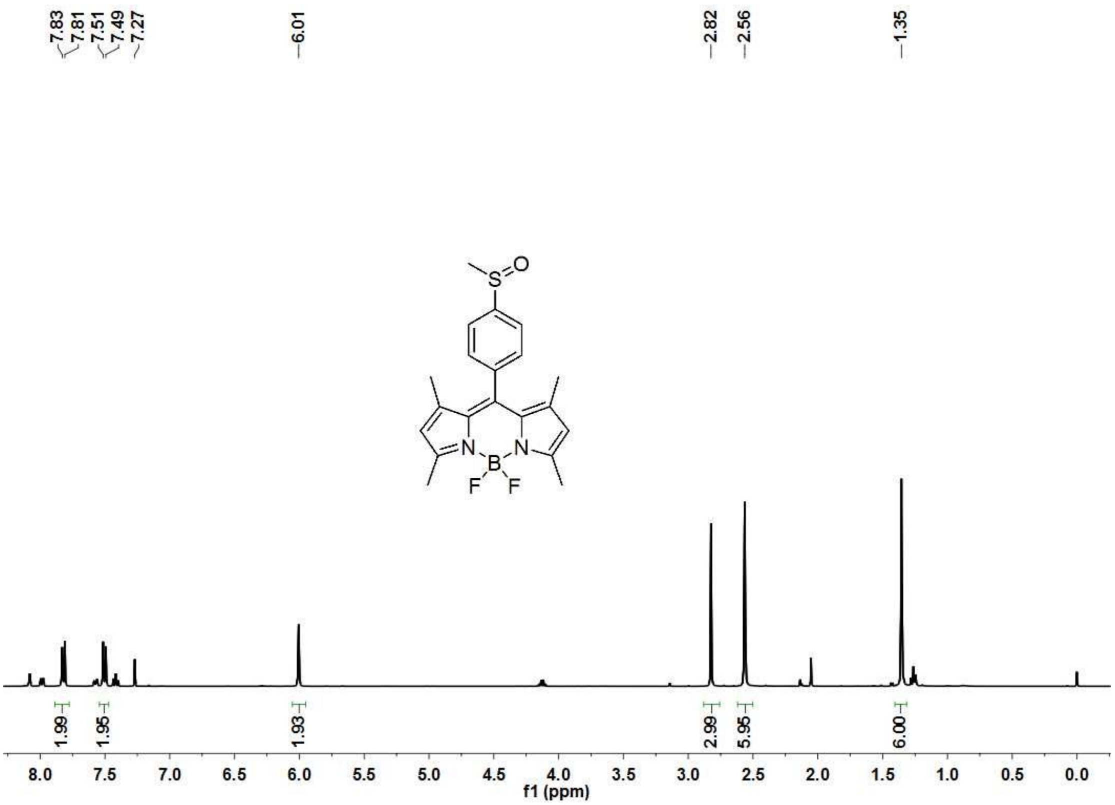

Figure S15. ^{1}H NMR spectra of compoud **5**.

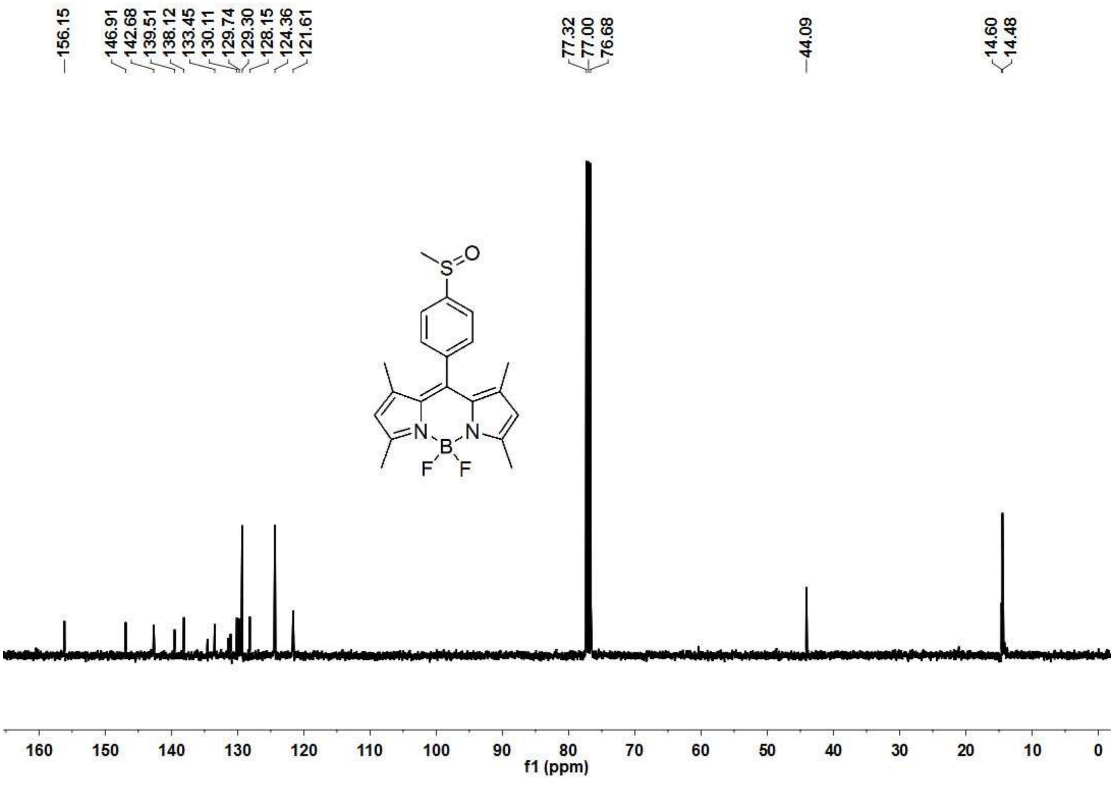

Figure S16. ^{13}C NMR spectra of compoud **5**.

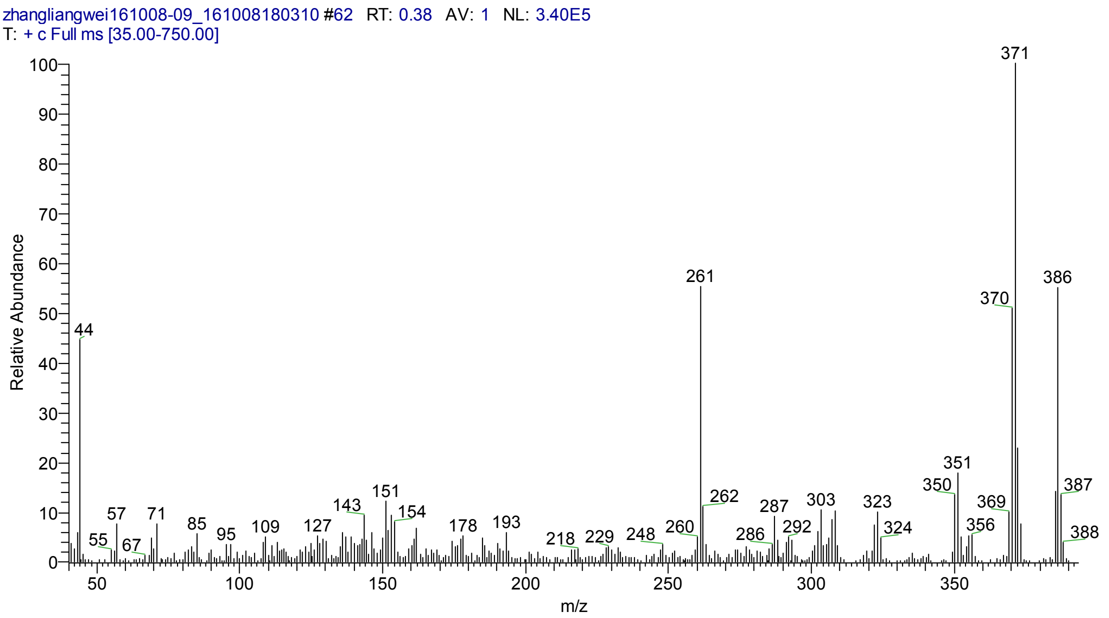

Figure S17. MS spectra of compound **5**

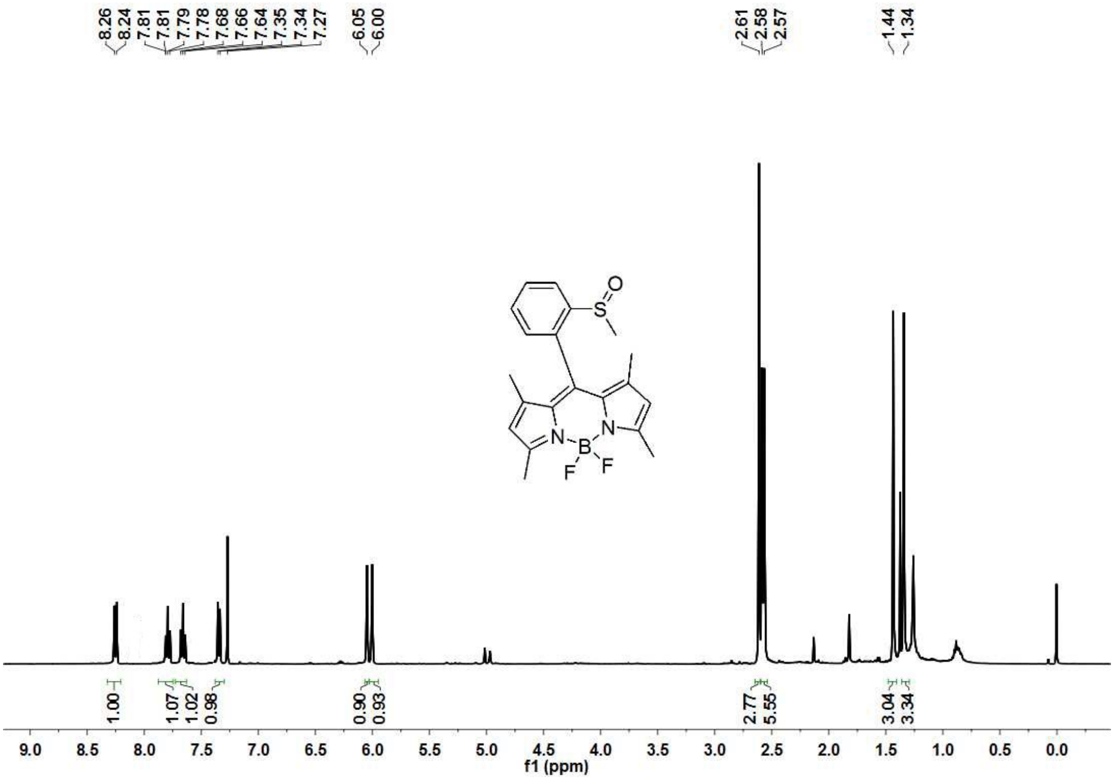

Figure S18. ^{1}H NMR spectra of compoud **6**.

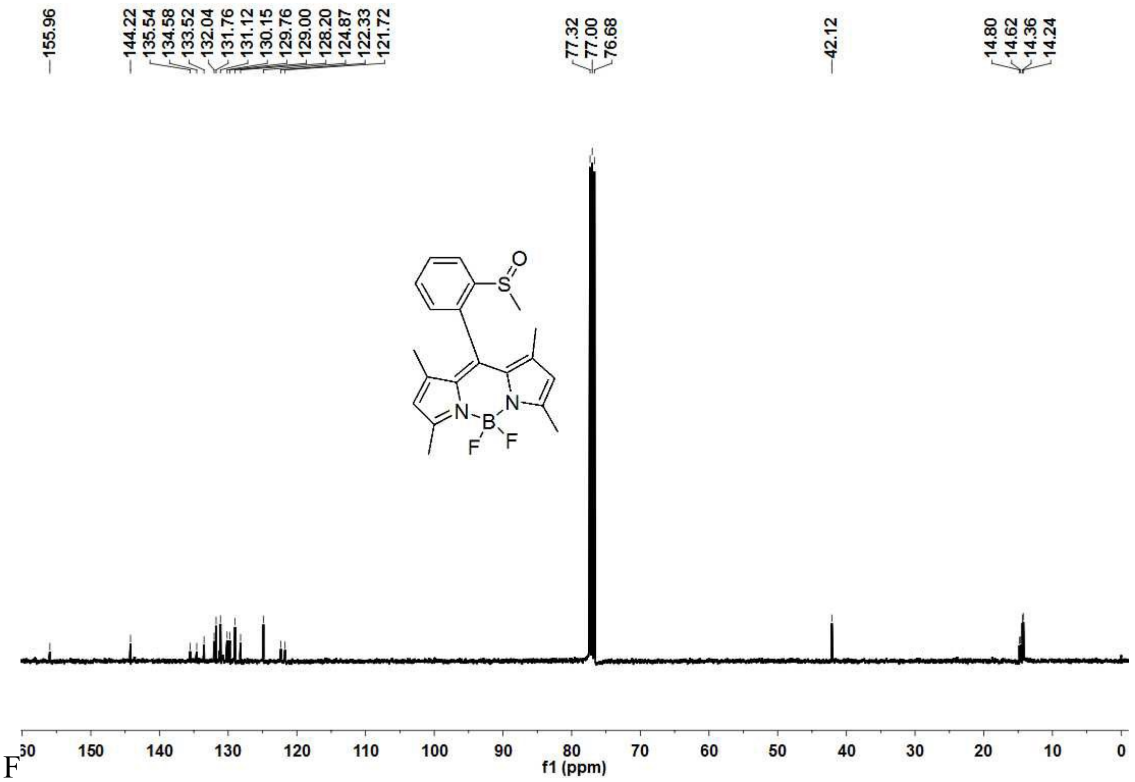

Figure S19. ^{13}C NMR spectra of compoud **6**.

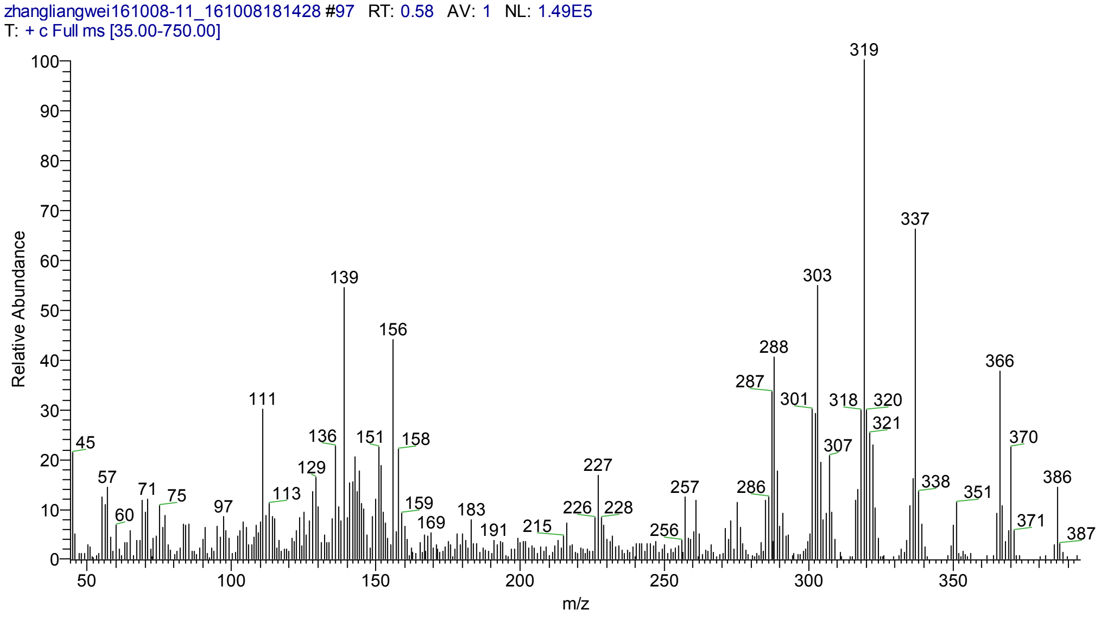

Figure S20. MS spectra of compound **6.**

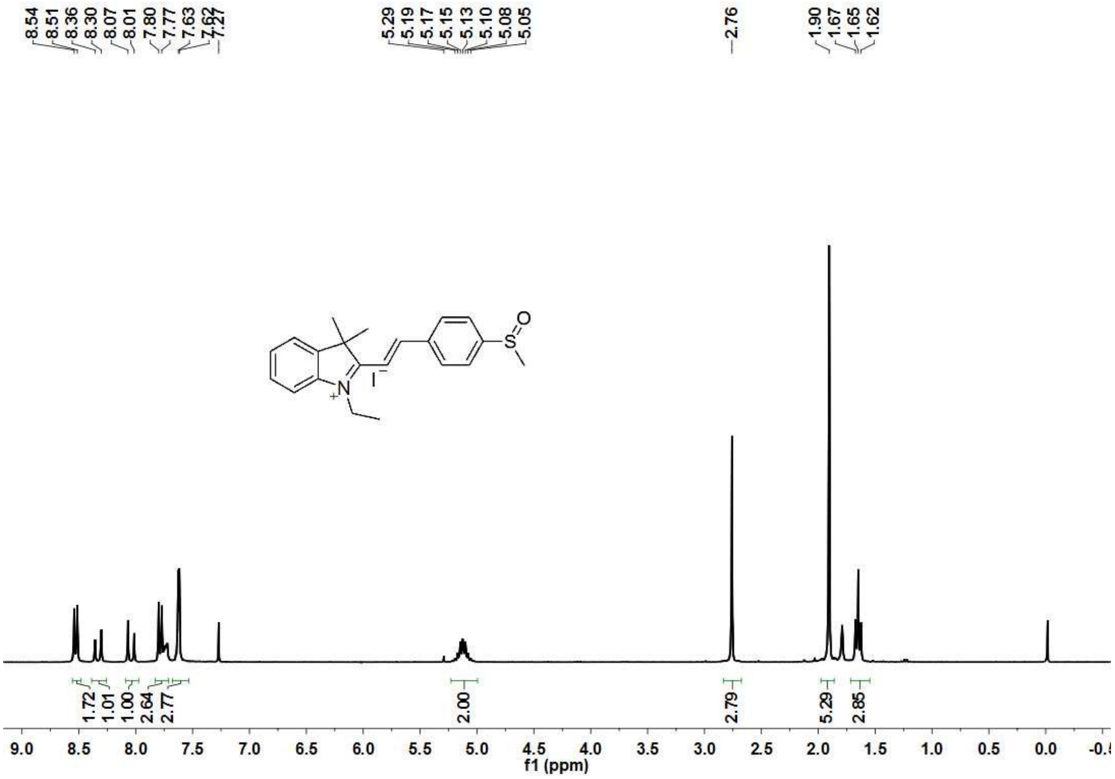

Figure S21. ^{1}H NMR spectra of compoud **7**.

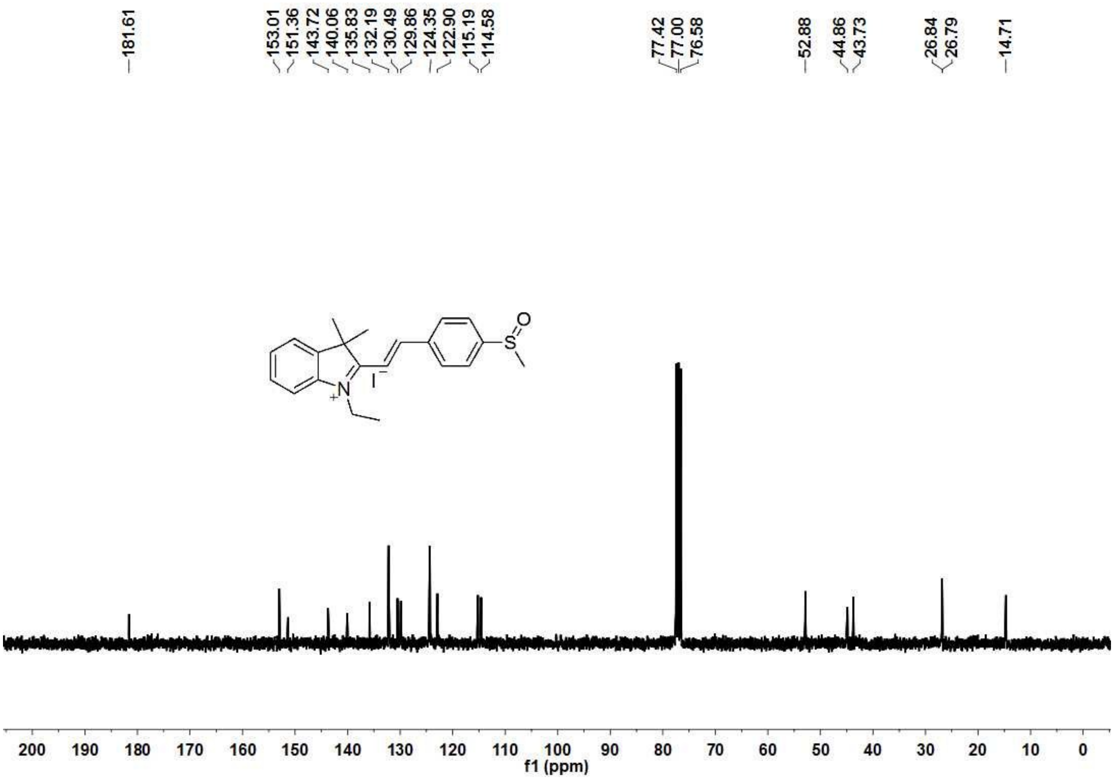

Figure S22. ^{13}C NMR spectra of compoud **7**.

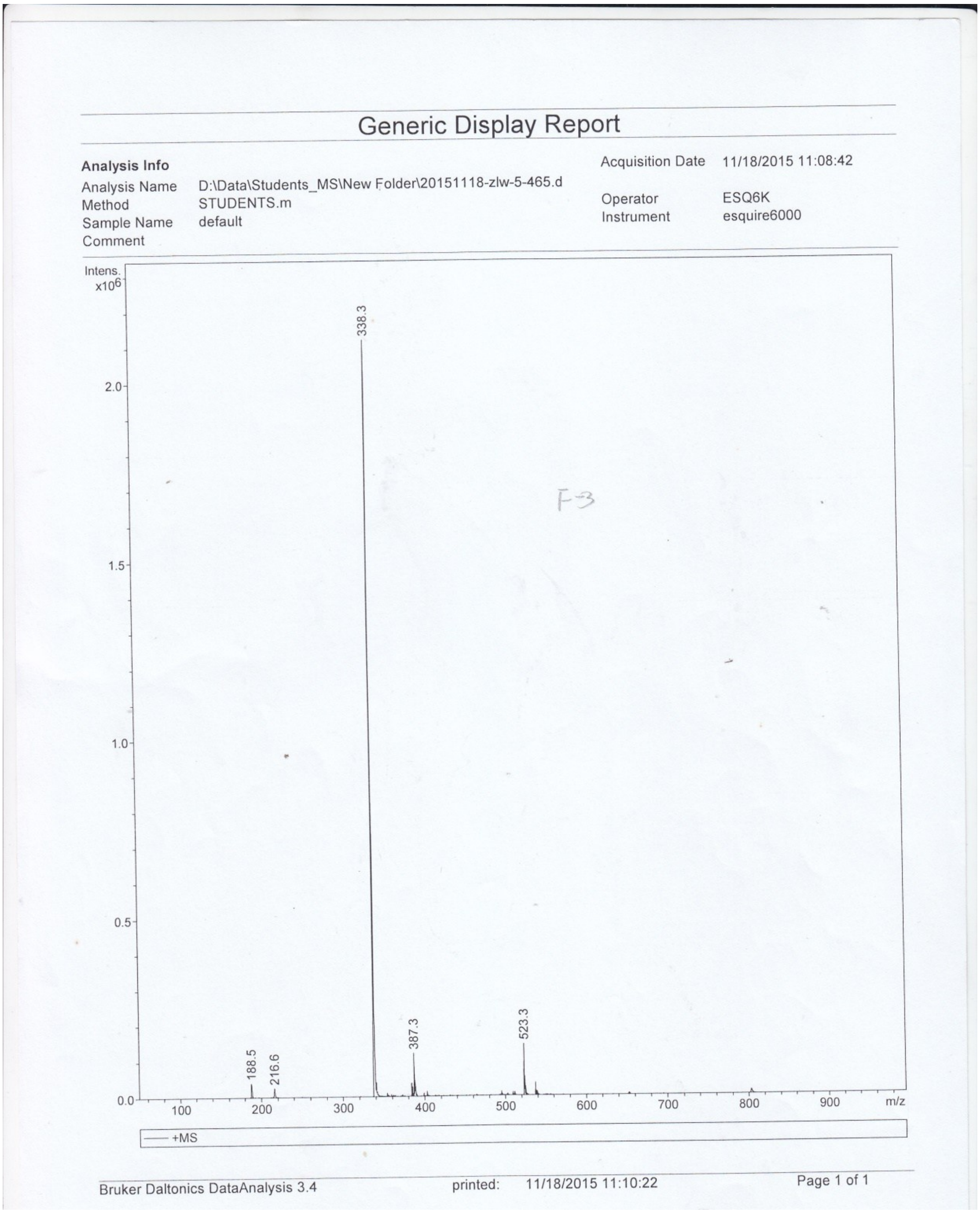

Figure s23. MS spectra of compound **7**.

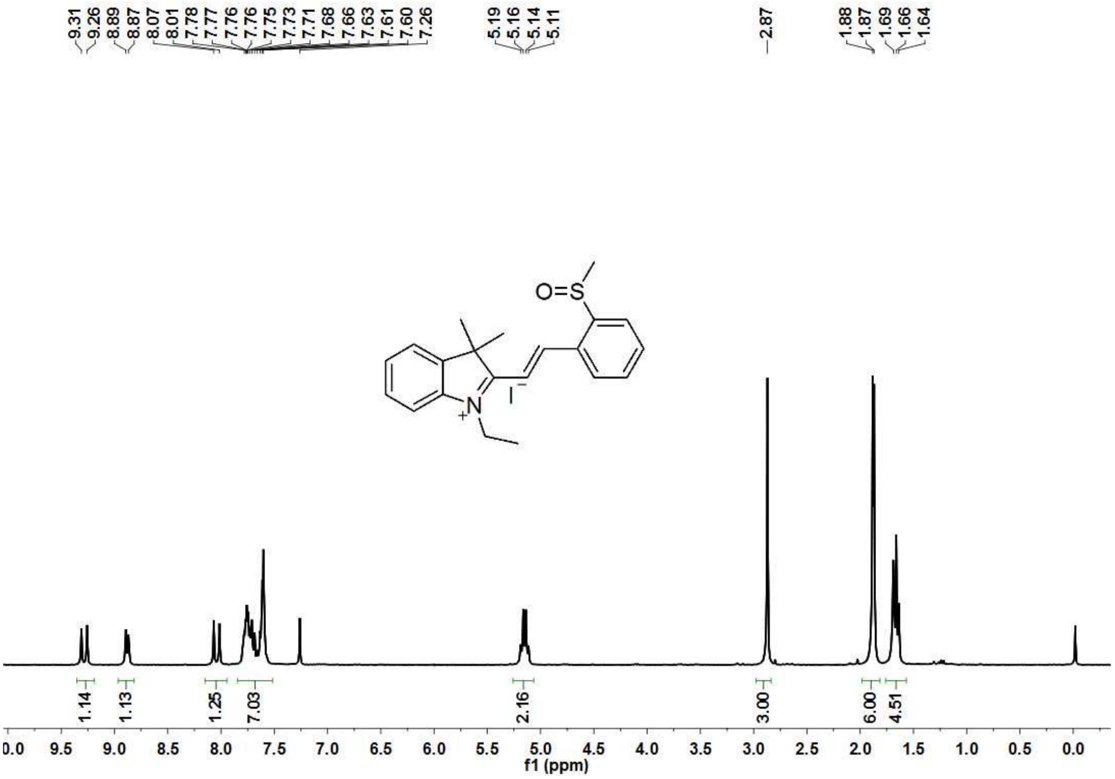

Figure S24. ^{1}H NMR spectra of compoud **8**.

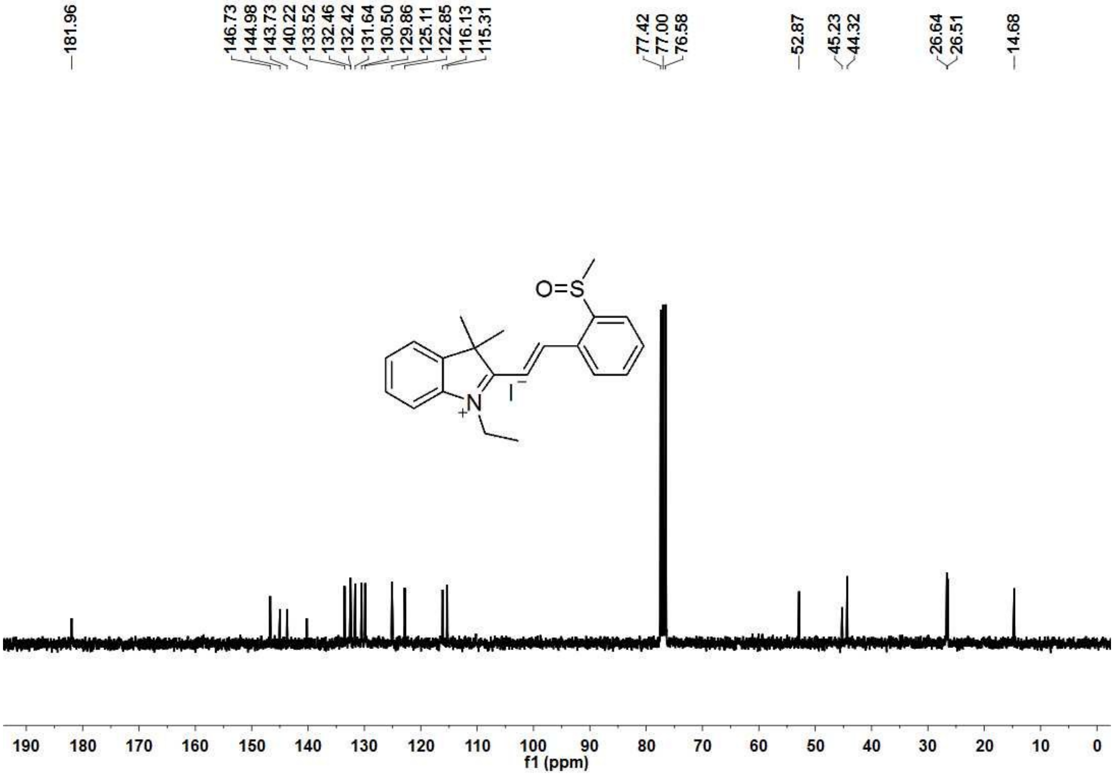

Figure S25. ^{13}C NMR spectra of compoud **8**.

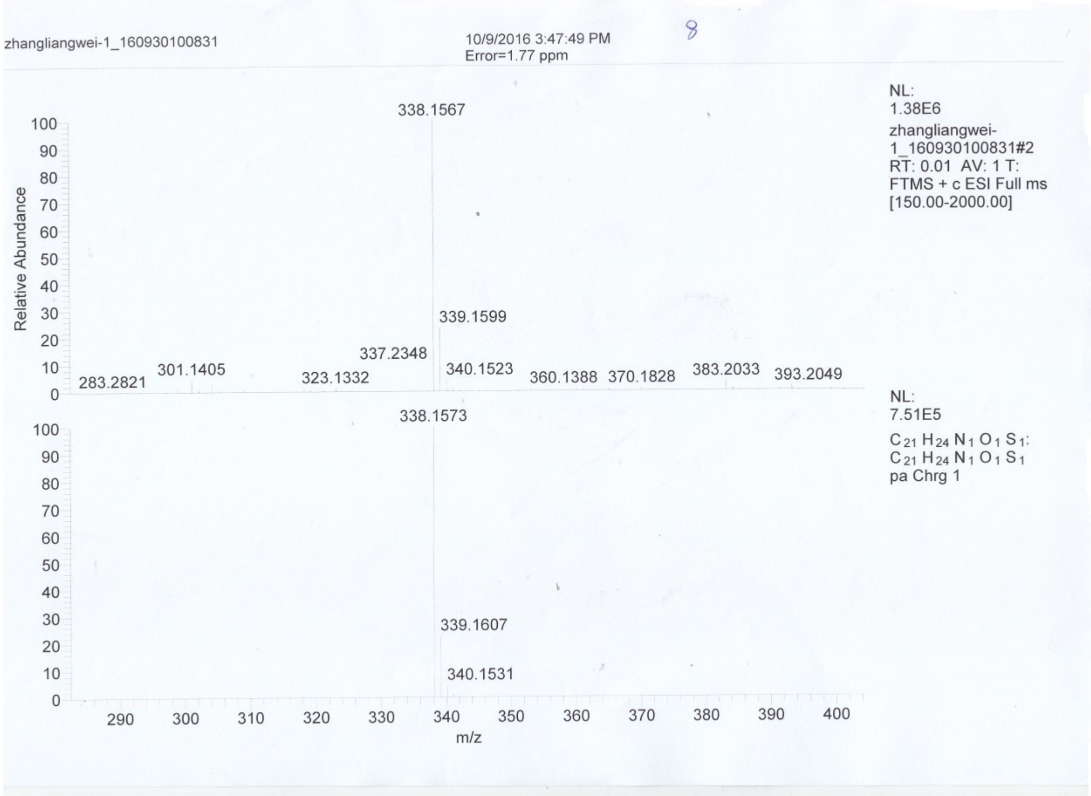

Figure S26. MS spectra of compound **8**.

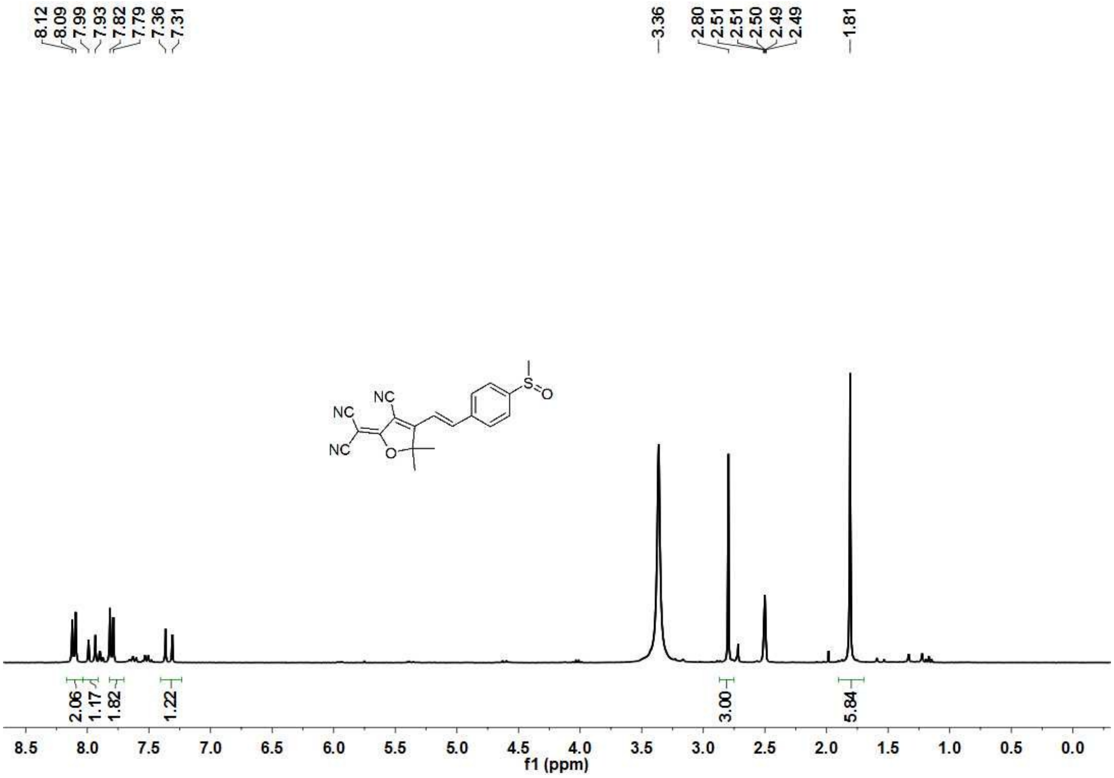

Figure S27. ^{1}H NMR spectra of compoud **9**.

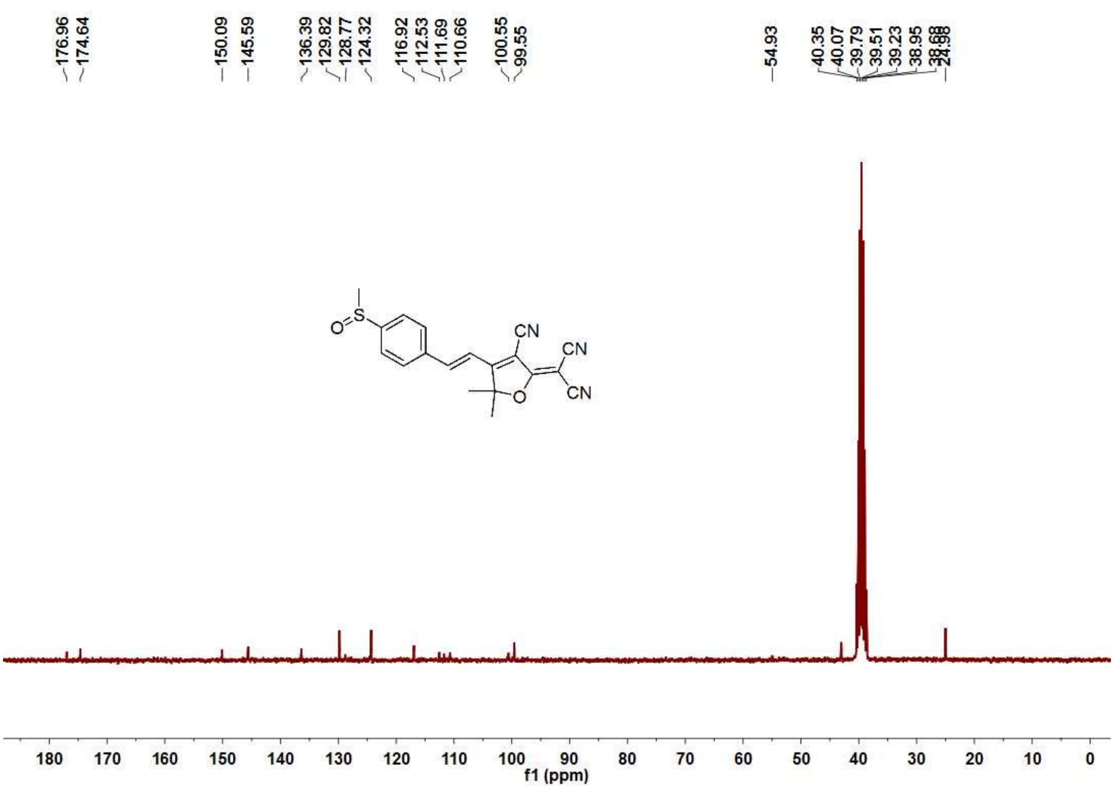

Figure S28. ^{13}C NMR spectra of compoud **9**.

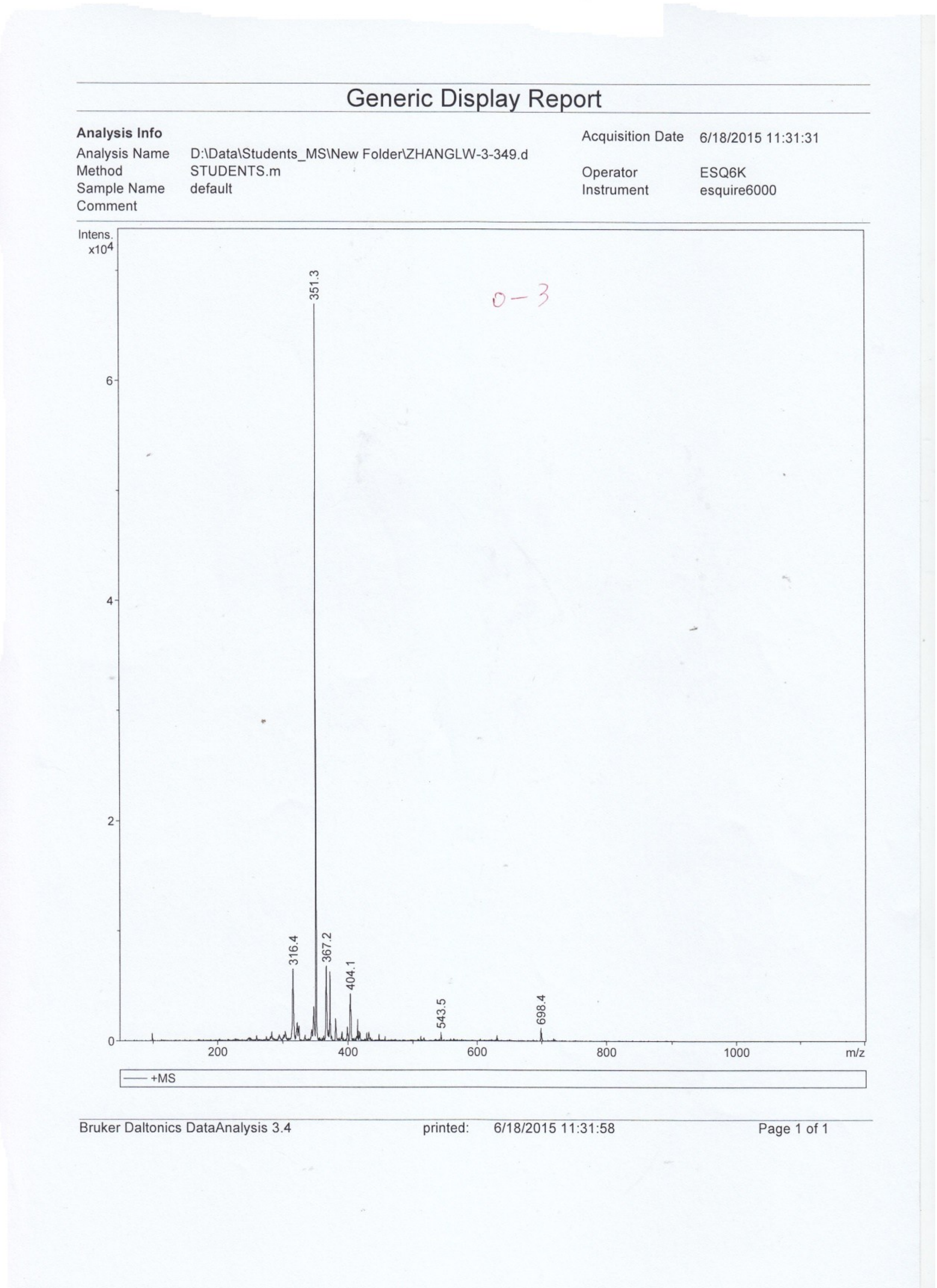

Figure S29. MS spectra of compound **9.**

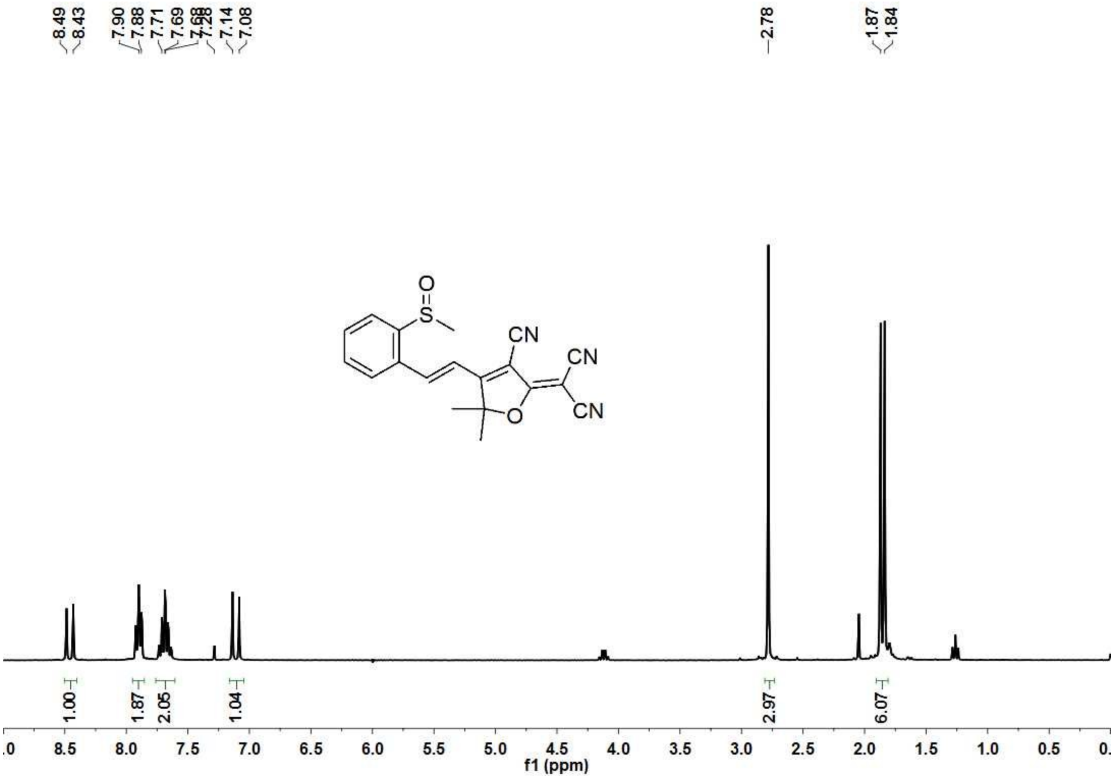

Figure S30. ^{1}H NMR spectra of compoud **10**.

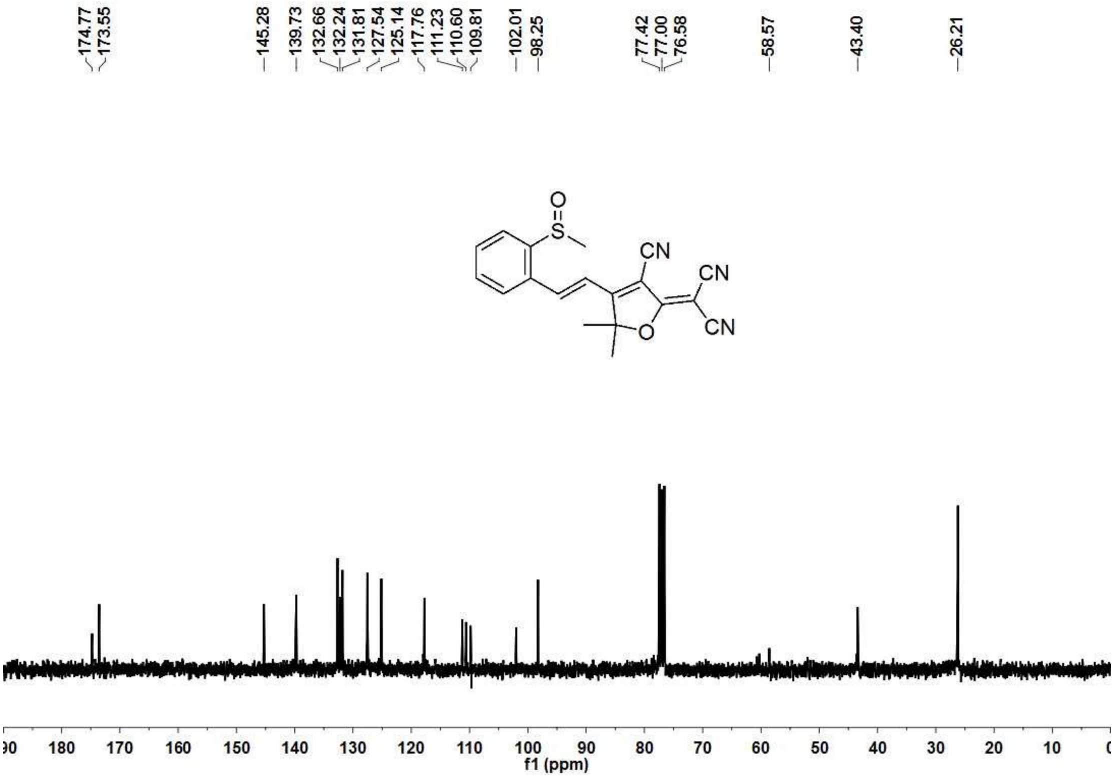

Figure S31. ^{13}C NMR spectra of compoud **10**.

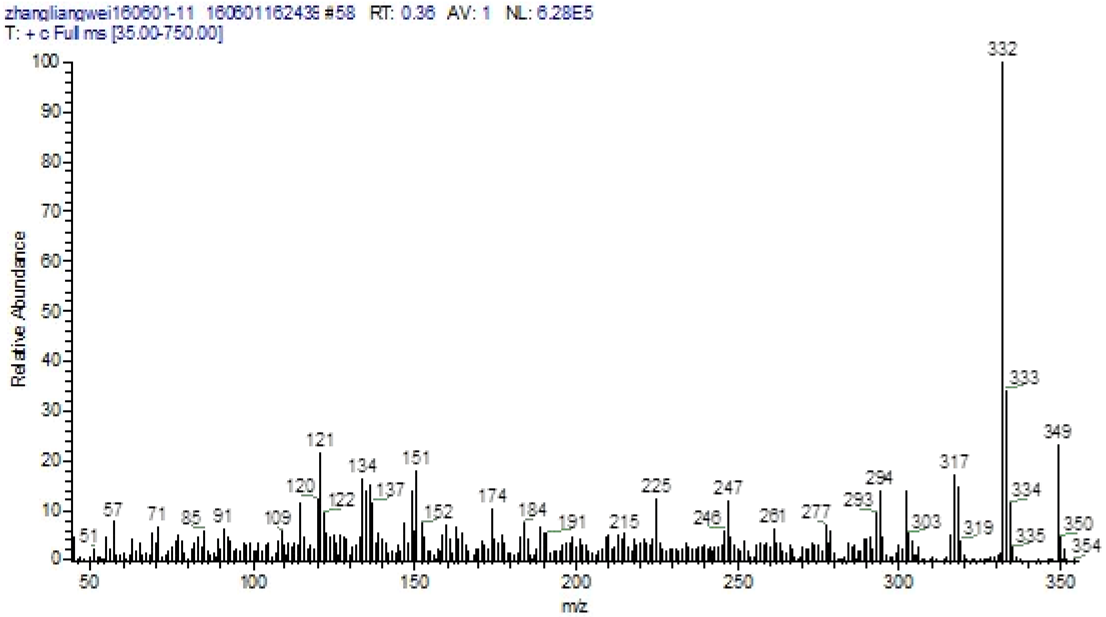

Figure S32. MS spectra of compound **10**.

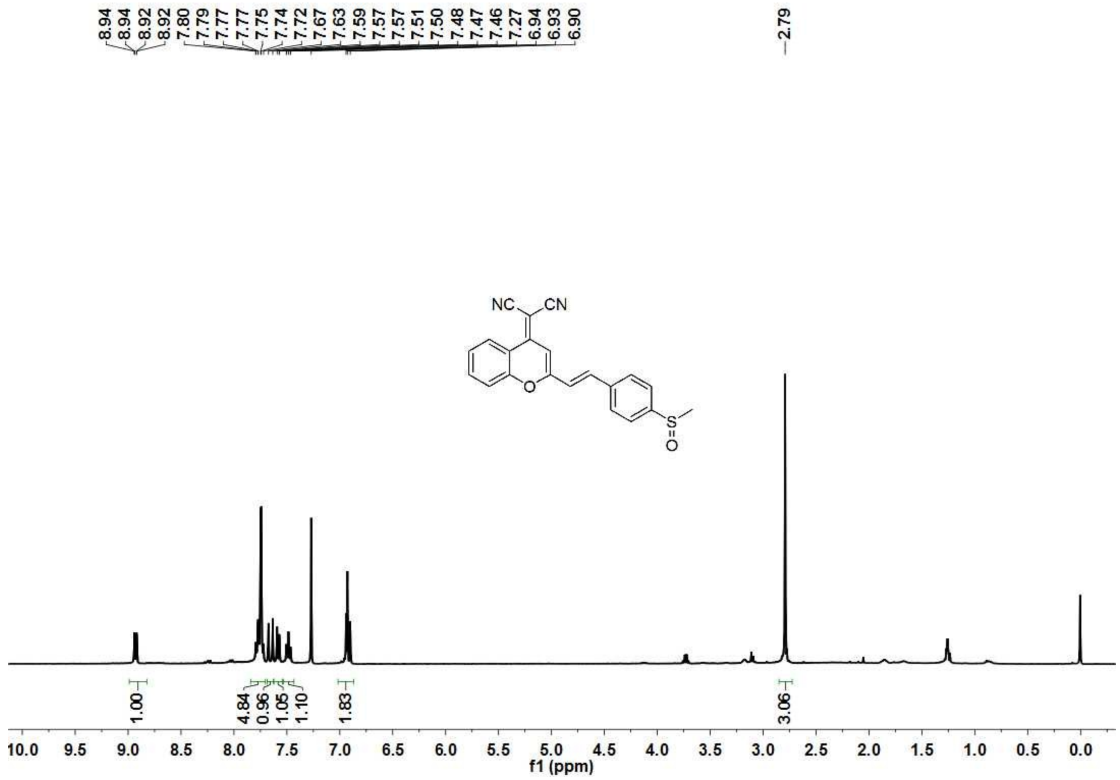

Figure S33. ^{1}H NMR spectra of compoud **11**.

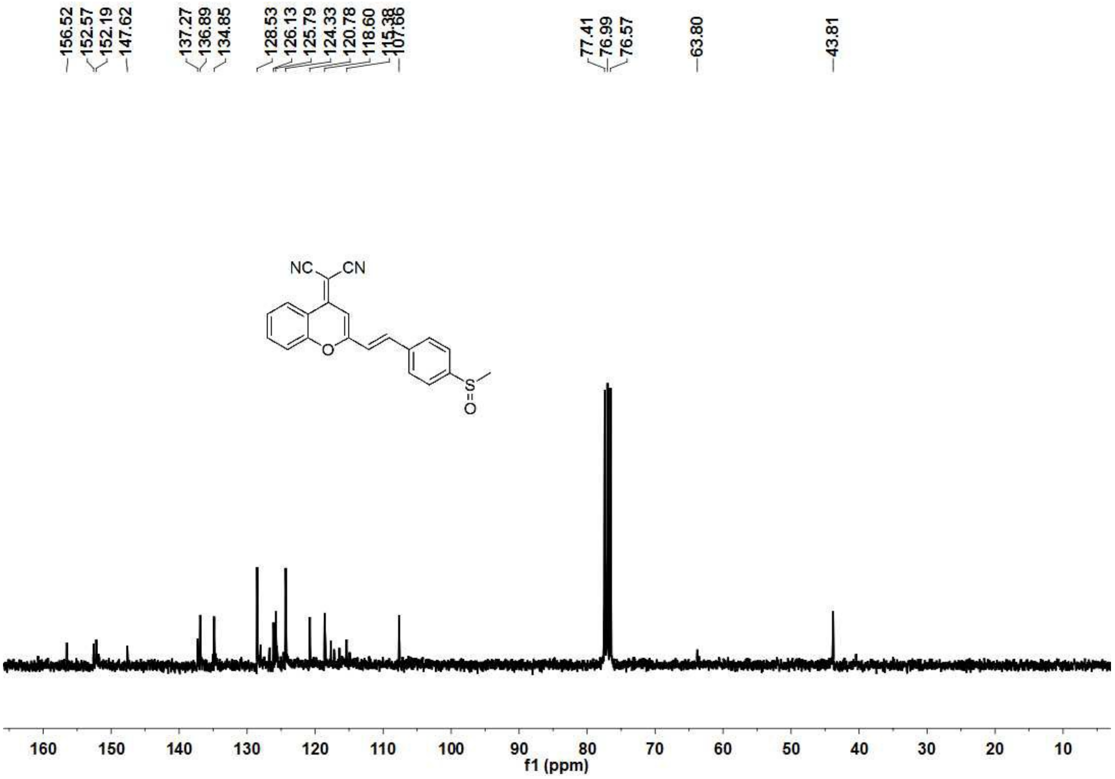

Figure S34. ^{13}C NMR spectra of compoud **11**.

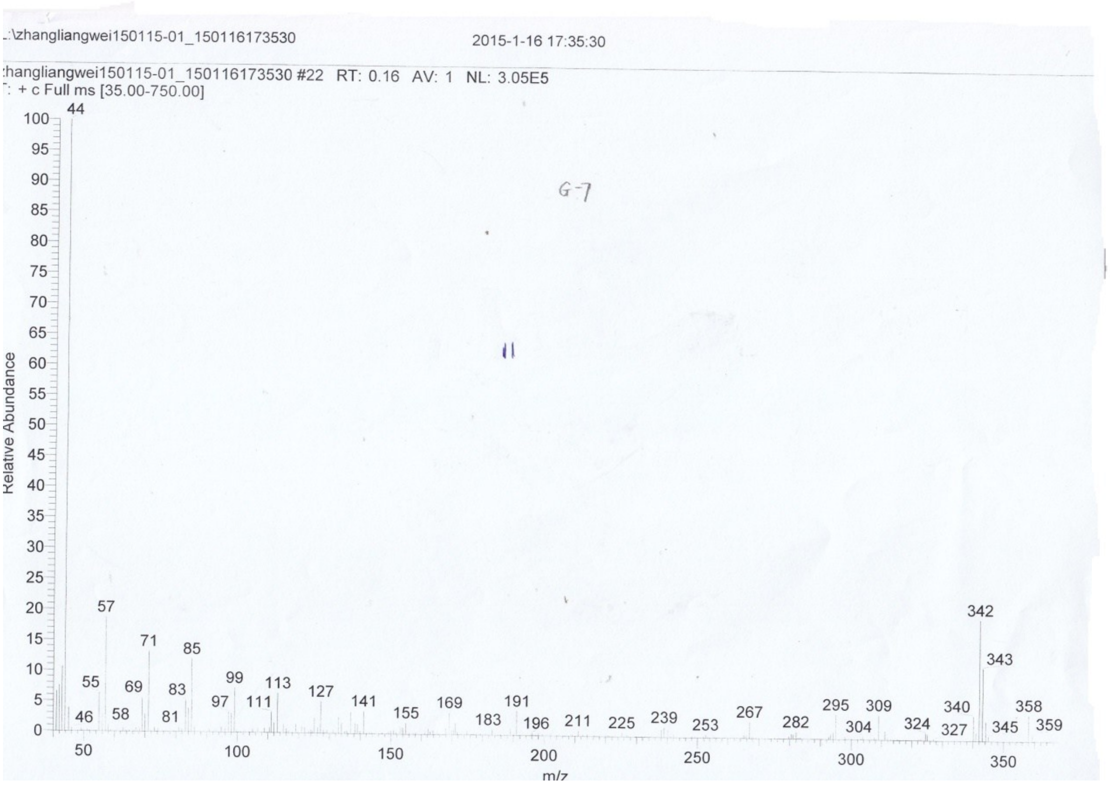

Figure S35. MS spectra of compound **11**.

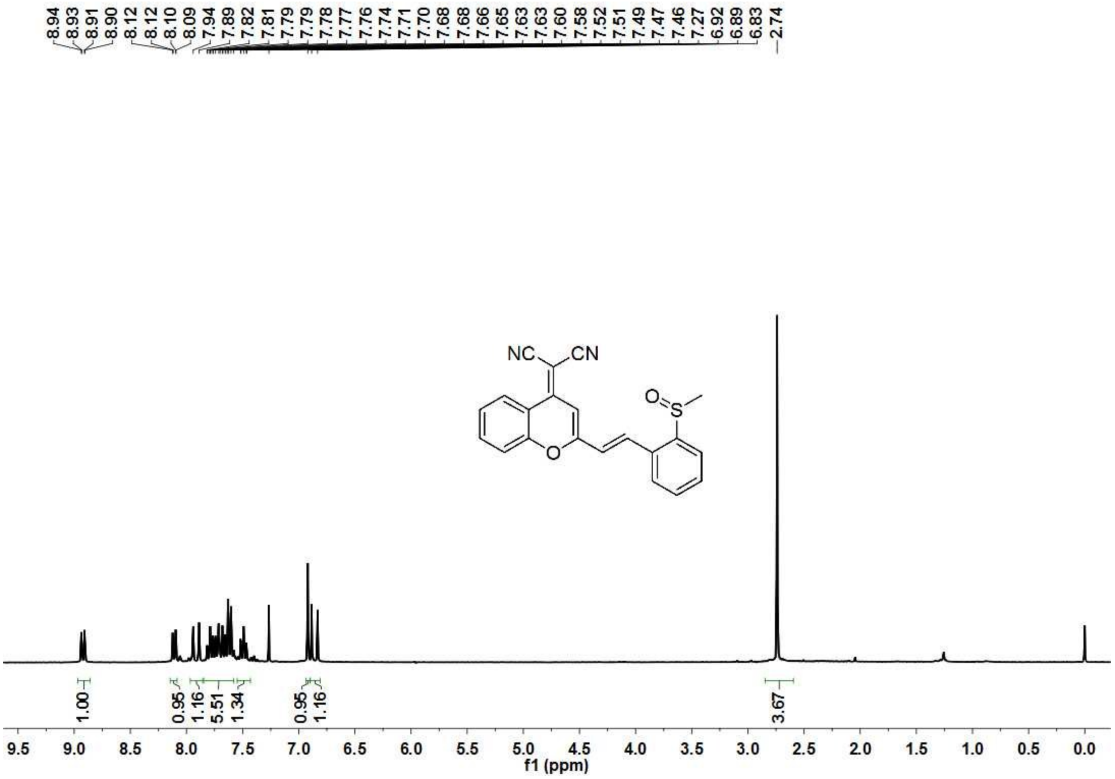

Figure S36. ^{1}H NMR spectra of compoud **12**.

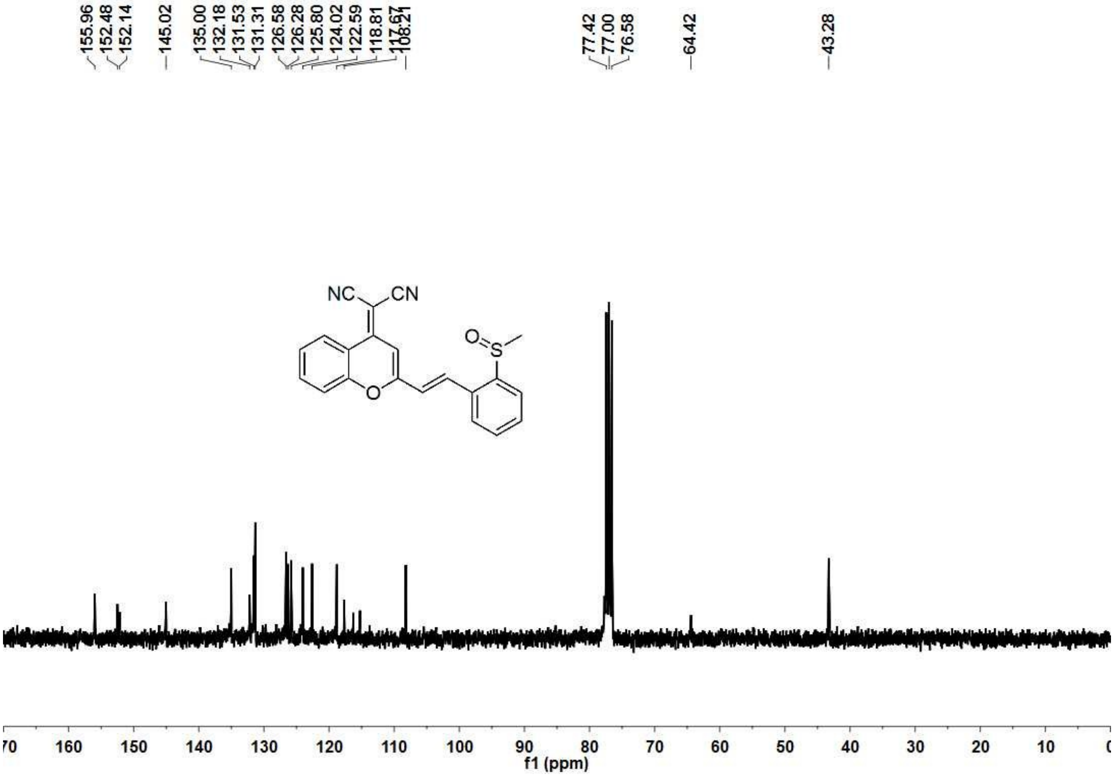

Figure S37. ^{13}C NMR spectra of compoud **12**.

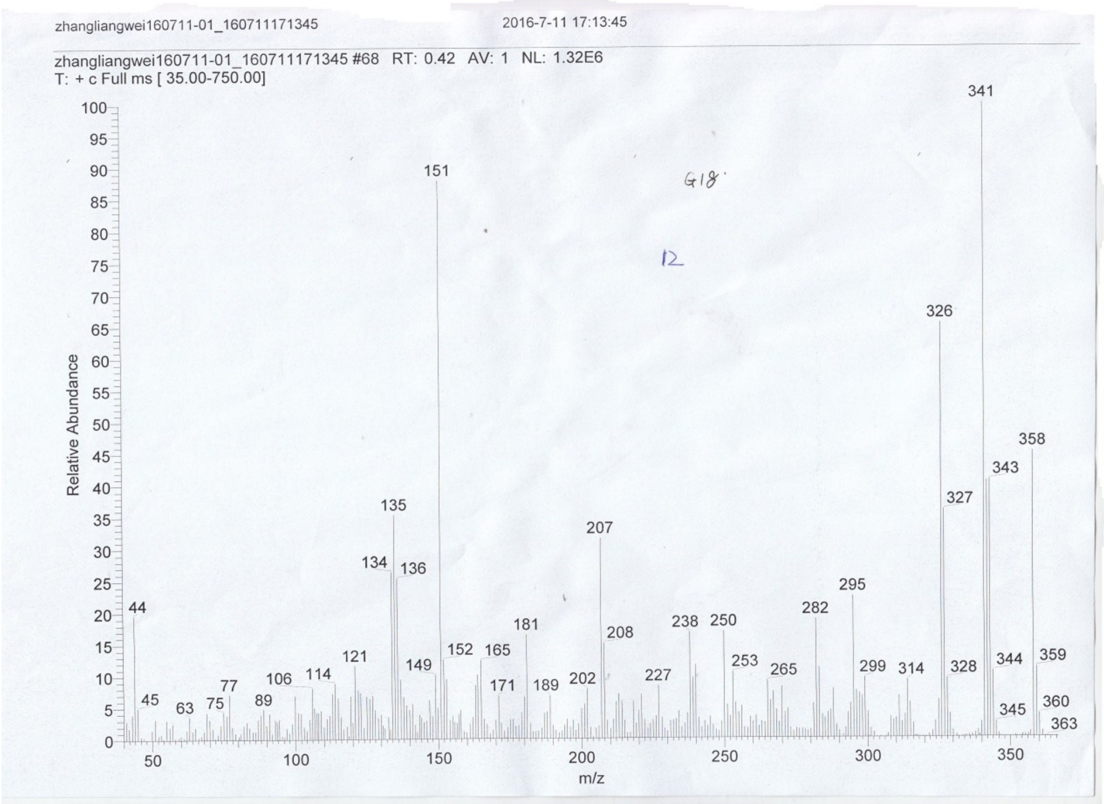

Figure S38. MS spectra of compound **12**.

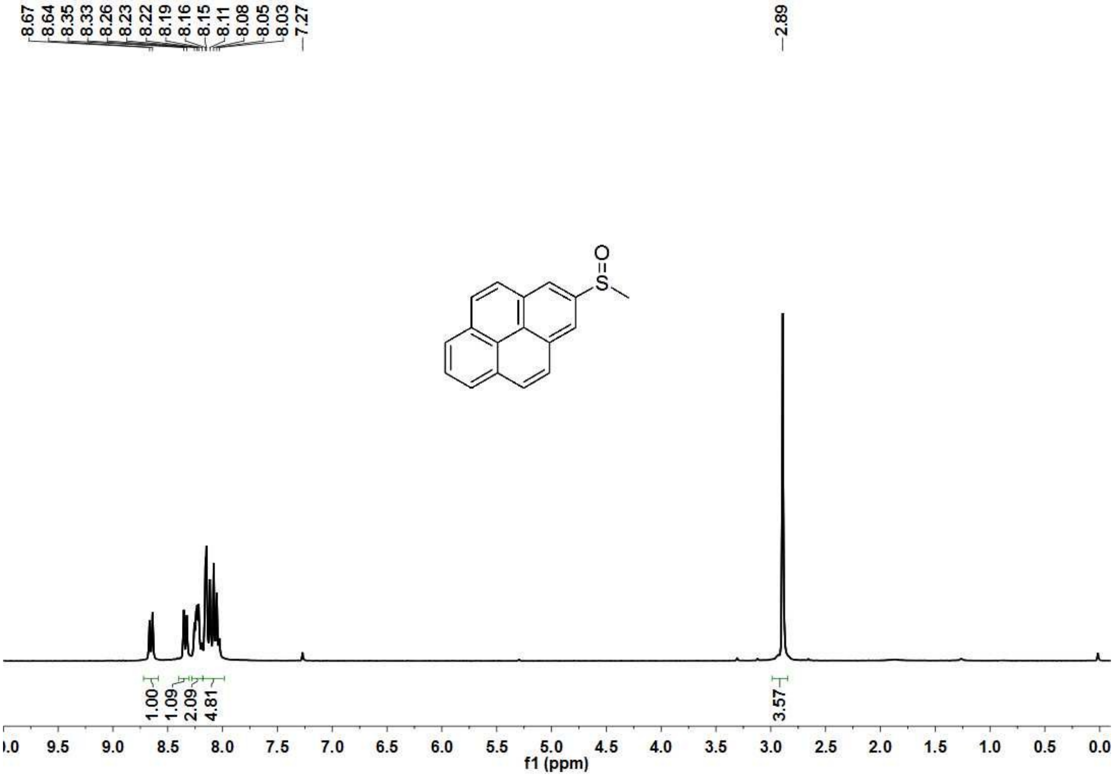

Figure S39. ^{1}H NMR spectra of compoud **13**.

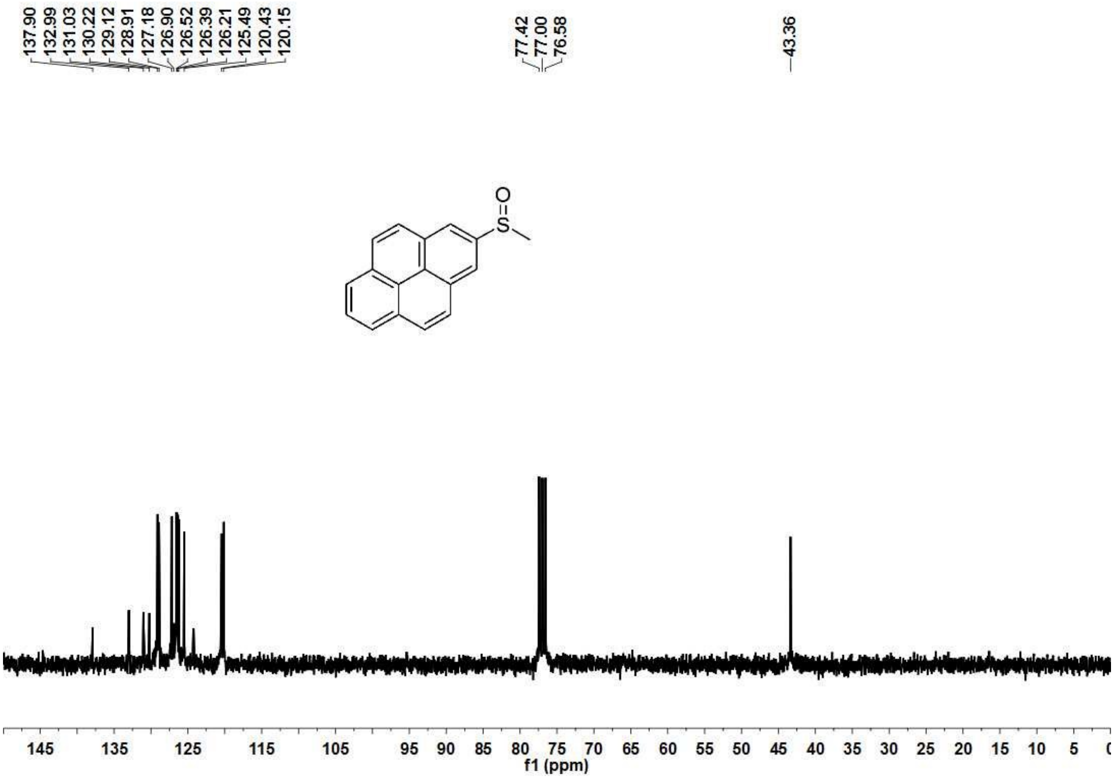

Figure S40. ^{13}C NMR spectra of compoud **13**.

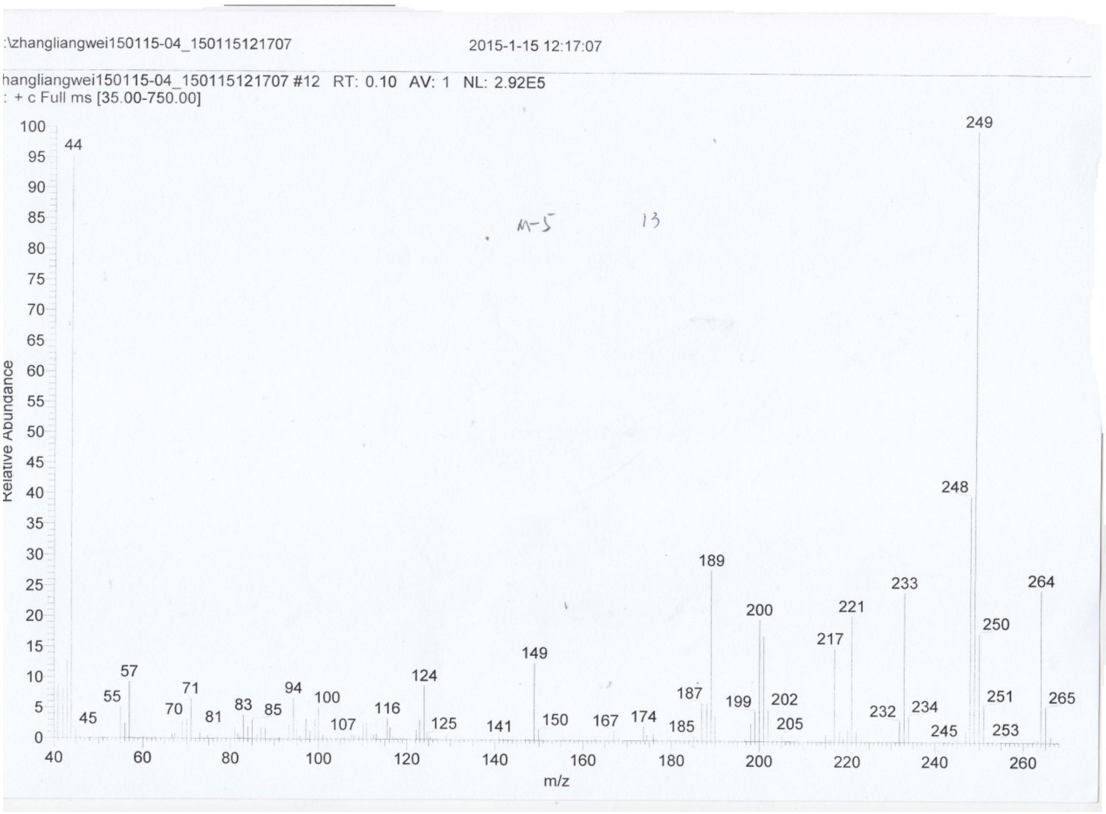

Figure S41. MS spectra of compound **13**.

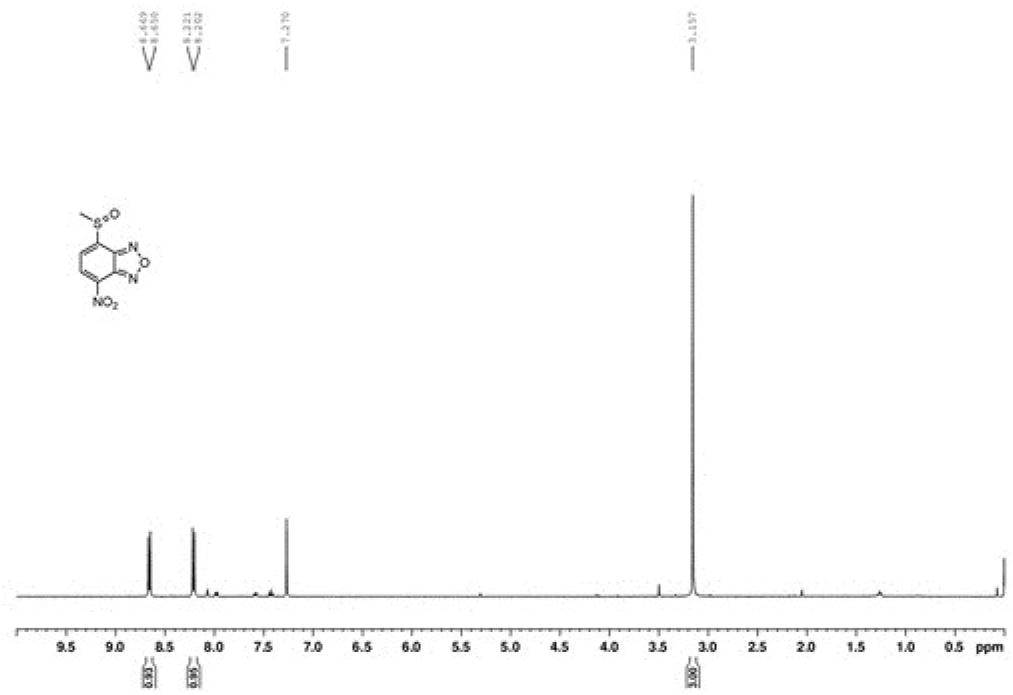

Figure S42. ^{1}H NMR spectra of compoud **14**

.

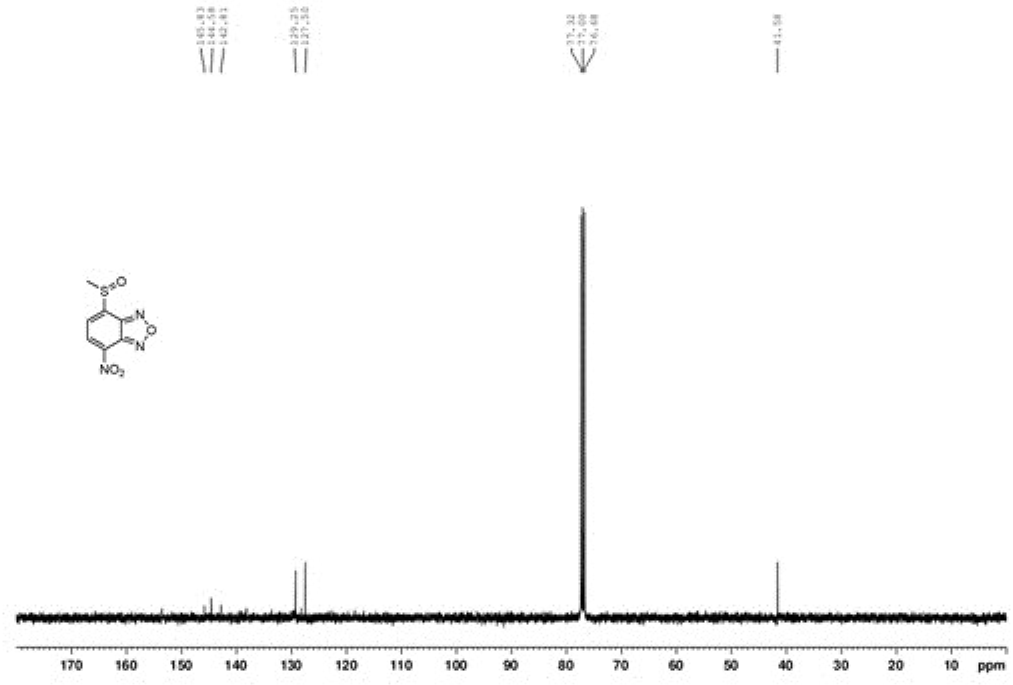

Figure S43. ^{13}C NMR spectra of compoud **14**.

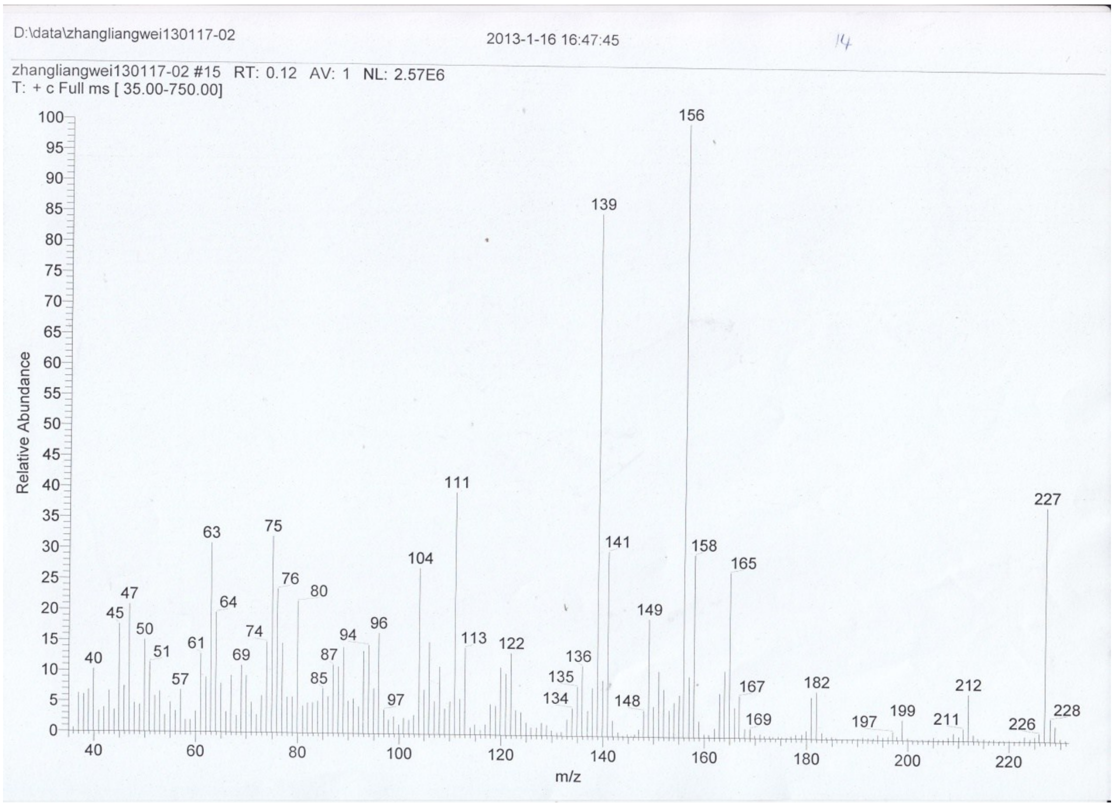

Figure S44. MS spectra of compound **14**.

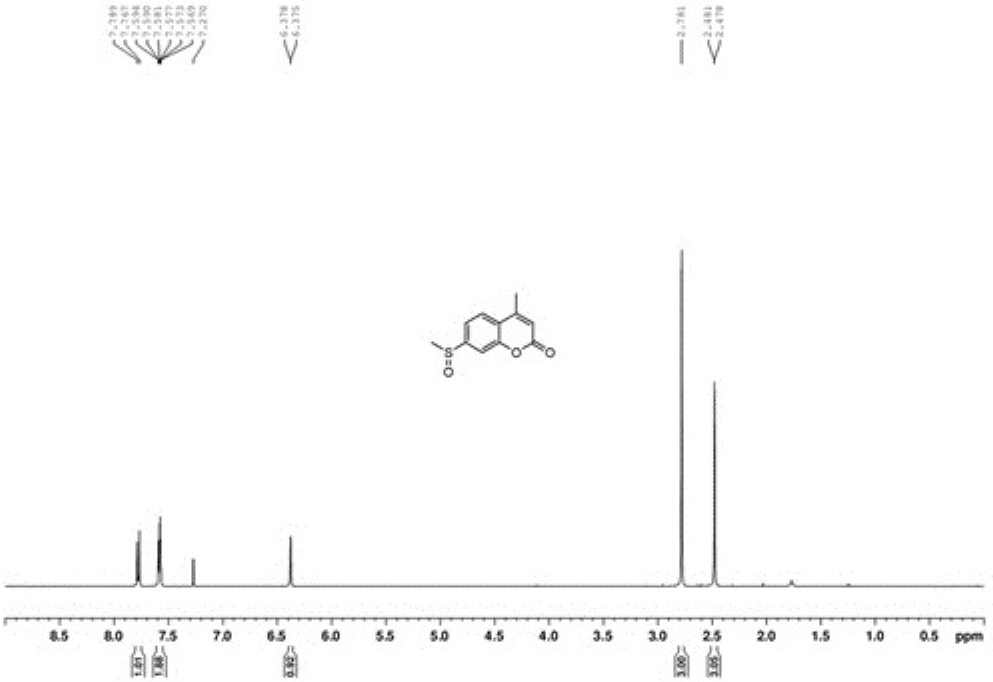

Figure S45. ^{1}H NMR spectra of compoud **15**.

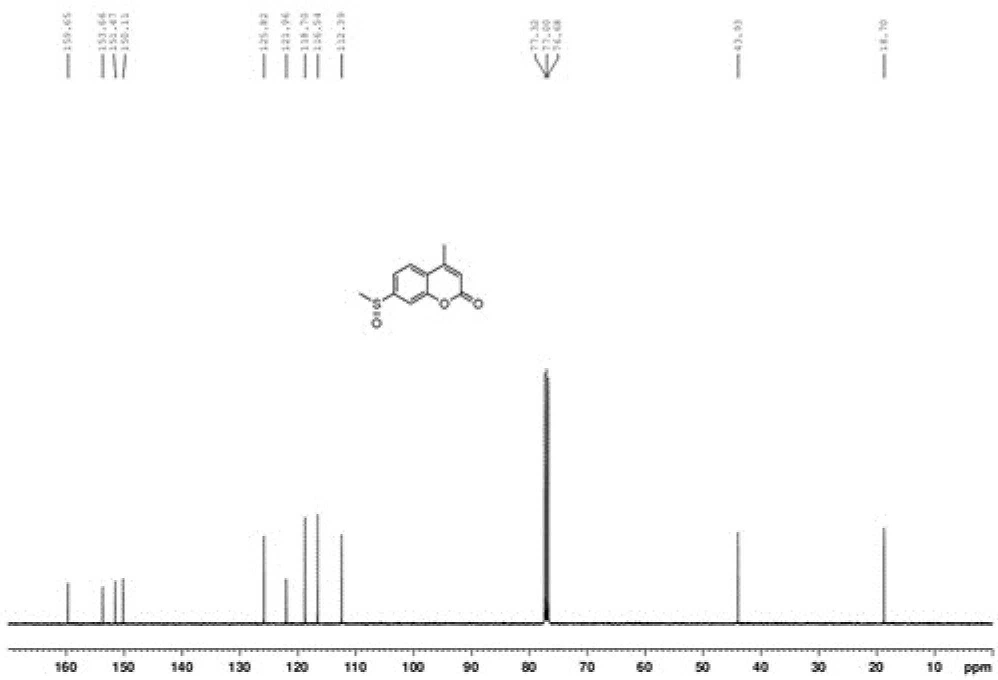

Figure S46. ^{13}C NMR spectra of compoud **15**.

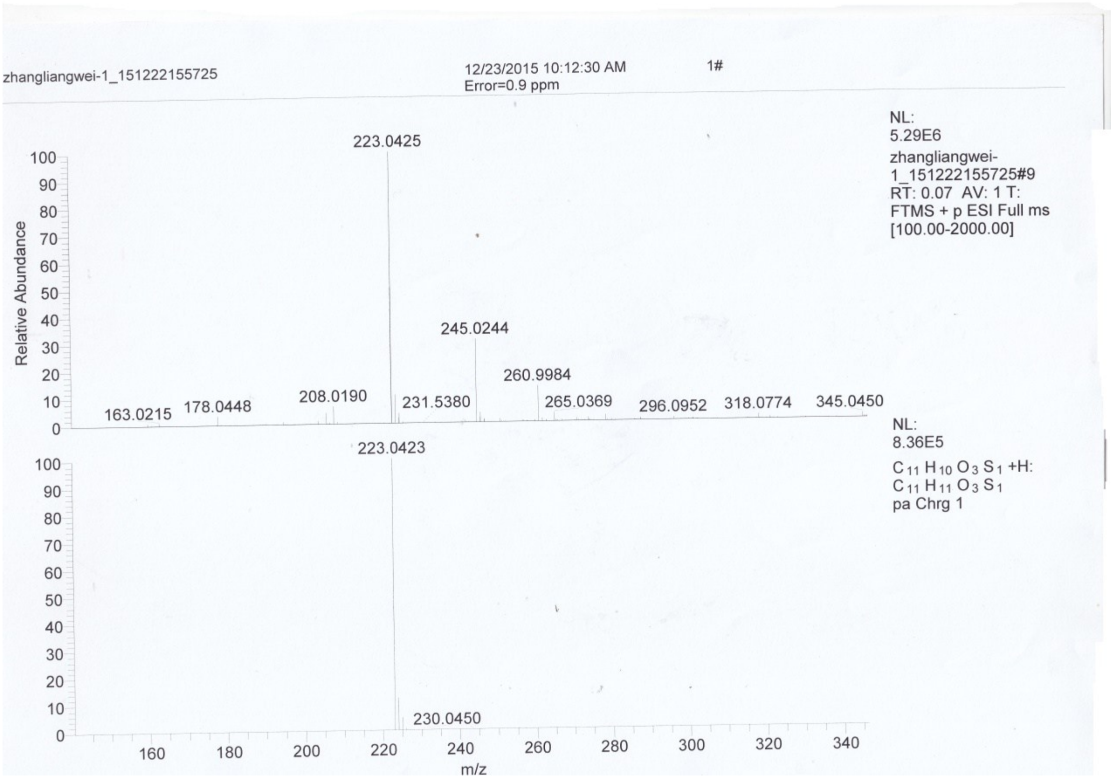

Figure S47. MS spectra of compound **15**.

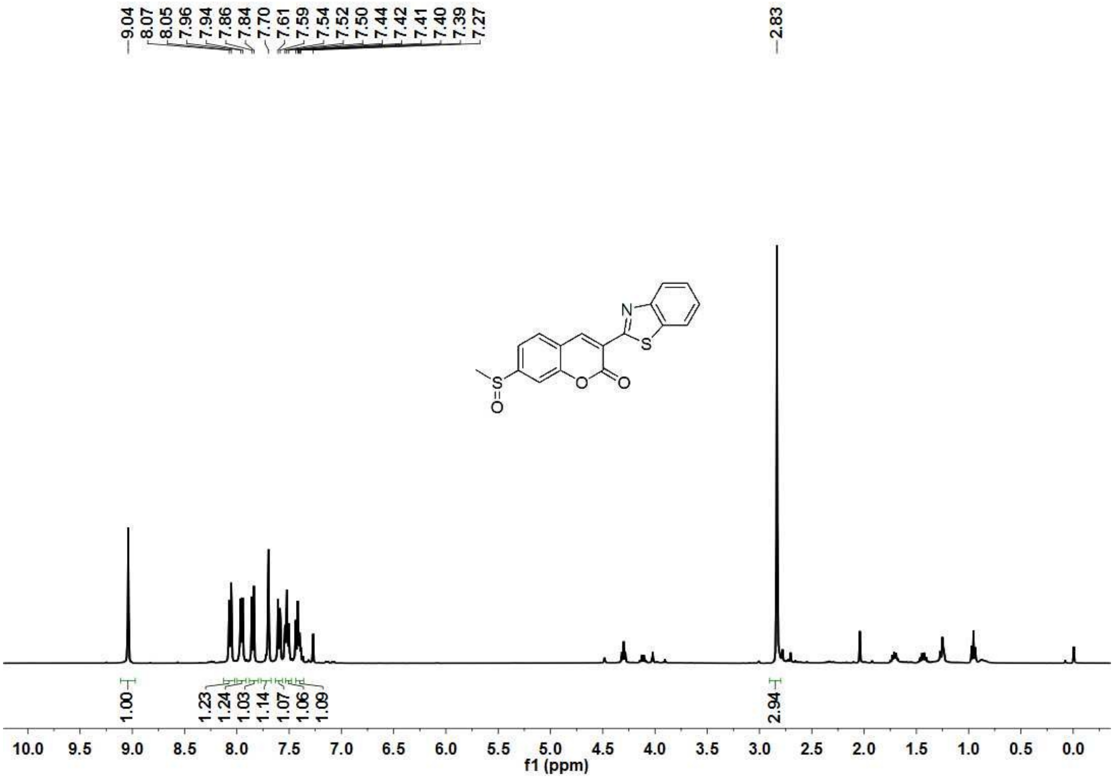

Figure S48. ^{1}H NMR spectra of compoud **16**.

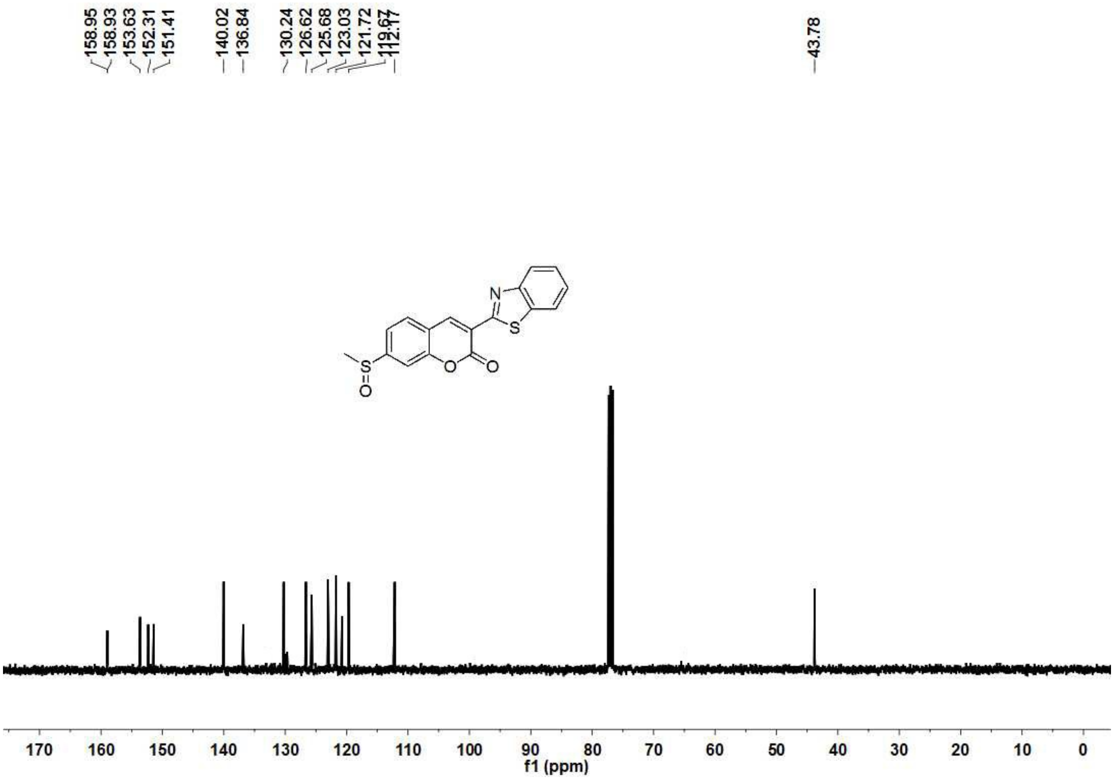

Figure S49. ^{13}C NMR spectra of compoud 1**6**.

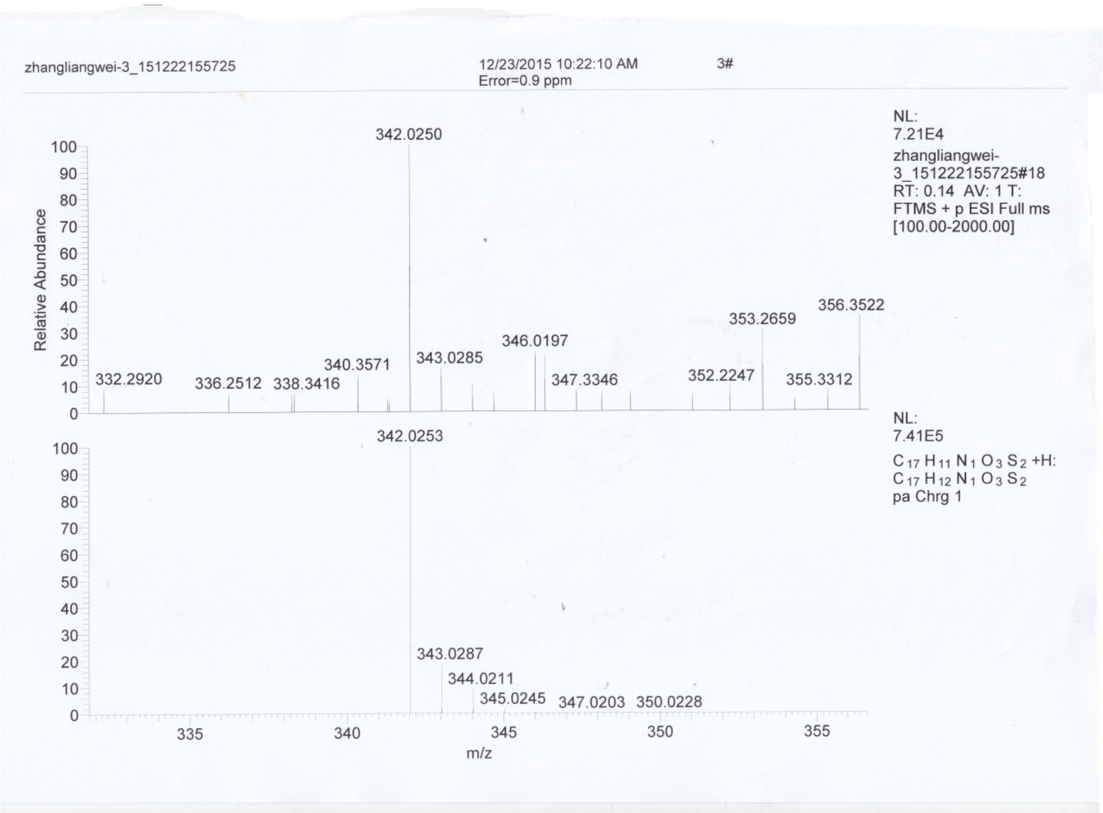

Figure S50. MS spectra of compound **16**.

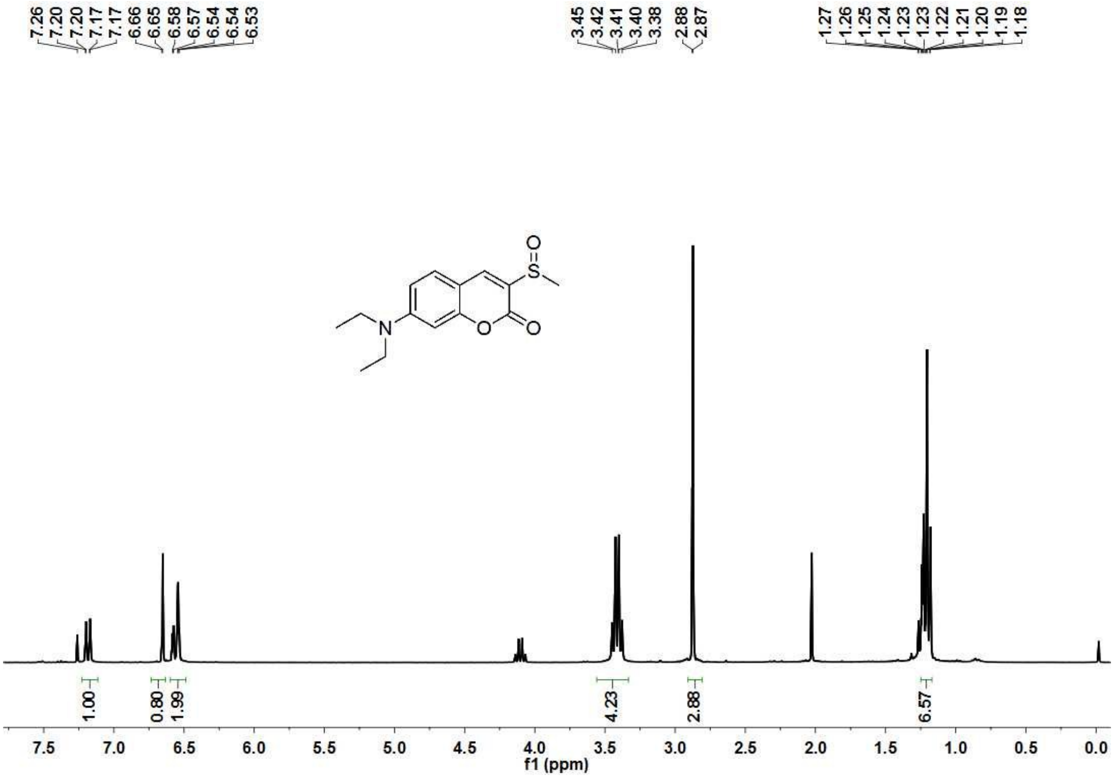

Figure S51. ^{1}H NMR spectra of compoud **17**.

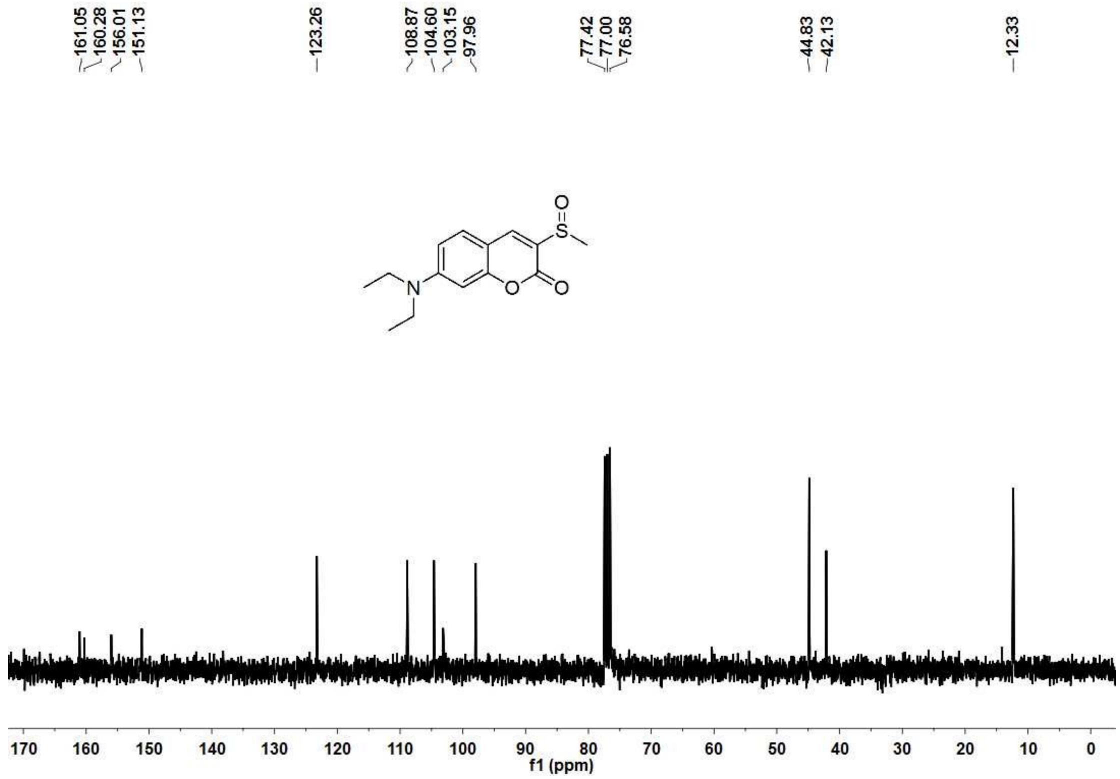

Figure S52. ^{13}C NMR spectra of compoud **17**.

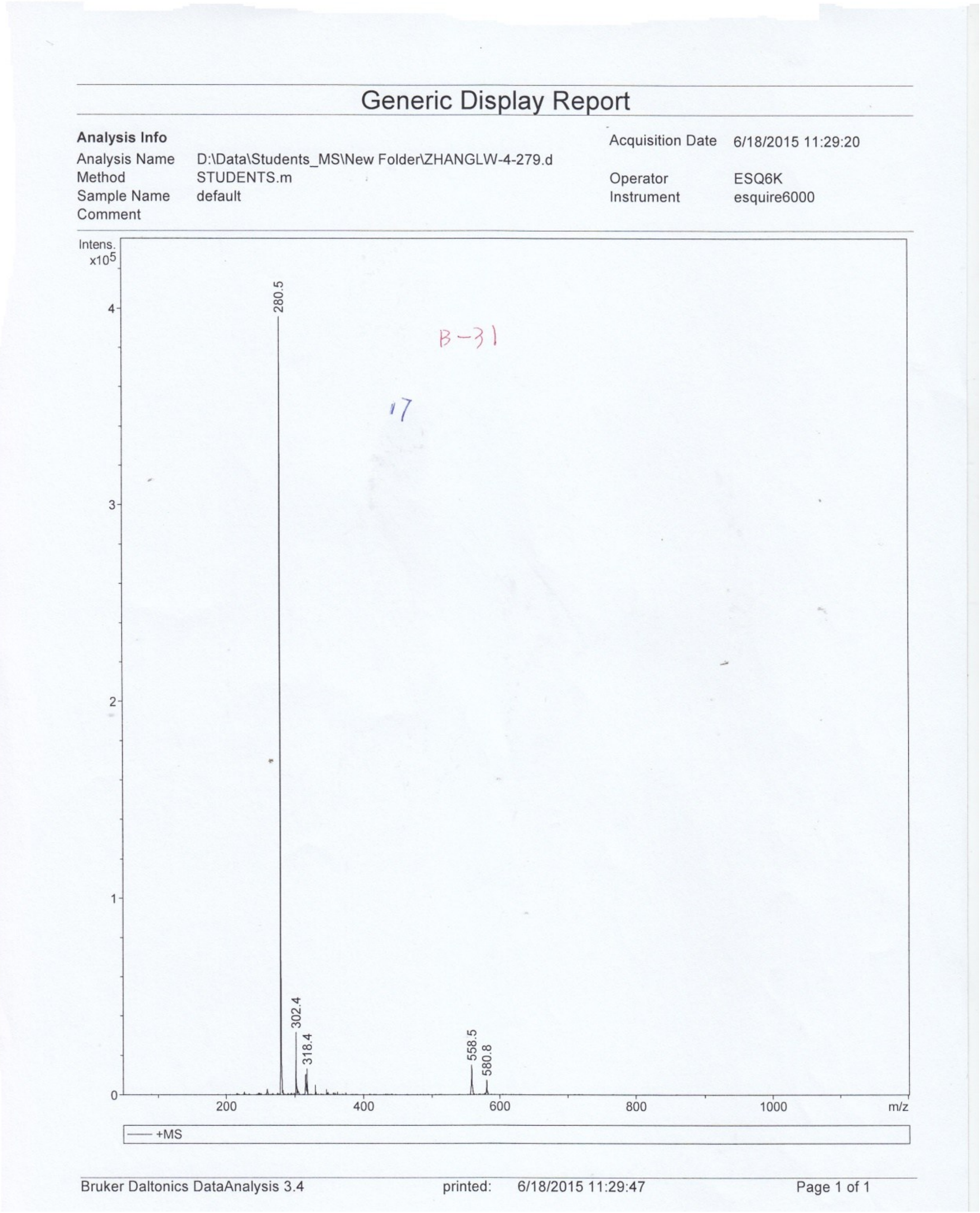

Figure S53. MS spectra of compound **17**.

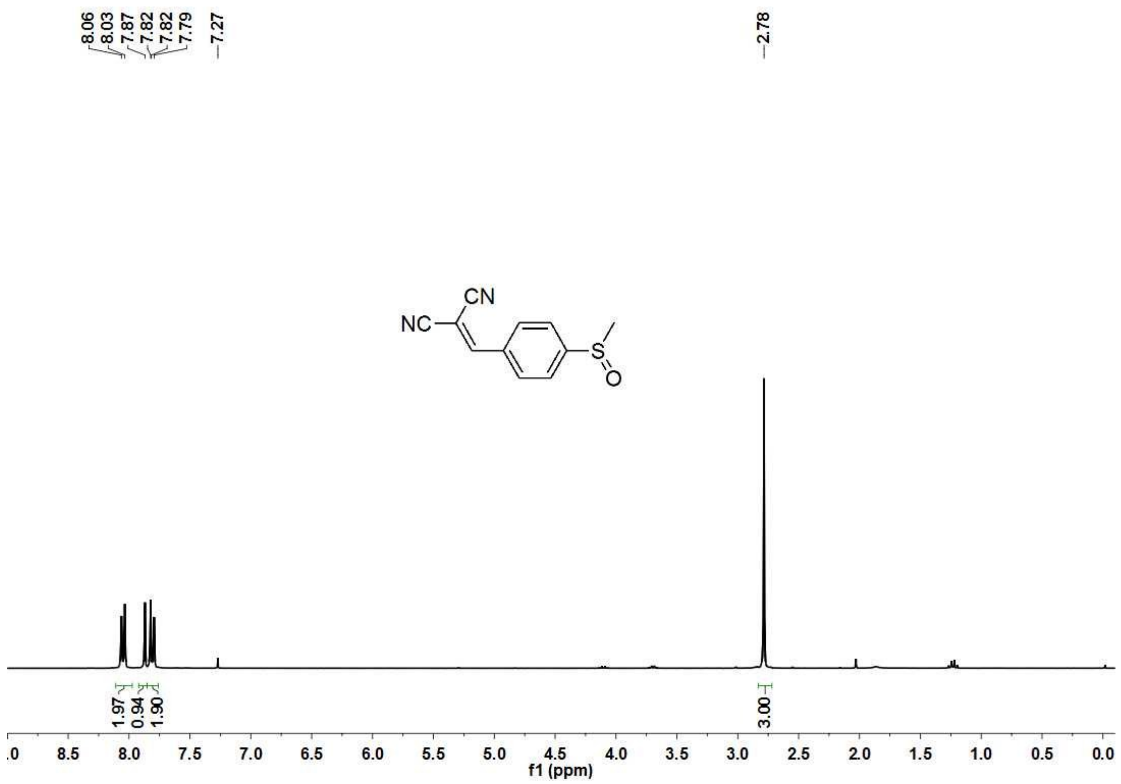

Figure S54. ^{1}H NMR spectra of compoud **18**.

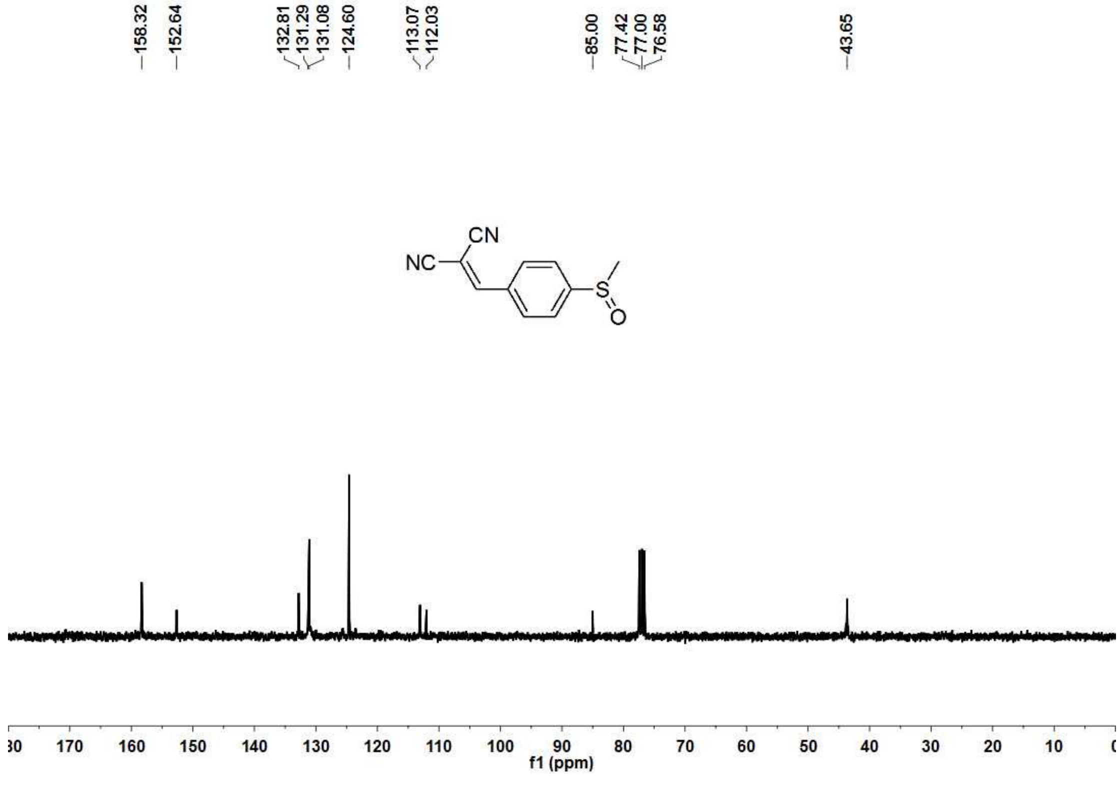

Figure S55. ^{13}C NMR spectra of compoud **18**.

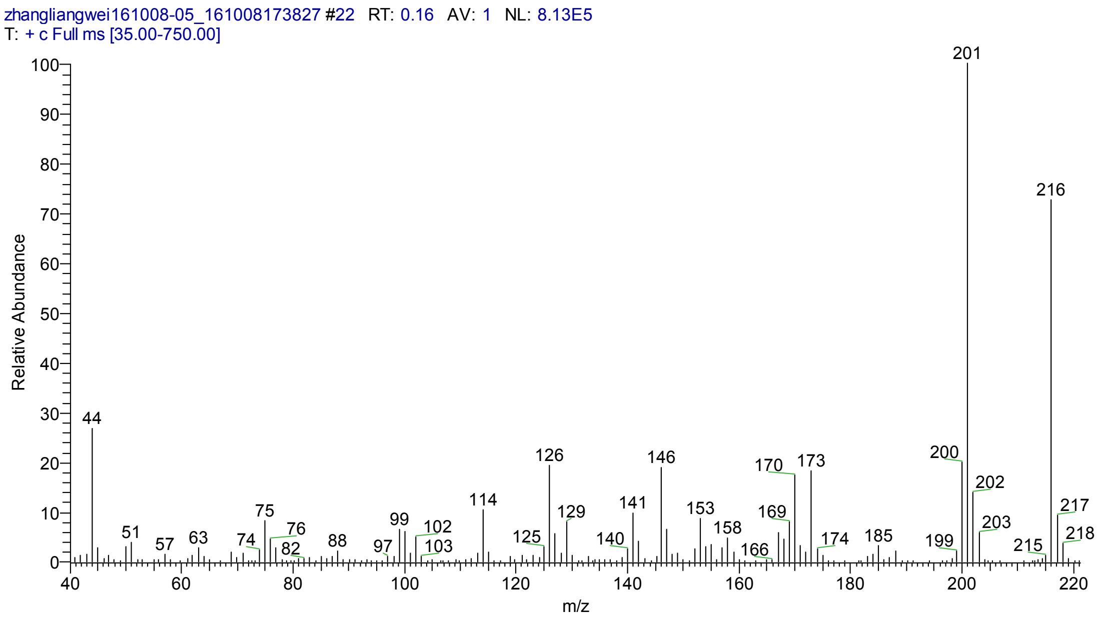

Figure S56. MS spectra of compound **18**.

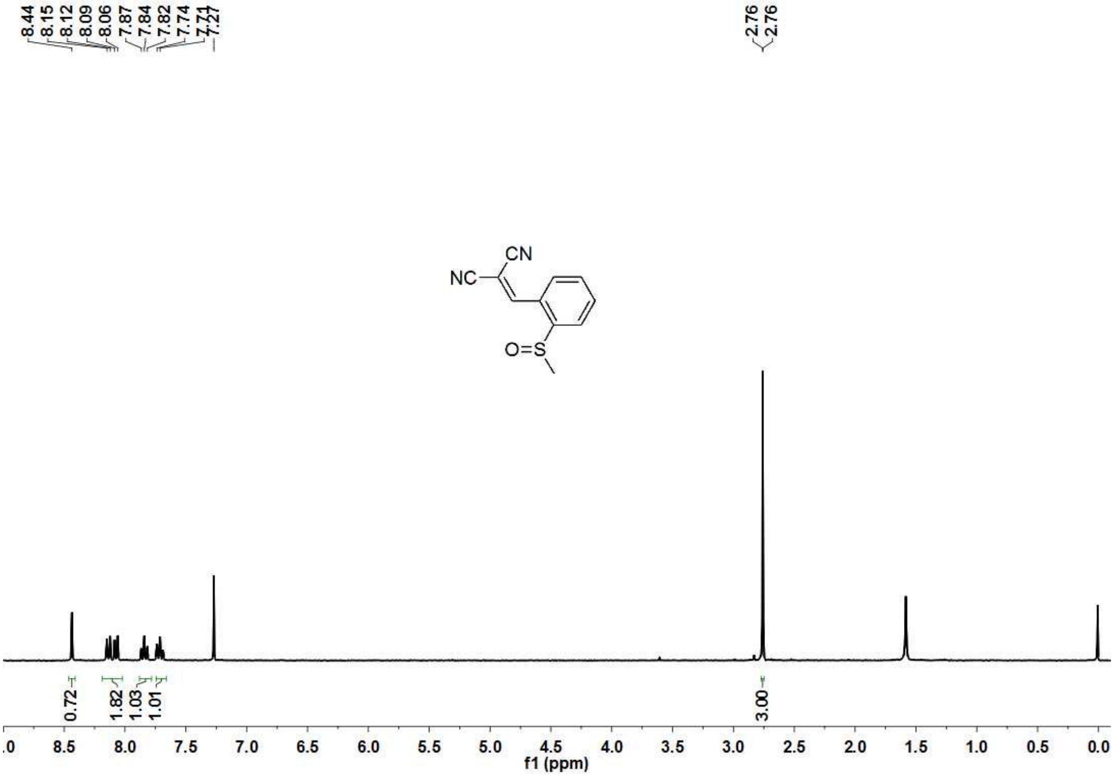

Figure S57. ^{1}H NMR spectra of compoud **19**.

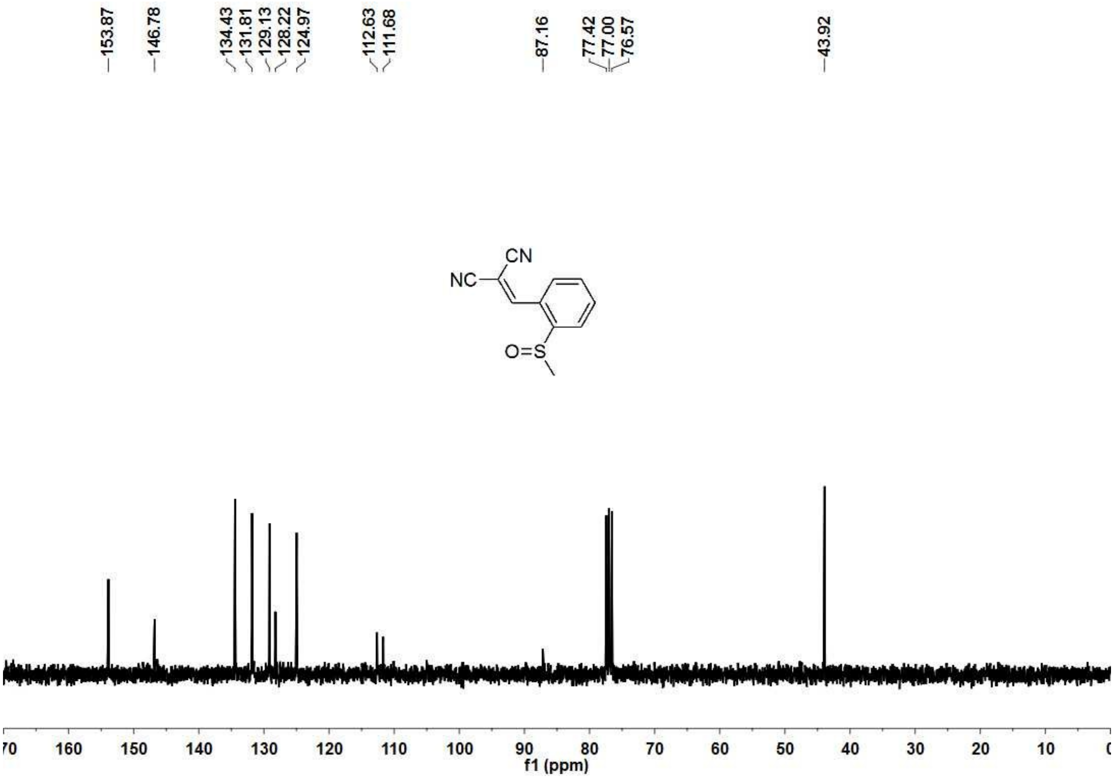

Figure S58. ^{13}C NMR spectra of compoud **19**.

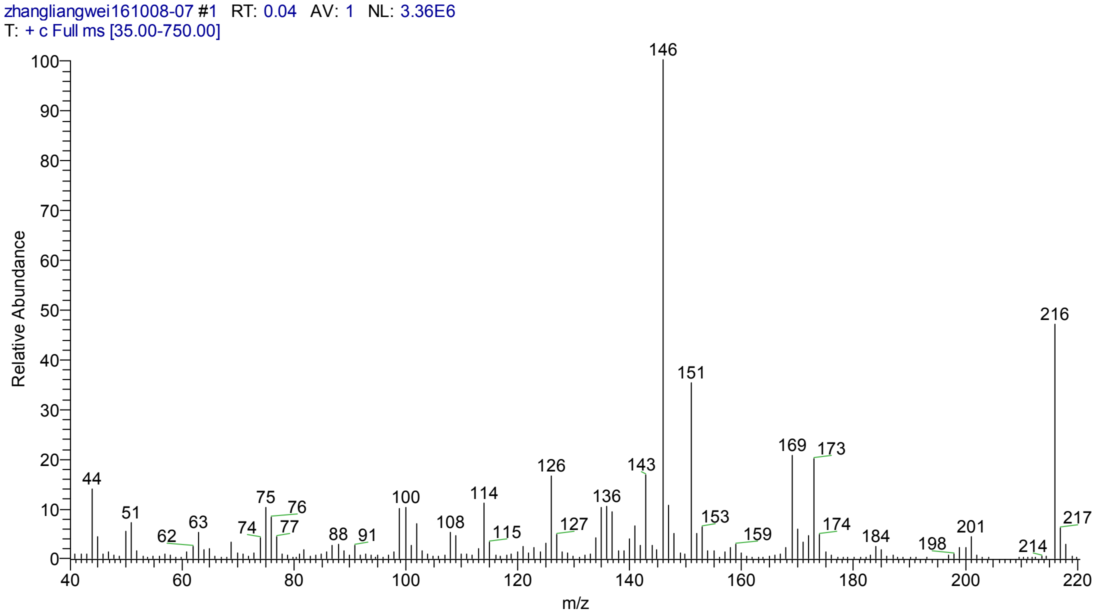

Figure S59. MS spectra of compound **19**.

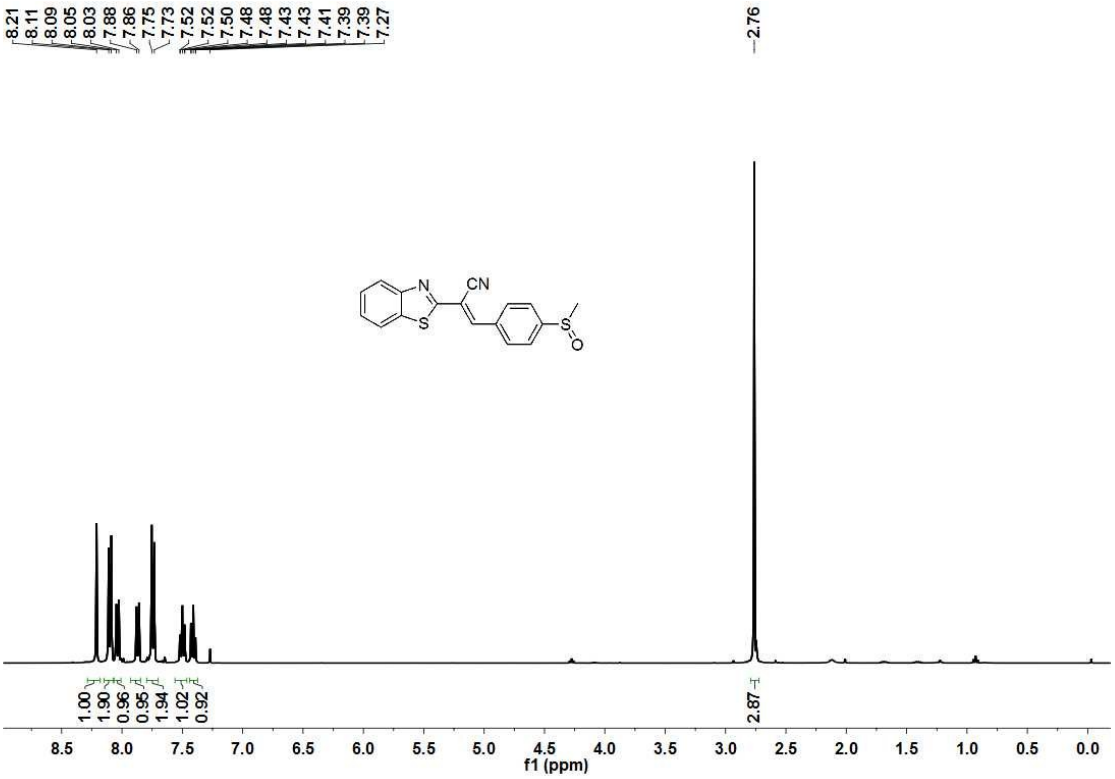

Figure S60. ^{1}H NMR spectra of compoud **20**.

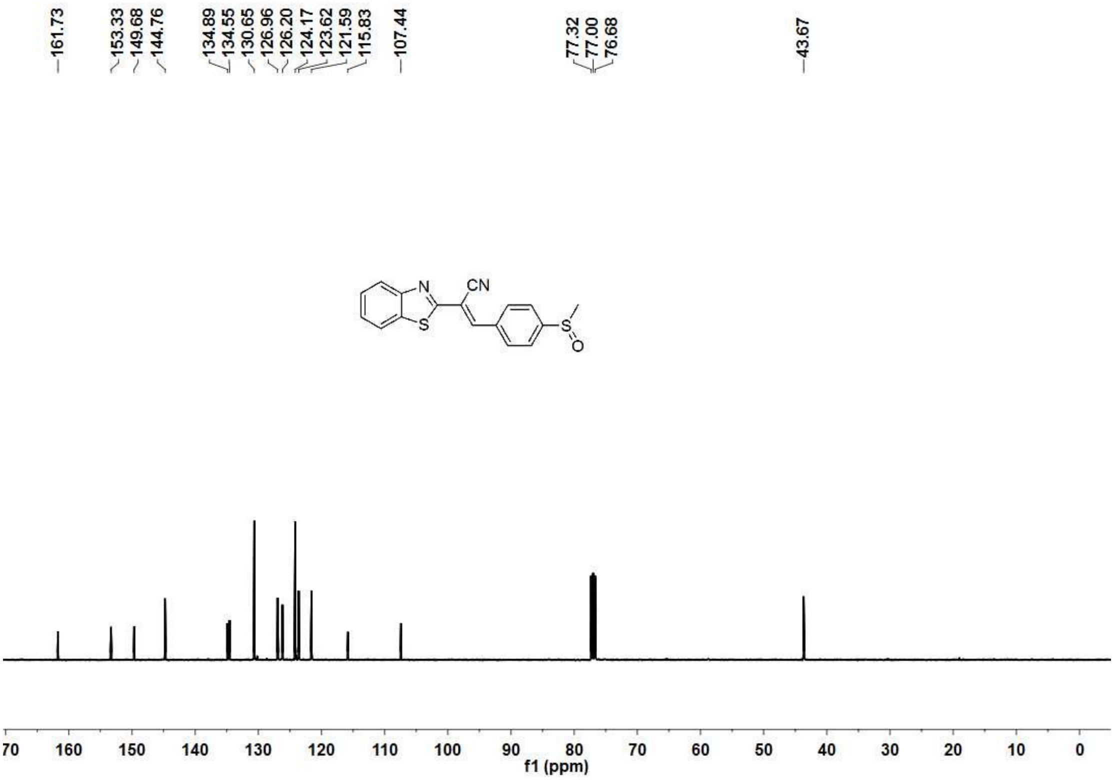

Figure S61. ^{13}C NMR spectra of compoud **20**.

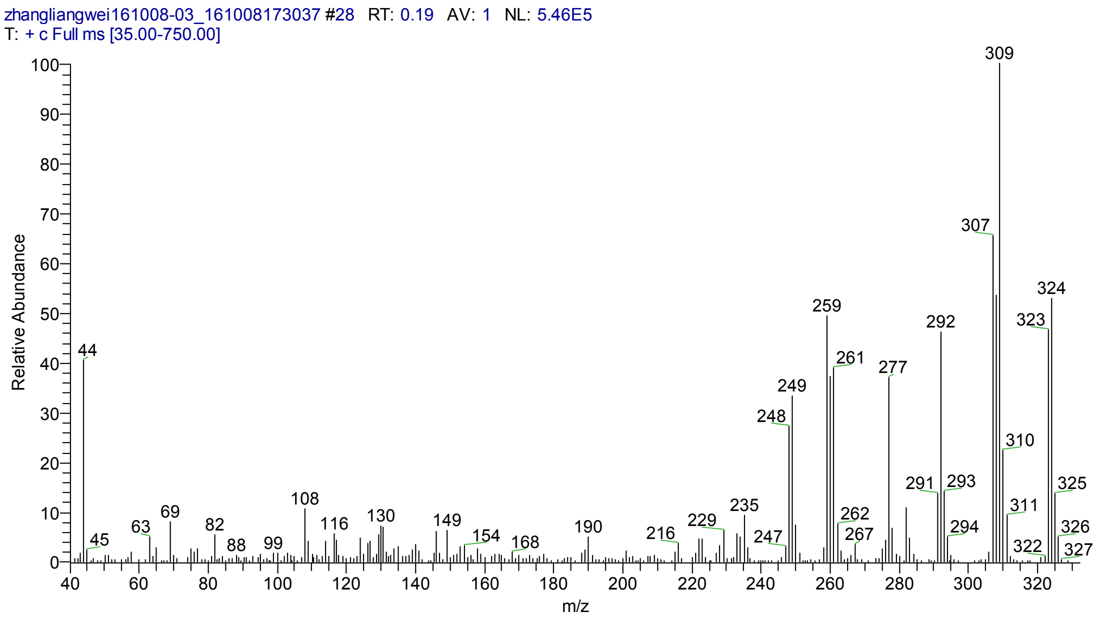

Figure S62. MS spectra of compound **20**.

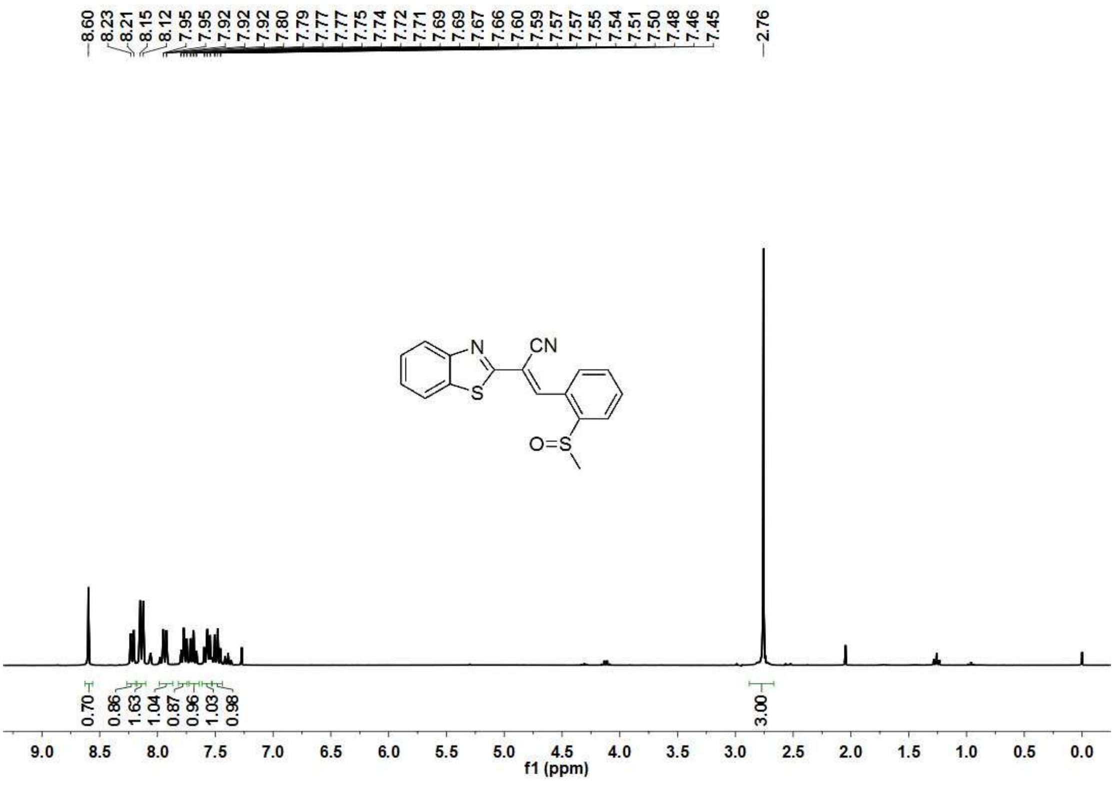

Figure S63. ^{1}H NMR spectra of compoud 2**1**.

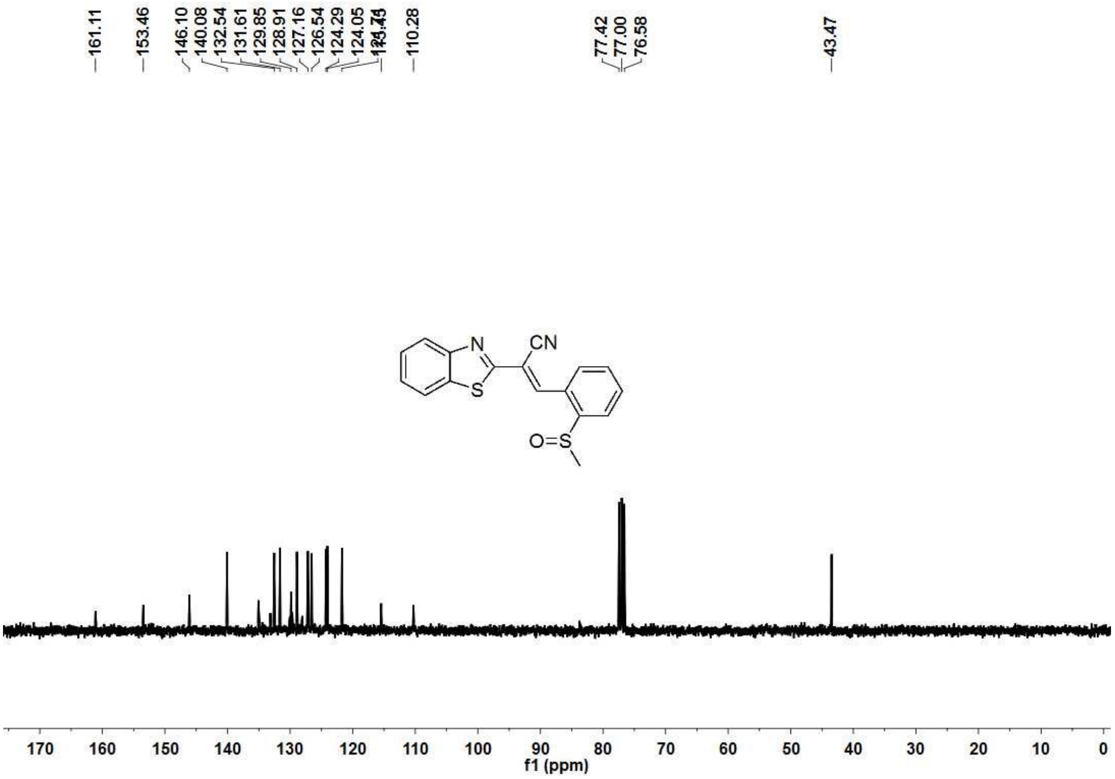

Figure S64. ^{13}C NMR spectra of compoud **21**.

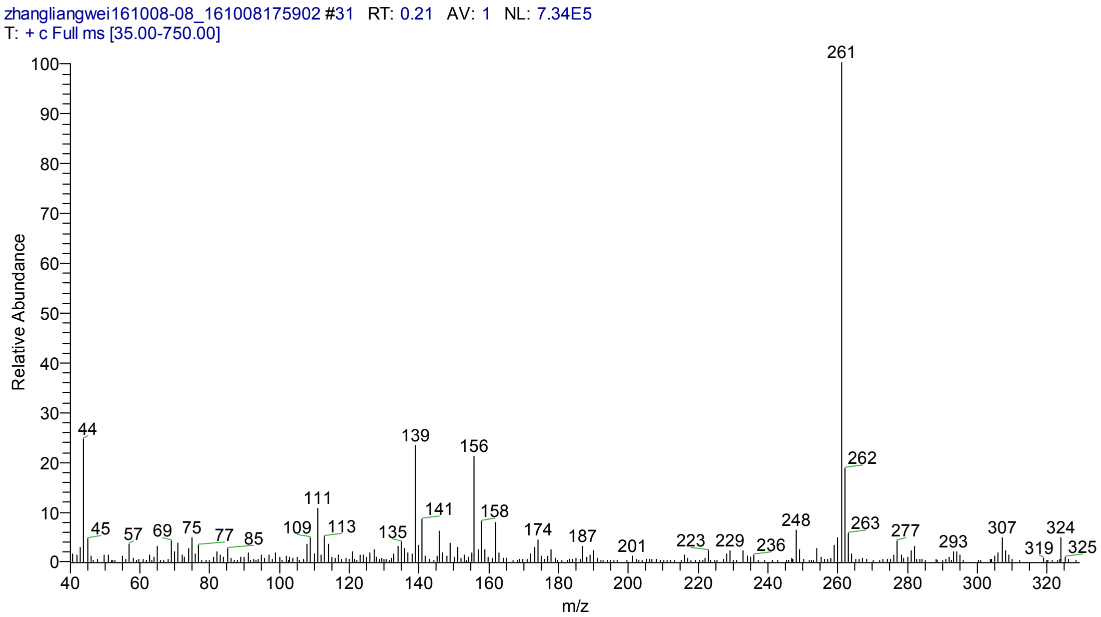

Figure S65. MS spectra of compound **21**.

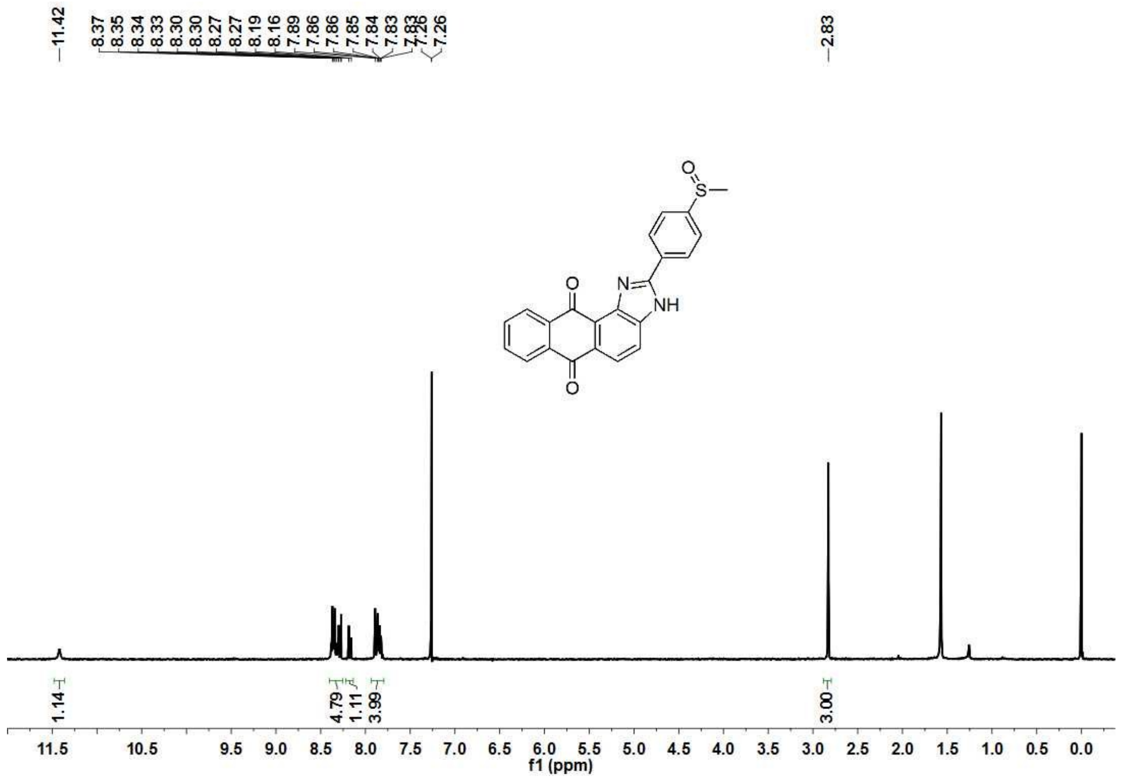

Figure S66. ^{1}H NMR spectra of compoud **22**.

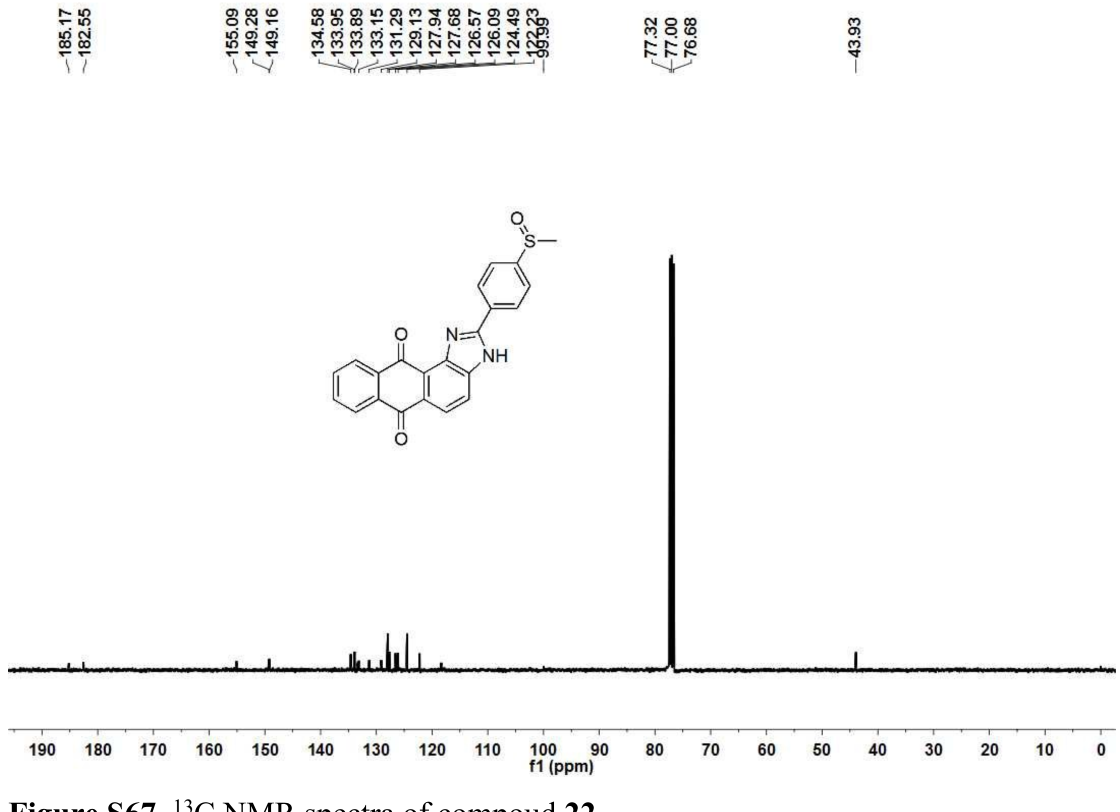

Figure S67. ^{13}C NMR spectra of compoud **22**.

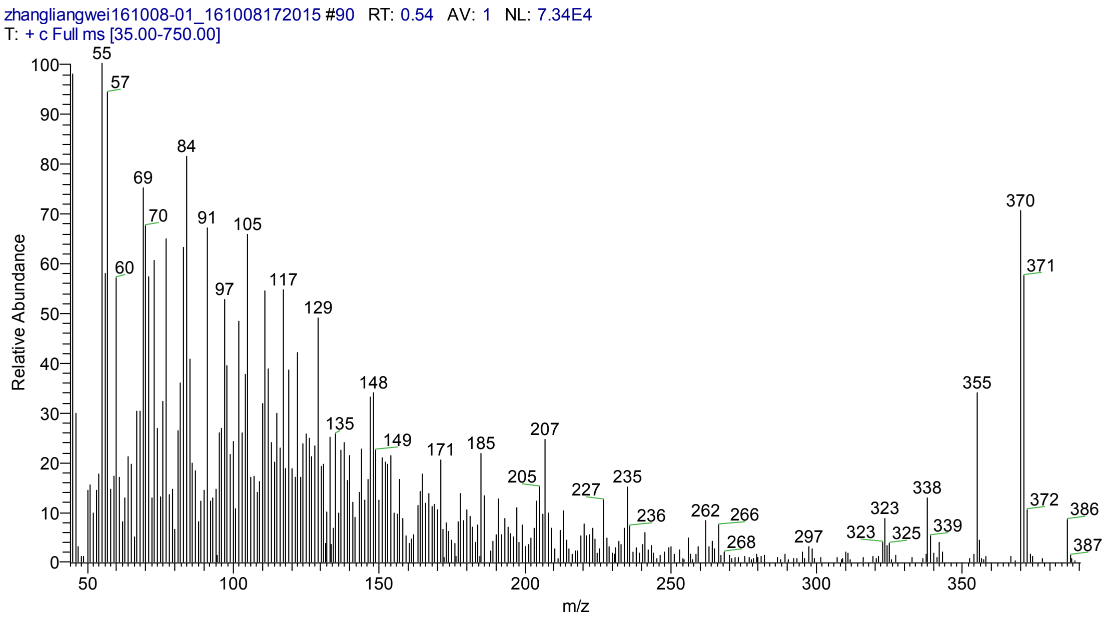

Figure S68. MS spectra of compound **22**.

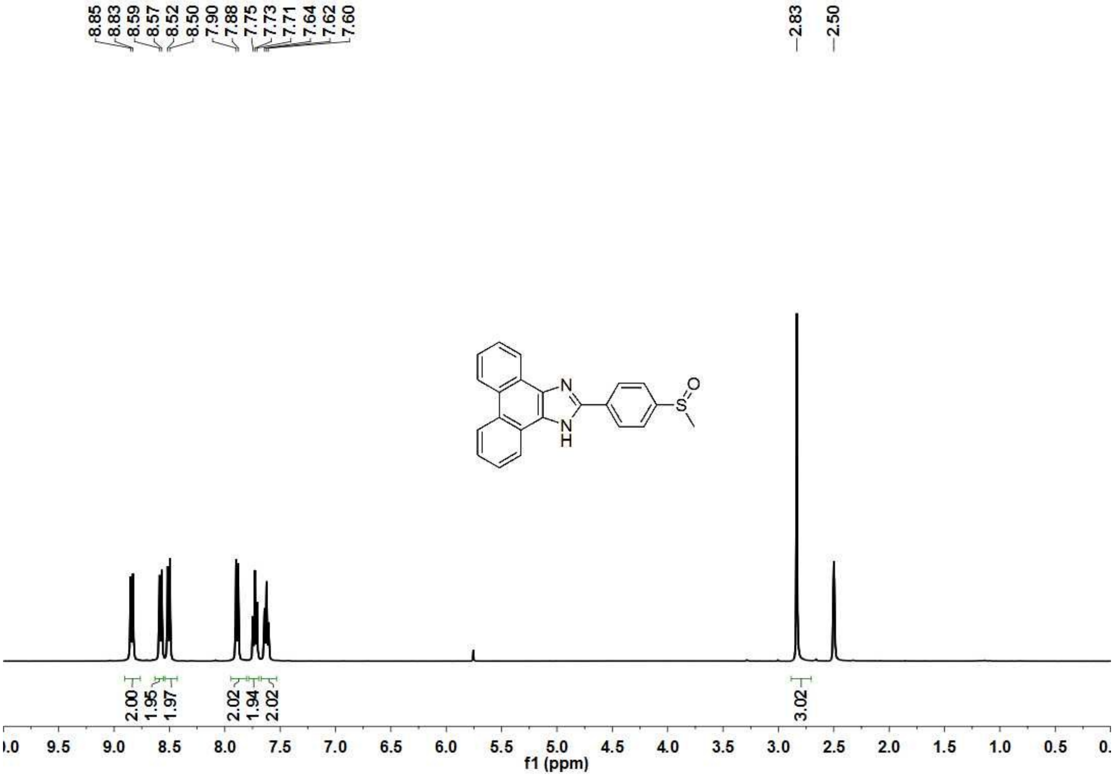

Figure S69. ^{1}H NMR spectra of compoud **23**.

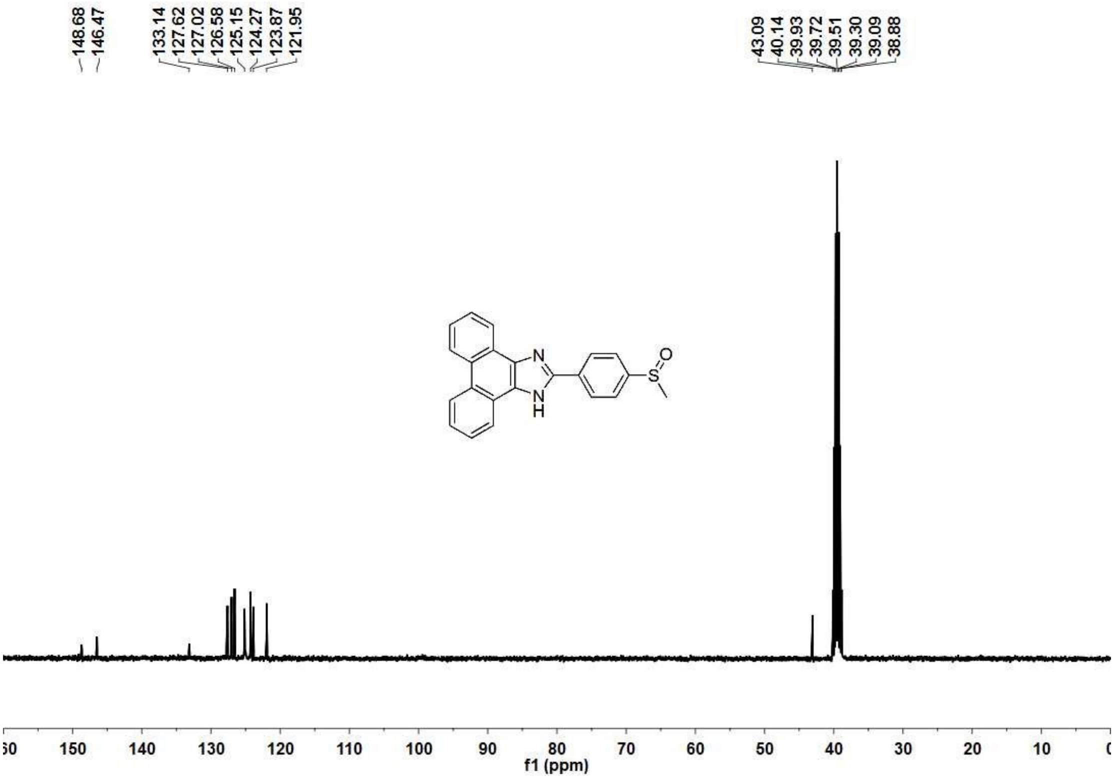

Figure S70. ^{13}C NMR spectra of compoud **23**.

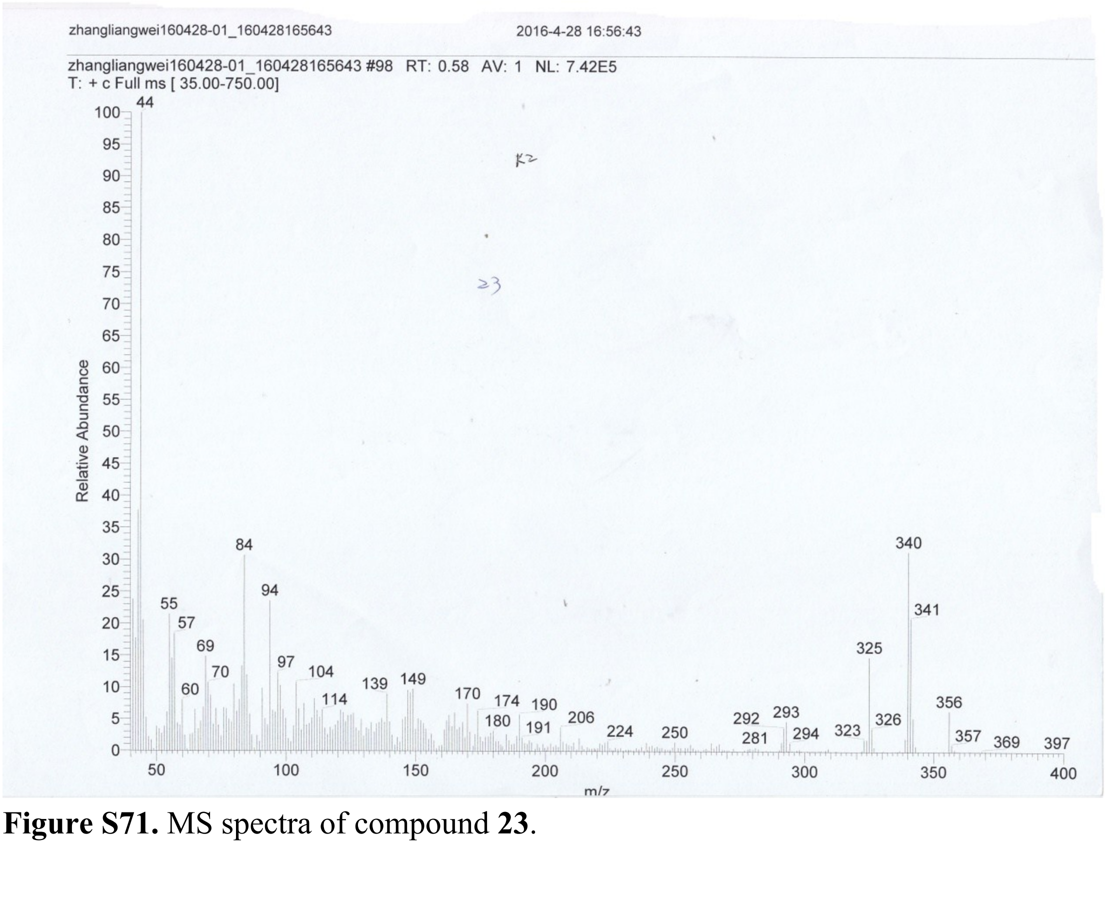

Figure S71. MS spectra of compound **23**.

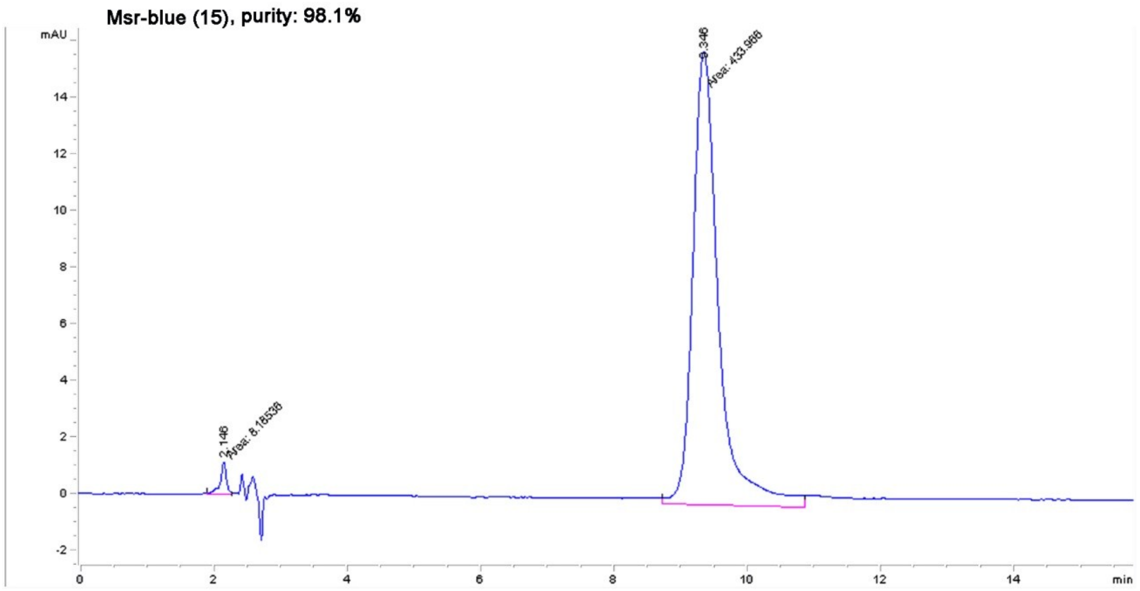

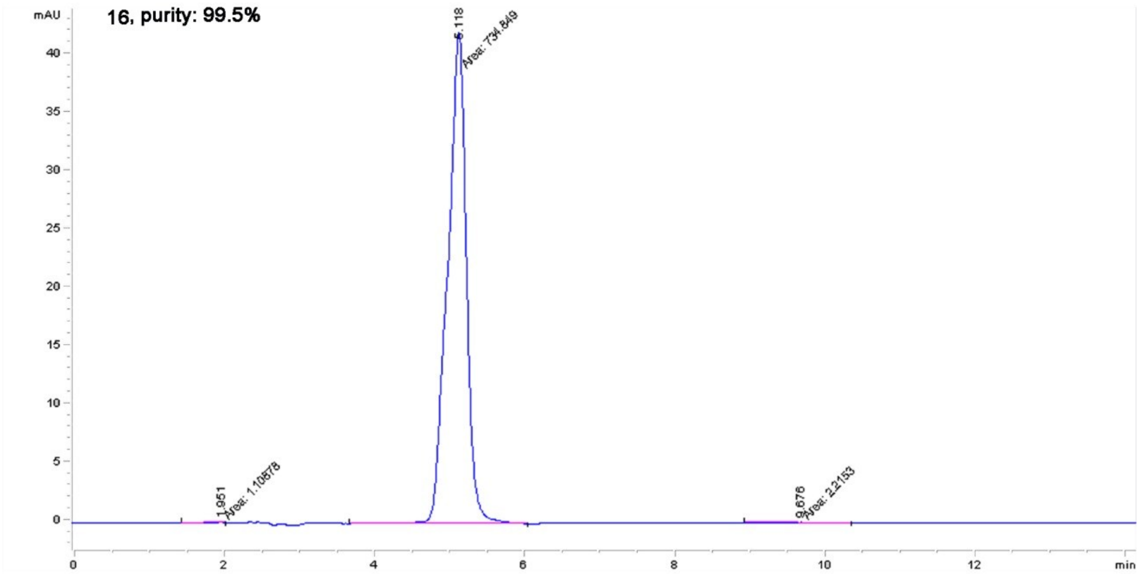

Figure S72. Analyses of the purity of Msr-blue and **16** by HPLC. The chromatograms of the probes were shown, and the purity of Msr-blue and **16** was 98% and 99%, respectively.

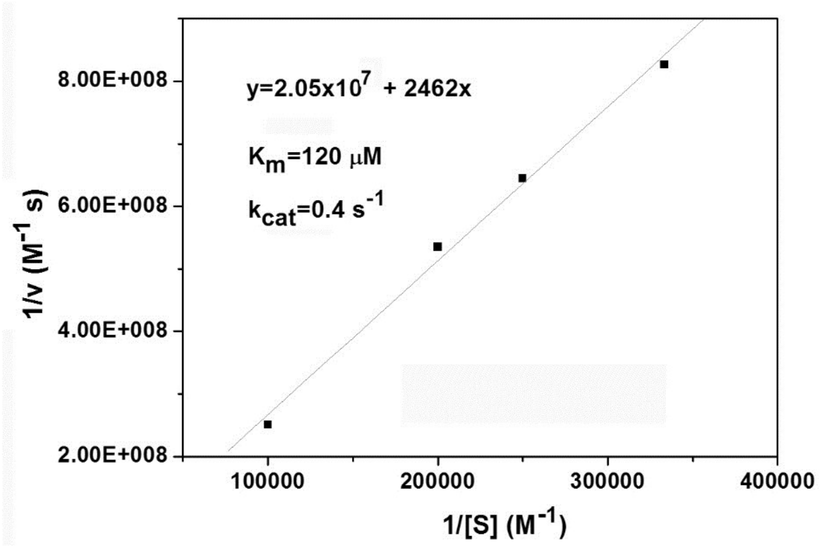

Figure S73. Lineweaver-Burk plot for the enzymatic reduction of Msr-blue catalyzed by Msr A. The K_m was found to be 120 μM and the k_{cat} 0.4 s^{-1}. The concentrations of Msr-blue are ranging from 0-20 μM, and the enzyme concentration is 3 μg/ml (120 nM). The data were calculated by following these assumptions: 1) Only the *S*-diastereoisomer serves as the substrate for Msr A, 2) The *R*-diastereoisomer does not act as either an inhibitor or substrate, and 3) The racemic mixture of Msr-blue contains the equal amount of both diastereoisomers. The K_{cat} value was calculated based on effective concentrations of the *S*-diastereoisomer of Msr-blue, which are the half of the Msr-blue concentrations used in the experiments.

Experimental section

Unless stated otherwise, all reagents were of analytical grade and were purchased from commercial supplies. NMR spectra were recorded on Bruker 400 MHz instruments or Agilent Mercury plus 300 BB. MS spectra were recorded on Trace DSQ GC-MS spectrometer or Bruker Daltonics esquire 6000 mass spectrometer. HRMS was obtained on Orbitrap Elite (Thermo Scientific). Live cell imaging was carried out on a laser scanning confocal microscope (Olympus FV1000). Fluorescence studies were carried out using an Agilent Cary Eclipse Fluorescence Spectrophotometer (the silt wide was 5 nm for both excitation and emission). The recombinant Msr A was kindly provided by Prof. Hong Yu from the School of Basic Medical Sciences, Wuhan University, China.[1]

Chemical Synthesis.

Synthesis of 1b. Compound **1a** was synthesized according to the reported work in two steps.[2] Compound **1a** (277 mg, 1mmol) was dissolved in dimethylformamide (DMF, 20 mL), and the solution of CH_3SNa (1.05 g, 20% aqueous solution, 0.2 mmol) was added slowly to the mixture. After the mixture reacted for 2h at room temperature, it was poured into the water and the precipitate was filtered and washed with water twice. The crude product was dried in vacuum oven without further purification (224 mg, 92%). ^{1}H NMR (300 MHz, DMSO) δ 8.48 (ddd, *J*=7.5, 5.9, 0.9 Hz, 2H), 8.32 (d, *J*=8.0 Hz, 1H), 7.86 (dd, *J*=8.4, 7.4 Hz, 1H), 7.61 (d, *J*=8.0 Hz, 1H), 2.73 (s, 3H). ^{13}C NMR (75 MHz, DMSO) δ 160.67, 160.45, 147.80, 132.70, 132.17, 130.26, 129.40, 127.97, 127.43, 121.50, 119.45, 114.29, 14.03. EI-MS (m/z, %): 244 (M^+, 100), 44 (56).

Synthesis of 1. Compound **1b** (244 mg, 1mmol) was dissolved in dichloromethane (DCM, 20 mL), and meta-chloroperoxybenzoic acid (*m*-CPBA, 172 mg, 1mmol) was added successively to the solution. The mixture was stirred for another 15 min at room temperature. Upon solvent evaporation, the crude product was purified by silica gel column chromatography (petroleum ether:AcOEt from 3:1 to 0:1 (v/v), 189 mg, 73% yield). ^{1}H NMR (300 MHz, DMSO) δ 8.69 (d, *J*=7.6 Hz, 1H), 8.56 (dd, *J*=20.8, 7.9 Hz, 2H), 8.34 (d, *J*=7.6 Hz, 1H), 8.00 (t, *J*=7.9 Hz, 1H), 2.92 (s, 3H). ^{13}C NMR (75 MHz, DMSO) δ 160.28, 151.04, 132.79, 131.92, 129.68, 129.29, 128.78, 126.53, 123.01, 121.45, 120.12, 42.93. EI-MS (m/z, %): 260 (M^+, 100), 245 (54), 214 (54), 201 (73).

Synthesis of 2'.[3] Compound **1b** (2.44 g, 10mmol) was dissolved in 20 mL EtOH, and *n*-butylamine (2.94 mL, 40 mmol) was slowly added to the stirred solution. After refluxing for 8h, the solvent was removed in vacuum to yield the crude product and purified by silica gel column chromatography (petroleum ether:AcOEt 3:1 (v/v), 1.85 g, 62%). ^{1}H NMR (400 MHz, $CDCl_3$) δ 8.55 (d, *J*=7.3 Hz, 1H), 8.41 (d, *J*=8.1 Hz, 2H), 7.68 (t, *J*=7.9 Hz, 1H), 7.37 (d, *J*=7.9 Hz, 1H), 4.15 (t, 2H), 2.67 (s, 3H), 1.71 (ddd, *J*=12.8, 8.6, 6.6 Hz, 2H), 1.45 (dq, *J*=14.7, 7.4 Hz, 2H), 0.98 (t, *J*=7.4 Hz, 3H). ^{13}C NMR (100 MHz, $CDCl_3$) δ 163.94, 163.91, 146.11, 131.27, 130.68, 129.42, 128.79, 127.94, 126.39, 123.06, 120.84, 118.71, 40.14, 30.18, 20.35, 14.78, 13.81. ESI-MS (m/z): $[M+H]^+$ 300.2.

Synthesis of 2. Compound **2'** (299 mg, 1mmol) was dissolved in tetrahydrofuran (THF, 50 mL) and kept on ice, and NaClO (0.74g, 1mmol, 10%) were added slowly to the solution. The reaction was monitored by TLC. Subsequently, the solvent was removed and the crude product was purified by silica gel column chromatography to yield the white solid. (280 mg, 89%). ^{1}H NMR (400 MHz,

$CDCl_3$) δ 8.72 (d, *J*=8 Hz, 1H), 8.63 (dd, *J*=7.3, 0.9 Hz, 1H), 8.39 (d, *J*=7.6 Hz, 1H), 8.25 (dd, *J*=8.4, 0.9 Hz, 1H), 7.84 (dd, *J*=8.4, 7.4 Hz, 1H), 4.14 (t, 2H), 2.87 (s, 3H), 1.73-1.64 (m, 2H), 1.42 (dq, *J*=14.7, 7.4 Hz, 2H), 0.95 (t, *J*=7.4 Hz, 3H). ^{13}C NMR (100 MHz, $CDCl_3$) δ 163.32, 163.14, 148.89, 131.46, 130.68, 128.09, 128.04, 127.02, 126.90, 124.87, 123.63, 122.83, 43.26, 40.34, 30.03, 20.23, 13.70. ESI-MS (m/z): $[M+H]^+$ 316.3.

Synthesis of 3a.[4] Compound **1a** (277 mg, 1 mmol) was dissolved in acetic acid (AcOH, 20 mL), and phenylamine (930 mg, 10 mmol) was slowly added to the stirred solution. After refluxing for 12 h, the mixture was cooled to room temperature and poured into water, the desired product was obtained by filtration with 51% yield. ^1H NMR (300 MHz, $CDCl_3$) δ 8.69 (d, *J*=7.2 Hz, 1H), 8.63 (d, *J*=8.5 Hz, 1H), 8.45 (d, *J*=7.9 Hz, 1H), 8.07 (d, *J*=7.9 Hz, 1H), 7.94-7.81 (m, 1H), 7.60–7.42 (m, 3H), 7.37-7.27 (m, 2H). ^{13}C NMR (75 MHz, $CDCl_3$) δ 163.76, 135.06, 133.61, 132.42, 131.57, 131.19, 130.77, 130.65, 129.41, 128.83, 128.52, 128.17, 123.21, 122.33. EI-MS (m/z, %): 352 (M^+, 25), 238 (54), 44(100).

Synthesis of 3'. Compound **3a** (350 mg, 1mmol) was dissolved in DMF (20 mL), and the solution of CH_3SNa (1.05 g, 0.2 mmol) was added slowly to the mixture. After the mixture was stirred overnight at room temperature and then poured into water, the crude product was obtained by filtration (89%). ^1H NMR (300 MHz, DMSO) δ 8.67 (d, *J*=7.2 Hz, 1H), 8.56 (dd, *J*=12.5, 8.2 Hz, 2H), 7.79 (t, *J*=7.9 Hz, 1H), 7.63-7.44 (m, 4H), 7.32 (d, *J*=7.3 Hz, 2H), 2.72 (s, 3H). ^{13}C NMR (100 MHz, $CDCl_3$) δ 164.22, 146.88, 135.46, 131.87, 131.25, 130.02, 129.33, 129.18, 128.64, 128.55, 126.64, 123.31, 121.11, 118.89, 14.92. ESI-MS (m/z): $[M+H]^+$ 320.3.

Synthesis of 3. Compound **3'** (160 mg, 0.5mmol) was dissolved in DCM (20 mL), and *m*-CPBA (86 mg, 0.5 mmol) was added successively to the solution. The mixture was stirred for another 15 min at room temperature. Upon solvent evaporation, the crude product was purified by silica gel column chromatography (petroleum ether:AcOEt 3/1 to 0:1, 112 mg, 67%). ^1H NMR (300 MHz, $CDCl_3$) δ 8.80 (d, *J*=7.6 Hz, 1H), 8.72 (d, *J*=7.3 Hz, 1H), 8.47 (d, *J*=7.7 Hz, 1H), 8.34 (d, *J*=8.4 Hz, 1H), 7.94-7.82 (m, 1H), 7.65-7.42 (m, 3H), 7.32 (dd, *J*=5.1, 3.8 Hz, 2H), 2.92 (s, 3H). ^{13}C NMR (75 MHz, DMSO) δ 163.60, 163.40, 149.16, 134.84, 132.01, 131.22, 129.43, 128.90, 128.52, 128.43, 128.25, 127.48, 127.13, 125.04, 123.74, 123.07, 43.20. EI-MS (m/z, %): 335 (M^+, 70), 320 (71), 288 (100).

Synthesis of 4' Compound **4a** was prepared as described.[5] Compound **4a** (350 mg, 1mmol) was

dissolved in DMF (20 mL), and the solution of CH_3SNa (1.05 g, 0.2 mmol) was added slowly to the mixture. After the mixture was stirred overnight at room temperature and then poured into the water. The precipitate was filtered and washed with water twice to afford **4'** (272 mg, 86%). Due to poor solubility of the compound, the ^{13}C NMR spectra were not obtained. ^1H NMR (300 MHz, $CDCl_3$) δ 8.77 (dd, *J*=12.2, 7.3 Hz, 1H), 8.57 (d, *J*=8.1 Hz, 1H), 8.54-8.44 (m, 1H), 8.51 (d, *J*=7.9, 8.4 Hz, 1H), 7.92-7.81 (m, 1H), 7.75 (dt, *J*=11.1, 7.8 Hz, 1H), 7.52-7.38 (m, 3H), 2.67 (s, 3H). ESI-MS (m/z): $[M+H]^+$ 317.2.

Synthesis of 4. Compound **4'** (160 mg, 0.5mmol) was dissolved in DCM (20 mL), and *m*-CPBA (86 mg, 0.5 mmol) was added successively to the solution. The mixture was stirred for another 15 min at room temperature. Upon solvent evaporation, the crude product was purified by silica gel column chromatography (petroleum ether:AcOEt 3:1 to 0:1, 129 mg, 78% yield). Due to poor solubility of the compound, the ^{13}C NMR spectra were not obtained. ^1H NMR (300 MHz, $CDCl_3$) δ 8.83 (ddd, *J*=18.6, 13.2, 7.6 Hz, 2H), 8.55-8.39 (m, 2H), 8.22 (dd, *J*=8.5, 8.4 Hz, 1H), 7.84 (td, *J*=11.8, 8.2 Hz, 2H), 7.58-7.38 (m, 2H), 2.93 (s, 3H). EI-MS (m/z, %): 332 (M^+, 37), 317 (100), 44 (48).

Synthesis of 5'. Compound **5a** (152 mg, 1mmol) and 2,4-dimethylpyrrole (190 mg,2 mmol) were dissolved in dry DCM (75 mL) under argon. One drop of trifluoroacetic acid (TFA) was added to the solution and stirred for another 4 h at room temperature. 2,3-Dichloro-5,6-dicyanoquinone (DDQ, 221 mg, 1 mmol) was added, and the mixture was stirred for an additional 30 min. The reaction mixture was then treated with triethylamine (1.5 mL) for 5 min. Boron trifluoride etherate (1.5 mL) was added and the mixture was stirred for another 40 min, the dark brown solution was washed with water and brine (30 mL), dried over anhydrous magnesium sulfate, and concentrated at reduced pressure. The crude product was purified by silica-gel column chromatography (10% EtOAc-petroleum ether, 23% yield). ^1H NMR (400 MHz, $CDCl_3$) δ 7.35 (d, *J*=8.3 Hz, 2H), 7.19 (d, *J*=8.3 Hz, 2H), 5.99 (s, 2H), 2.56 (d, *J* = 2.8 Hz, 9H), 1.44 (s, 6H). ^{13}C NMR (100 MHz, $CDCl_3$) δ 155.44, 143.05, 141.17, 139.99, 131.49, 131.22, 128.36, 126.31, 121.20, 15.28, 14.64, 14.57. EI-MS (m/z, %): 370 (M^+, 36), 44(100).

Synthesis of 5. Compound **5'** (120 mg, 0.32 mmol) was dissolved in 2 DCM (20 mL), and *m*-CPBA (56 mg, 0.32 mmol) was added successively to the solution. The mixture was stirred for another 15 min at room temperature. Upon solvent evaporation, the crude product was purified by silica gel

column chromatography (petroleum ether:AcOEt 3/1 to 1:2, 70% yield). ^{1}H NMR (400 MHz, $CDCl_3$) δ 7.82 (d, *J*=8.3 Hz, 2H), 7.50 (d, *J*=8.3 Hz, 2H), 6.01 (s, 2H), 2.82 (s, 3H), 2.56 (s, 6H), 1.35 (s, 6H). ^{13}C NMR (100 MHz, $CDCl_3$) δ 156.15, 146.91, 142.68, 139.51, 138.12, 133.45, 130.11, 129.74, 129.30, 128.15, 124.36, 121.61, 44.09, 14.60, 14.48. EI-MS (m/z, %): 386 (M^+, 54), 371 (100), 261(55).

Synthesis of 6'. Compound **6a** (152 mg, 1mmol) and 2,4-dimethylpyrrole (190 mg,2 mmol) were dissolved in dry DCM (75 mL) under argon. One drop of TFA was added to the solution and stirred for another 4 h at room temperature. 2,3-Dichloro-5,6-dicyanoquinone (DDQ, 221 mg, 1 mmol) was added, and the mixture was stirred for an additional 30 min. The reaction mixture was then treated with triethylamine (1.5 mL) for 5 min. Boron trifluoride etherate (1.5 mL) was added and the mixture was stirred for another 40 min, the dark brown solution was washed with water and brine (30 mL), dried over anhydrous magnesium sulfate, and concentrated at reduced pressure. The crude product was purified by silica-gel flash column chromatography (10% EtOAc–petroleum ether, 17%). ^{1}H NMR (400 MHz, $CDCl_3$) δ 7.51-7.39 (m, 1H), 7.35-7.21 (m, 2H), 7.16 (dd, *J*=7.5, 1.2 Hz, 1H), 5.99 (s, 2H), 2.57 (s, 6H), 2.44 (s, 3H), 1.44 (s, 6H). ^{13}C NMR (100 MHz, $CDCl_3$) δ 155.69, 142.59, 139.22, 138.00, 132.87, 131.00, 129.70, 128.46, 125.41, 124.80, 121.06, 14.97, 14.66, 13.76. EI-MS (m/z, %): 370 (M^+, 34), 302 (190), 44(60).

Synthesis of 6. Compound **6'** (120 mg, 0.32 mmol) was dissolved in DCM (20 mL), and *m*-CPBA (56 mg, 0.32 mmol) was added successively to the solution. The mixture was stirred for another 15 min at room temperature. Upon solvent evaporation, the crude product was purified by silica gel column chromatography (petroleum ether:AcOEt 3:1 to 1:2, 63% yield). ^{1}H NMR (400 MHz, $CDCl_3$) δ 8.25 (d, *J*=7.8 Hz, 1H), 7.80 (dd, *J*=11.2, 4.2 Hz, 1H), 7.66 (t, *J*=7.5 Hz, 1H), , 6.05 (s, 1H), 6.00 (s, 1H), 2.61 (s, 3H), 2.58 (d, *J*=7.6 Hz, 6H), 1.44 (s, 3H), 1.34 (s, 3H). ^{13}C NMR (100 MHz, $CDCl_3$) δ 155.96, 144.22, 135.54, 134.58, 133.52, 132.04, 131.76, 131.12, 130.15, 129.76, 129.00, 128.20, 124.87, 122.33, 121.72, 42.12, 14.80, 14.62, 14.36, 14.24. EI-MS (m/z, %): 386 (M^+, 14), 337 (66), 319 (100).

Synthesis of 7b. 2,3,3-Trimethylindolenine (6.1 g, 38.3 mmol) and ethyl iodide (10.74 g, 68.8 mmol) were dissolved in toluene (30 mL) and stirred at 100 °C for 20 h. After the reaction finished, the mixture was cooled to room temperature and filtered. The crude compound was obtained with 87%

yield as pink solid without any purification for next step. ^{1}H NMR (300 MHz, $CDCl_3$) δ 7.69 (dt, *J*=6.3, 3.8 Hz, 1H), 7.52 (qd, *J*=5.8, 3.4 Hz, 3H), 4.63 (q, *J*=7.5 Hz, 2H), 3.05 (s, 3H), 1.56-1.52 (m, 9H). ^{13}C NMR (75 MHz, $CDCl_3$) δ 195.07, 141.35, 140.19, 129.86, 129.25, 123.20, 115.08, 54.36, 45.08, 22.80, 16.72, 13.42. MS: [M^+]187.6.

Synthesis of 7'. Compound **7b** was prepared according to the published procedure.[6] Compound **7b** (315 mg, 1mmol) and compound **5a** (182 mg, 1.2 mmol) were dissolved in ethanol (5 mL), and a few drops of piperidine were added as catalyst. Then the mixture was stirred under refluxing for 10 h. After the mixture cooled to room temperature, the product was precipitated, and obtained by filtration (69%). ^{1}H NMR (300 MHz, $CDCl_3$) δ 8.26-8.18 (m, 3H), 7.82 (d, *J*=16.1 Hz, 1H), 7.60 (dd, *J*=12.6, 3.0 Hz, 4H), 7.32 (d, *J*=8.4 Hz, 2H), 5.02 (q, *J*=7.2 Hz, 2H), 2.52 (s, 3H), 1.85 (s, 6H), 1.61 (t, *J*=7.3 Hz, 3H). ^{13}C NMR (75 MHz, $CDCl_3$) δ 180.93, 154.84, 149.00, 143.26, 140.19, 132.04, 129.84, 129.64, 125.50, 122.76, 114.47, 110.83, 52.25, 44.14, 27.14, 14.66, 14.35. ESI-MS: [M^+] 322.3.

Synthesis of 7. Compound **7'** (100 mg, 0.22 mmol) was dissolved in DCM (20 mL), and *m*-CPBA (38 mg, 0.22 mmol) was added successively to the solution. The mixture was stirred for another 15 min at room temperature. Upon solvent evaporation, the crude product was purified by silica gel column chromatography (DCM, 78% yield). ^{1}H NMR (300 MHz, $CDCl_3$) δ 8.53 (d, *J*=8.4 Hz, 2H), 8.33 (d, *J*=16.3 Hz, 1H), 8.04 (d, *J*=16.3 Hz, 1H), 7.81-7.68 (m, 3H), 7.62 (d, *J*=2.8 Hz, 3H), 5.23-4.98 (m, 2H), 2.76 (s, 3H), 1.90 (s, 6H), 1.65 (t, *J*=7.3 Hz, 3H). ^{13}C NMR (75 MHz, $CDCl_3$) δ 181.61, 153.01, 151.36, 143.72, 140.06, 135.83, 132.19, 130.49, 129.86, 124.35, 122.90, 115.19, 114.58, 52.88, 44.86, 43.73, 26.84, 26.79, 14.71. ESI-MS: [M^+] 338.3

Synthesis of 8'. Compound **7b** (315 mg, 1mmol) and Compound **6a** (182 mg,1.2 mmol) were dissolved in ethanol (5 mL), and a few drops of piperidine was added as catalyst. Then the mixture was stirred under refluxing for 10 h. After the mixture cooled to room temperature, the product was precipitated and was obtained by filtration with a yield of 56%. ^{1}H NMR (300 MHz, $CDCl_3$) δ 8.78 (d, *J*=16.1 Hz, 1H), 8.70 (d, *J*=7.9 Hz, 1H), 7.87 (d, *J*=16.1 Hz, 1H), 7.71 (dd, *J*=6.6, 3.1 Hz, 1H), 7.67-7.57 (m, 3H), 7.55-7.48 (m, 1H), 7.41 (dd, *J*=15.9, 7.8 Hz, 2H), 5.11 (q, *J*=7.3 Hz, 2H), 2.59 (s, 3H), 1.87 (s, 6H), 1.64 (t, *J*=7.3 Hz, 3H). ^{13}C NMR (75 MHz, $CDCl_3$) δ 181.74, 150.71, 143.39, 142.45, 140.27, 134.05, 132.25, 130.71, 130.04, 129.80, 127.31, 126.85, 122.82, 115.00, 113.42,

52.52, 44.84, 27.21, 16.88, 14.42. ESI-MS: [M^+] 322.2.

Synthesis of 8. Compound **8'** (100 mg, 0.22 mmol) was dissolved in DCM (20 mL), and *m*-CPBA (38 mg, 0.22 mmol) was added successively to the solution. The mixture was stirred for another 15 min at room temperature. Upon evaporation of the solvent, the crude product was purified by silica gel column chromatography (DCM, 76% yield). ^{1}H NMR (300 MHz, $CDCl_3$) δ 9.28 (d, *J*=16.2 Hz, 1H), 8.88 (d, *J*=7.3 Hz, 1H), 8.04 (d, *J*=16.2 Hz, 1H), 7.84-7.54 (m, 7H), 5.15 (q, *J*=7.3 Hz, 2H), 2.87 (s, 3H), 1.88 (d, *J*=3.8 Hz, 6H), 1.66 (t, *J*=7.5 Hz, 3H). ^{13}C NMR (75 MHz, $CDCl_3$) δ 181.96, 146.73, 144.98, 143.73, 140.22, 133.52, 132.46, 132.42, 131.64, 130.50, 129.86, 125.11, 122.85, 116.13, 115.31, 77.42, 77.00, 76.58, 52.87, 45.23, 44.32, 26.64, 26.51, 14.68. HRMS (m/z): [M^+] calcd. for 338.1573, found 338.1567.

Synthesis of 9. Compounds **9a** and **9'** were synthesized according to previous methods.[7] Compound **9'** (167 mg, 0.5 mmol) was dissolved in DCM (20 mL), and *m*-CPBA (86 mg, 0.5 mmol) was added successively to the solution. The mixture was stirred for another 15 min at room temperature. Upon solvent evaporation, the crude product was purified by silica gel column chromatography (AcOEt, 67% yield). ^{1}H NMR (300 MHz, $CDCl_3$) δ 8.11 (d, *J*=8.4 Hz, 2H), 7.96 (d, *J*=16.6 Hz, 1H), 7.80 (d, *J*=8.3 Hz, 2H), 7.34 (d, *J*=16.6 Hz, 1H), 2.80 (s, 3H), 1.81 (s, 6H). ^{13}C NMR (75 MHz, DMSO) δ 176.96, 174.64, 150.09, 145.59, 136.39, 129.82, 128.77, 124.32, 116.92, 112.53, 111.69, 110.66, 100.55, 99.55, 54.93, 43.00, 24.98. ESI-MS: $[M+H]^+$ 351.3.

Synthesis of 10'. To the mixture of compound **9a** (199 mg, 1mmol) and compound **6a** (182 mg,1.2 mmol) in ethanol (5 mL), a few drops of piperidine were added as catalyst. Then the mixture was stirred under refluxing for 12 h. After cooling the mixture to room temperature, the product was precipitated and obtained by filtration with a yield of 51%. ^{1}H NMR (300 MHz, $CDCl_3$) δ 8.12 (d, *J*=16.4 Hz, 1H), 7.71 (d, *J*=7.8 Hz, 1H), 7.53-7.42 (m, 1H), 7.38 (d, *J*=6.9 Hz, 1H), 7.30 (d, *J*=7.2 Hz, 1H), 7.07 (d, *J*=16.4 Hz, 1H), 2.55 (s, 3H), 1.86 (s, 6H). ^{13}C NMR (75 MHz, $CDCl_3$) δ 174.21, 143.66, 141.56, 132.57, 127.53, 127.19, 126.13, 116.10, 111.54, 110.78, 109.83, 100.95, 97.86, 26.77, 16.61. EI-MS (m/z, %): 333 (M^+, 100), 318 (72).

Synthesis of 10. Compound **10'** (167 mg, 0.5 mmol) was dissolved in DCM (20 mL), and *m*-CPBA (86 mg, 0.5 mmol) was added successively to the solution. The mixture was stirred for another 15 min at room temperature. Upon solvent evaporation, the crude product was purified by silica gel

column chromatography (AcOEt, 72% yield). ^{1}H NMR (300 MHz, $CDCl_3$) δ 8.46 (d, *J*=16.4 Hz, 1H), 7.90 (dd, *J*=9.9, 4.0 Hz, 2H), 7.78-7.58 (m, 2H), 7.11 (d, *J*=16.4 Hz, 1H), 2.78 (s, 3H), 1.85 (d, *J*=9.9 Hz, 6H). ^{13}C NMR (75 MHz, $CDCl_3$) δ 174.77, 173.55, 145.28, 139.73, 132.66, 132.24, 131.81, 127.54, 125.14, 117.76, 111.23, 110.60, 109.81, 102.01, 98.25, 58.57, 43.40, 26.21. EI-MS (m/z, %): 349 (M^+, 23), 332 (100).

Synthesis of 11'. Compounds **11a-d** were prepared as described.[8] To the mixture of compounds **11d** (208 mg, 1mmol) and **5a** (182 mg, 1.2 mmol) in toluene (5 mL), a few drops of piperidine and AcOH were added. Then the mixture was stirred under refluxing for 3 h. After the mixture cooled to room temperature, the product was precipitated and obtained by filtration with a yield of 41%. ^{1}H NMR (300 MHz, $CDCl_3$) δ 8.92-8.85 (m, 1H), 7.79-7.66 (m, 1H), 7.61-7.40 (m, 5H), 7.27 (s, 1H), 7.24 (s, 1H), 6.82 (s, 1H), 6.74 (d, *J*=15.9 Hz, 1H), 2.53 (s, 3H). ^{13}C NMR (75 MHz, $CDCl_3$) δ 157.50, 152.71, 152.25, 142.61, 138.32, 134.59, 130.97, 128.22, 125.96, 125.91, 125.76, 118.53, 117.77, 117.50, 116.79, 115.73, 106.62, 62.47, 15.04. EI-MS (m/z, %): 342 (M^+, 22), 44 (100).

Synthesis of 11. Compound **11'** (103 mg, 0.3 mmol) was dissolved in DCM (20 mL), and *m*-CPBA (52 mg, 0.3 mmol) was added successively to the solution. The mixture was stirred for another 15 min at room temperature. Upon solvent evaporation, the crude product was purified by silica gel column chromatography (AcOEt, 63% yield). ^{1}H NMR (400 MHz, $CDCl_3$) δ 8.93 (dd, *J*=8.4, 1.2 Hz, 1H), 7.83-7.71 (m, 5H), 7.65 (d, *J*=16.0 Hz, 1H), 7.61-7.57 (m, 1H), 7.52-7.44 (m, 1H), 6.92 (t, *J*=8.0 Hz, 2H), 2.79 (s, 3H). ^{13}C NMR (75 MHz, $CDCl_3$) δ 156.52, 152.57, 152.19, 147.62, 137.27, 136.89, 134.85, 128.53, 126.13, 125.79, 124.33, 120.78, 118.60, 117.69, 117.19, 116.42, 115.38, 107.66, 63.80, 43.81. EI-MS (m/z, %): 358 (M^+, 4), 342 (19), 44 (100).

Synthesis of 12'. To the mixture of compounds **11d** (208 mg, 1mmol) and **6a** (182 mg,1.2 mmol) in toluene (5 mL), a few drops of piperidine and AcOH were added as catalyst. Then the mixture was stirred under refluxing for 3h. After the mixture cooled to room temperature, the product was precipitated and obtained by filtration with a yield of 39%. ^{1}H NMR (300 MHz, $CDCl_3$) δ 8.91 (d, *J*=8.3 Hz, 1H), 8.13 (d, *J*=15.9 Hz, 1H), 7.74 (t, *J*=7.5 Hz, 1H), 7.60 (d, *J*=8.1 Hz, 2H), 7.46 (t, *J*=7.7 Hz, 1H), 7.36 (s, 2H), 7.27-7.16 (m, 1H), 6.87 (s, 1H), 6.76 (d, *J*=15.8 Hz, 1H), 2.54 (s, 3H).

^{13}C NMR (75 MHz, $CDCl_3$) δ 179.91, 157.34, 152.81, 152.32, 139.50, 135.64, 134.64, 133.66, 130.54, 127.31, 126.74, 125.97, 125.83, 125.77, 120.24, 118.78, 117.80, 116.70, 115.61, 107.15,

77.20, 16.59. EI-MS (m/z, %): 342 (M^+, 79), 327 (94), 135 (100).

Synthesis of 12. Compound **12'** (103 mg, 0.3 mmol) was dissolved in DCM (20 mL), and *m*-CPBA (52 mg, 0.3 mmol) was added successively to the solution. The mixture was stirred for another 15 min at room temperature. Upon solvent evaporation, the crude product was purified by silica gel column chromatography (AcOEt, 63% yield). ^{1}H NMR (300 MHz, $CDCl_3$) δ 8.92 (dd, J = 8.4, 1.2 Hz, 1H), 8.11 (dd, J=7.8, 1.2 Hz, 1H), 7.91 (d, J=15.7 Hz, 1H), 7.82-7.56 (m, 5H), 7.54-7.44 (m, 1H), 6.92 (s, 1H), 6.86 (d, J=15.7 Hz, 1H), 2.74 (s, 3H). ^{13}C NMR (75 MHz, $CDCl_3$) δ 155.96, 152.48, 152.14, 145.02, 135.00, 132.18, 131.53, 131.31, 126.58, 126.28, 125.80, 124.02, 122.59, 118.81, 117.67, 116.27, 115.24, 108.21, 64.42, 43.28. EI-MS (m/z, %): 358 (M^+, 44), 341 (100), 151 (88).

Synthesis of 13. Compound **13** was prepared according to the published procedure.[9]

Synthesis of 14'. To the solution of compound **14a** (50 mg, 0.25 mmol) in Tris-HCl buffer (50 mM, pH=8.0) was added the solution of CH_3SNa (1.31 g, 20% aqueous solution, 0.2 mmol) slowly. The mixture was monitored by TLC and extracted with chloroform (3x20 mL). Upon organic phase evaporation, the crude product was purified by silica gel column chromatography (petroleum ether:AcOEt=3:1, v/v) with a yield of 63%. ^{1}H NMR (400 MHz, $CDCl_3$) δ 8.43 (d, J=8.0 Hz, 1H), 7.11 (d, J=8.0 Hz, 1H), 2.78 (s, 3H). ^{13}C NMR (100 MHz, $CDCl_3$) δ 148.92, 142.57, 142.31, 130.76, 119.51, 14.65. ESI-MS: $[M+H]^+$ 212.2.

Synthesis of 14. Compound **14'** (55 mg, 0.25 mmol) was dissolved in DCM (20 mL), and *m*-CPBA (43 mg, 0.25 mmol) was added successively to the solution. The mixture was stirred for another 1 h. Upon solvent evaporation, the crude product was purified by silica gel column chromatography (petroleum ether:AcOEt form 3:1 to 0:1 (v/v), 68% yield). ^{1}H NMR (400 MHz, $CDCl_3$) δ 8.66 (d, J=7.2 Hz, 1H), 8.21 (d, J=3.6 Hz, 1H), 3.16 (s, 3H). ^{13}C NMR (100 MHz, $CDCl_3$) δ 145.83, 144.57, 142.57,129.24, 127.49, 41.59. EI-MS (m/z, %): 227 (M^+, 38),156 (100), 139 (85).

Synthesis of 15'. Compound **15a** (655 mg, 3.75mmol) and $NaNO_2$ (262 mg, 3.8 mmol) were dissolved in THF/H_2O (1:1) and stirred on ice water. Then con. HCl (0.5 mL) was added slowly to the solution. The mixture stirred for additional 30 min, the solution of CH_3SNa (1.31 g, 20% aqueous solution, 0.2 mmol) was added slowly to the mixture. After 15 min, the mixture was extracted with chloroform (3×20 mL). Upon organic phase evaporation, the crude product was purified by silica gel column chromatography (petroleum ether:AcOEt 3:1, 21% yield). ^{1}H NMR (400 MHz, $CDCl_3$) δ

7.47 (d, *J*=8.3 Hz, 1H), 7.14 (dd, *J*=10.6, 2.2 Hz, 2H), 6.21 (s, 1H), 2.54 (s, 3H), 2.41 (s, 3H). ^{13}C NMR (100 MHz, $CDCl_3$) δ 160.68, 153.98, 152.23, 144.74, 124.43, 121.74, 116.69, 113.61, 112.59, 18.57, 14.94. ESI-MS: $[M+H]^+$ 207.1.

Synthesis of 15. Compound **15'** (56 mg, 0.27 mmol) was dissolved in DCM (20 mL) and stirred at -78℃, and *m*-CPBA (46 mg, 0.27 mmol) was added successively to the solution. The mixture was stirred for another 30 min. Upon solvent evaporation, the crude product was purified by silica gel column chromatography (AcOEt, 86% yield). ^{1}H NMR (400 MHz, $CDCl_3$) δ 7.78 (d, *J*=8.8 Hz, 1H), 7.58 (dq, *J*=3.4, 1.6 Hz, 2H), 6.38 (d, *J*=1.1 Hz, 1H), 2.48 (d, *J*=1.2 Hz, 3H). ^{13}C NMR (100 MHz, $CDCl_3$) δ 159.65, 153.66, 151.47, 150.11, 125.82, 121.96, 118.70, 116.54, 112.39, 43.93, 18.70. ESI-MS: $[M+H]^+$ 223.1. HRMS (m/z) $[M + H]^+$ calcd for 223.0384, found 223.0425.

Synthesis of 16'. Compound **16a** was synthesized according to the reported method.[10] Compound **16a** (311 mg, 1mmol) was dissolved in acetone (20 mL), and K_2CO_3 (690 mg, 5 mmol) was added to the mixture. Subsequently, CH_3I (200 μL) was added dropwisely to the stirred mixture. After the mixture reacted for 10 min at room temperature, organic phase was filtered and concentrated in vacuum. The desired product was obtained by silica gel column chromatography (petroleum ether:AcOEt 3/1, 75% yield). ^{1}H NMR (400 MHz, $CDCl_3$) δ 9.01 (s, 1H), 8.08 (d, *J*=8.1 Hz, 1H), 7.98 (d, *J*=7.9 Hz, 1H), 7.55 (ddd, *J*=15.4, 8.5, 3.9 Hz, 2H), 7.47-7.40 (m, 1H), 7.23-7.18 (m, 2H), 2.58 (s, 3H). ^{13}C NMR (100 MHz, $CDCl_3$) δ 160.08, 159.77, 154.36, 152.46, 147.63, 141.20, 136.68, 128.97, 126.42, 125.22, 122.75, 122.62, 121.72, 118.48, 115.63, 111.83, 14.87. ESI-MS: $[M+H]^+$ 326.3

Synthesis of 16. Compound **16'** (185 mg, 0.57 mmol) was dissolved in DCM (20 mL), and *m*-CPBA (98 mg, 0.57 mmol) was added successively to the solution. The mixture was stirred for another 15 min at room temperature. Upon solvent evaporation, the crude product was purified by silica gel column chromatography (petroleum ether:AcOEt 1/1 to 0:1, 167 mg, 86% yield). ^{1}H NMR (400 MHz, $CDCl_3$) δ 9.04 (s, 1H), 8.06 (d, *J*=8.1 Hz, 1H), 7.95 (d, *J*=7.9 Hz, 1H), 7.85 (d, *J*=8.1 Hz, 1H), 7.70 (s, 1H), 7.60 (d, *J*=8.0 Hz, 1H), 7.52 (t, *J*=7.7 Hz, 1H), 7.45-7.38 (m, 1H), 2.83 (s, 3H). ^{13}C NMR (100 MHz, $CDCl_3$) δ 158.95, 158.93, 153.63, 152.31, 151.41, 140.02, 136.84, 130.24, 126.62, 125.68, 123.03, 121.72, 121.69, 120.74, 119.67, 112.17, 43.78. HRMS (m/z) $[M+H]^+$ calcd for 342.0214, found 342.0250.

Synthesis of 17'. Compound **17a** was synthesized according to the reported work.[11] Compound **17a** (295 mg, 1mmol) was dissolved in DMF (20 mL), and the solution of CH_3SNa (1.05g, 0.2 mmol) was added slowly to the mixture. After the mixture reacted for 2 h at room temperature, it was poured into water and the precipitate was filtered and washed with water twice to afford the produce (62%). ^1H NMR (300 MHz, $CDCl_3$) δ 7.48 (dd, *J*=9.0, 2.3 Hz, 1H), 6.56 (dd, *J*=9.0, 2.2 Hz, 1H), 6.46 (d, *J*=2.2 Hz, 1H), 5.80 (d, *J*=2.1 Hz, 1H), 3.51-3.27 (m, 4H), 2.49 (d, *J*=2.1 Hz, 3H), 1.45-1.07 (m, 6H). ^{13}C NMR (75 MHz, $CDCl_3$) δ 160.64, 157.40, 154.44, 150.71, 124.60, 108.31, 107.07, 100.37, 97.25, 44.69, 13.60, 12.39. ESI-MS: $[M+H]^+$ 264.5

Synthesis of 17. Compound **17'** (130 mg, 0.5 mmol) was dissolved in DCM (20 mL), and *m*-CPBA (86 mg, 0.5 mmol) was added successively to the solution. The mixture was stirred at room temperature. Upon solvent evaporation until compound **17'** was consumed monitoring by TLC, the crude product was purified by silica gel column chromatography (petroleum ether:AcOEt 3/1, 81% yield). ^1H NMR (300 MHz, $CDCl_3$) δ 7.19 (dd, *J*=8.7, 1.5 Hz, 1H), 6.65 (d, *J*=1.8 Hz, 1H), 6.61-6.50 (m, 2H), 3.58-3.27 (m, 4H), 2.88 (d, *J*=1.7 Hz, 3H), 1.50-1.01 (m, 6H). ^{13}C NMR (75 MHz, $CDCl_3$) δ 161.05, 160.28, 156.01, 151.13, 123.26, 108.87, 104.60, 103.15, 97.96, 44.83, 42.13, 12.33. ESI-MS: $[M+H]^+$ 280.5.

Synthesis of 18'. Compound **5a** (152 mg, 1 mmol) and malononitrile (66 mg, 1mmol) were dissolved in EtOH (20 mL) and stirred under argon, and one drop of piperidine was added as catalyst. After the mixture was stirred overnight at room temperature, the organic phase was removed in vacuum. The crude product refined on silica gel column chromatography (petroleum ether:AcOEt 3/1, 62% yield). ^1H NMR (300 MHz, $CDCl_3$) δ 7.82 (d, *J*=8.4 Hz, 2H), 7.67 (s, 1H), 7.32 (d, *J*=8.4 Hz, 2H), 2.56 (s, 3H). ^{13}C NMR (75 MHz, $CDCl_3$) δ 158.73, 149.37, 130.96, 126.99, 125.39, 114.15, 113.04, 80.10, 14.51. ESI-MS: $[M+H]^+$ 201.2.

Synthesis of 18. Compound **18'** (100 mg, 0.5 mmol) was dissolved in DCM (20 mL), and *m*-CPBA (86 mg, 0.5 mmol) was added successively to the solution. The mixture was stirred for another 15 min at room temperature. Upon solvent evaporation, the crude product was purified by silica gel column chromatography (petroleum ether:AcOEt 1/1 to 0:1, 167 mg, 87% yield). ^1H NMR (300 MHz, $CDCl_3$) δ 8.05 (d, *J*=8.3 Hz, 2H), 7.87 (s, 1H), 7.82 (s, 1H), 7.79 (s, 1H), 2.78 (s, 3H). ^{13}C NMR (75 MHz, $CDCl_3$) δ 158.32, 152.64, 132.81, 131.29, 131.08, 124.60, 113.07, 112.03, 85.00,

43.65. EI-MS (m/z, %): 216 (M^+, 72), 201 (100), 44 (26).

Synthesis of 19'. Compound **6a** (152 mg, 1 mmol) and malononitrile (66 mg, 1mmol) were dissolved in EtOH (20 mL) and stirred under argon, and one drop of piperidine was added as catalyst. After the mixture was stirred overnight at room temperature, the organic phase was removed in vacuum. The crude product refined on silica gel column chromatography (petroleum ether:AcOEt 3/1, 67% yield). ^{1}H NMR (300 MHz, $CDCl_3$) δ 8.35 (s, 1H), 8.02 (d, *J*=8.0 Hz, 1H), 7.55 (t, *J*=7.7 Hz, 1H), 7.43 (d, *J*=8.0 Hz, 1H), 7.36–7.29 (m, 1H), 2.55 (s, 3H). ^{13}C NMR (75 MHz, $CDCl_3$) δ 156.73, 142.37, 133.89, 129.74, 128.96, 127.71, 125.99, 113.59, 112.28, 84.42, 16.99. ESI-MS: $[M+H]^+$ 200.4.

Synthesis of 19. Compound **19'** (100 mg, 0.5 mmol) was dissolved in DCM (20 mL), and *m*-CPBA (98 mg, 0.5 mmol) was added successively to the solution. The mixture was stirred for another 15 min at room temperature. Upon solvent evaporation, the crude product was purified by silica gel column chromatography (petroleum ether:AcOEt 1/1 to 0:1, 167 mg, 83% yield). ^{1}H NMR (300 MHz, $CDCl_3$) δ 8.44 (s, 1H), 8.13 (d, *J*=7.9 Hz, 1H), 8.07 (d, *J*=7.9 Hz, 1H), 7.84 (t, *J*=7.4 Hz, 1H), 7.71 (t, *J*=7.3 Hz, 2H), 2.76 (s, 7H). ^{13}C NMR (75 MHz, $CDCl_3$) δ 153.87, 146.78, 134.43, 131.81, 129.13, 128.22, 124.97, 112.63, 111.68, 87.16, 43.92. EI-MS (m/z, %): 216 (M^+, 47), 146 (100).

Synthesis of 20'. Compound **20a** was synthesized according to the pervious procedure.[12] Compounds **5a** (182 mg, 1.2 mmol) and **20a** (174 mg, 1mmol) were dissolved in EtOH (20 mL) and stirred under argon, and one drop of piperidine was added as catalyst. After the mixture was stirred for overnight at room temperature, the product was precipitated and obtained by filtration with a yield of 80%. ^{1}H NMR (300 MHz, $CDCl_3$) δ 8.17 (d, *J*=2.0 Hz, 1H), 8.06 (dd, *J*=8.1, 0.6 Hz, 1H), 7.99-7.86 (m, 3H), 7.52 (ddt, *J*=8.3, 7.3, 1.2 Hz, 1H), 7.47-7.37 (m, 1H), 7.31 (dd, *J*=8.5, 1.5 Hz, 2H), 2.55 (s, 3H). ^{13}C NMR (75 MHz, $CDCl_3$) δ 163.02, 153.60, 146.12, 145.51, 134.89, 130.68, 128.53, 126.87, 125.83, 125.56, 123.42, 121.62, 116.80, 103.77, 14.73. ESI-MS: $[M+H]^+$ 308.6.

Synthesis of 20. Compound **20'** (154 mg, 0.5 mmol) was dissolved in DCM (20 mL), and *m*-CPBA (86 mg, 0.5mmol) was added successively to the solution. The mixture was stirred for another 15 min at room temperature. Upon solvent evaporation, the crude product was purified by silica gel column chromatography (petroleum ether:AcOEt 1/1 to 0:1, 167 mg, 92% yield). ^{1}H NMR (400 MHz, $CDCl_3$) δ 8.21 (s, 1H), 8.10 (d, *J*=8.3 Hz, 2H), 8.04 (d, *J*=8.1 Hz, 1H), 7.87 (d, *J*=7.9 Hz, 1H), 7.74 (d, *J*=8.4 Hz, 2H), 7.56-7.47 (m, 1H), 7.45-7.37 (m, 1H), 2.76 (s, 3H). ^{13}C NMR (100 MHz,

$CDCl_3$) δ 161.73, 153.33, 149.68, 144.76, 134.89, 134.55, 130.65, 126.96, 126.20, 124.17, 123.62, 121.59, 115.83, 107.44, 43.67. EI-MS (m/z, %): 324 (M^+, 52), 309 (100), 292 (46), 259 (49), 44 (40).

Synthesis of 21'. Compounds **6a** (182 mg, 1.2 mmol) and **20a** (174 mg, 1mmol) were dissolved in EtOH (20 mL) and stirred under argon, and one drop of piperidine was added as catalyst. After the mixture was stirred for overnight at room temperature, the product was precipitated and obtained by filtration with a yield of 78%. ^1H NMR (300 MHz, $CDCl_3$) δ 8.64 (s, 1H), 8.13 (d, *J*=8.1 Hz, 2H), 7.91 (d, *J*=8.0 Hz, 1H), 7.54 (t, *J*=7.4 Hz, 1H), 7.44 (dd, *J*=13.7, 7.4 Hz, 3H), 7.34 (t, *J*= .4 Hz, 1H), 2.55 (s, 3H). ^{13}C NMR (75 MHz, $CDCl_3$) δ 162.55, 153.60, 144.39, 141.05, 134.85, 131.82, 131.54, 128.93, 127.37, 126.83, 126.10, 125.85, 123.86, 121.55, 115.91, 108.13, 16.93. ESI-MS: $[M+H]^+$ 308.5.

Synthesis of 21. Compound **21'** (154 mg, 0.5 mmol) was dissolved in DCM (20 mL), and *m*-CPBA (86 mg, 0.5mmol) was added successively to the solution. The mixture was stirred for another 15 min at room temperature. Upon solvent evaporation, the crude product was purified by silica gel column chromatography (petroleum ether:AcOEt 1/1 to 0:1, 167 mg, 90% yield). ^1H NMR (300 MHz, $CDCl_3$) δ 8.60 (s, 1H), 8.22 (d, *J*=7.6 Hz, 1H), 8.14 (d, *J*=7.9 Hz, 2H), 7.94-7.91 (m, 1H), 7.77 (td, *J*=7.6, 1.3 Hz, 1H), 7.69 (td, *J*=7.6, 1.4 Hz, 1H), 7.58 (dd, *J*=7.2, 1.1 Hz, 1H), 7.51-7.44 (m, 1H), 2.76 (s, 3H). ^{13}C NMR (75 MHz, $CDCl_3$) δ 161.11, 153.46, 146.10, 140.08, 135.06, 132.54, 131.61, 129.85, 128.91, 127.16, 126.54, 124.29, 124.05, 121.71, 115.45, 110.28, 43.47. EI-MS (m/z, %): 324 (M^+, 5), 261 (100).

Synthesis of 22'.[13] To a mixture of **5a** (456 mg, 3 mmol) and **22a** (238 mg, 1 mmol) in DMF (20 mL), a drop of con. H_2SO_4 was added. The mixture was heated at 100 °C for 30 min with stirring. After the hot mixture was cooled to room temperature, it was poured into water. The yellow solid was collected by filtration and further purified by column chromatography to give the product (46%). ^1H NMR (400 MHz, $CDCl_3$) δ 11.21 (s, 1H), 8.37-8.30 (m, 1H), 8.29-8.23 (m, 1H), 8.20 (d, *J*=8.4 Hz, 1H), 8.05 (dd, *J*=12.6, 8.4 Hz, 3H), 7.85-7.75 (m, 2H), 7.37 (d, *J*=8.4 Hz, 2H), 2.57 (s, 3H). ^{13}C NMR (100 MHz, $CDCl_3$) δ 185.19, 156.37, 143.71, 134.42, 134.21, 133.73, 127.59, 127.24, 126.46, 126.01, 125.41, 124.53, 122.04, 117.86, 15.00. ESI-MS: $[M+H]^+$ 371.6.

Synthesis of 22. Compound **22'** (185 mg, 0.5 mmol) was dissolved in DCM (20 mL), and *m*-CPBA (86 mg, 0.5 mmol) was added successively to the solution. The mixture was stirred for another 15

min at room temperature. Upon solvent evaporation, the crude product was purified by silica gel column chromatography (AcOEt, 67% yield). ^{1}H NMR (300 MHz, $CDCl_3$) δ 11.42 (s, 1H), 8.42-8.25 (m, 5H), 8.17 (d, *J*=8.4 Hz, 1H), 7.96-7.78 (m, 4H), 2.83 (s, 3H). ^{13}C NMR (100 MHz, $CDCl_3$) δ 185.17, 182.55, 155.09, 149.28, 149.16, 134.58, 133.95, 133.89, 133.32, 133.15, 131.29, 129.13, 127.94, 127.68, 126.57, 126.09, 124.49, 122.23, 118.34, 99.99, 43.93. EI-MS (m/z, %): 386 (M^+, 8), 370 (70), 355 (33), 55 (100).

Synthesis of 23'.[13-14] A mixture of **5a** (456 mg, 3 mmol), 1,10-phenanthroline-5,6-dione (208 mg, 1 mmol) and ammonium acetate (1.54 g, 20 mmol) in glacial acetic acid (15 mL) was heated at 100 °C for 30 min with stirring. After the hot mixture was cooled to room temperature, it was poured into water. The yellow solid was collected by filtration and further purified by column chromatography (70%). ^{1}H NMR (300 MHz, $CDCl_3$) δ 8.77-8.61 (m, 2H), 8.42 (s, 2H), 7.95 (d, *J*=8.1 Hz, 2H), 7.67-7.49 (m, 4H), 7.11 (d, *J*=7.3 Hz, 2H), 2.38 (s, 3H). ^{13}C NMR (75 MHz, $CDCl_3$) δ 140.70, 128.41, 127.03, 126.40, 126.12, 125.35, 123.68, 121.68, 15.16. EI-MS (m/z, %): 340 (M^+, 48), 325 (19), 44 (100).

Synthesis of 23 Compound **23'** (170 mg, 0.5 mmol) was dissolved in DCM (20 mL), and *m*-CPBA (86 mg, 0.5 mmol) was added successively to the solution. The mixture was stirred for another 15 min at room temperature. Upon solvent evaporation, the crude product was purified by silica gel column chromatography (AcOEt, 72% yield). ^{1}H NMR (400 MHz, DMSO) δ 8.84 (d, *J*=8.3 Hz, 2H), 8.58 (d, *J*=7.7 Hz, 2H), 8.51 (d, *J*=8.3 Hz, 2H), 7.89 (d, *J*=8.3 Hz, 2H), 7.73 (t, *J*=7.4 Hz, 2H), 7.62 (t, *J*=7.3 Hz, 2H), 2.83 (s, 3H). ^{13}C NMR (100 MHz, DMSO) δ 148.68, 146.47, 133.14, 127.62, 127.02, 126.58, 125.15, 124.27, 123.87, 121.95, 43.09. EI-MS (m/z, %): 356 (M^+, 7), 340 (32), 325 (14), 44 (100).

HPLC Conditions.

Analysis of Msr A-mediated reduction of Msr-blue. HPLC analyses of the pure probe Msr-blue, pure compound **15'** and the reaction of Msr A-mediated Msr-blue reduction were performed on an Agilent 1100 series HPLC system. Msr-blue (10 μM) was incubated with DTT (5 mM) and Msr A (3 μg/mL) at 37 °C for 6 h in TE buffer. The reaction mixture was dried under reduced pressure, and the residue was extracted by ethyl acetate. The combined solvent was removed under vacuum and

the residue was reconstituted in the small volume of methanol. Msr-blue and the pure compound **15'** were both prepared as a 10 μM solution and were used as standard samples. All samples were passed through a 0.22 μm filter, and 20 μL of each sample was loaded onto Agilent ZORBAX SB-C18, reversed-phase column (5 μm, 4.6×150 mm) on an Agilent 1100 series HPLC system. The column was eluted with methanol/water (1-10 min 35:65; 12-20 min 70:30; 22-27 min 35:65). The flow rate was set at 0.6 mL min^{-1}. A UV/vis detector was used to monitor the products at wavelength from 200-400 nm (compound **15'**: 333 nm; Msr-blue-278 nm; the reaction mixture: 1-12min, 278 nm & 12-27min, 333 nm).

Analysis of the purity of probes. The probes were dissolved in methanol to prepare a solution of 20 μM. Both samples were passed through a 0.22 μm filter, and 20 μL of each sample was loaded onto Agilent ZORBAX SB-C18, reversed-phase column (5 μm, 4.6×150 mm) on an Agilent 1100 series HPLC system. The column was eluted with methanol/water (35:65 for Msr-blue; and 80:20 for the probe **16**). The flow rate was set at 0.6 mL min^{-1}. A UV/vis detector was used to monitor the eluates at wavelength of 278 nm for Msr-blue, and 365 nm for **16**.

Reduction of MsrA-blue by kidney lysate. The protein extract from mouse kidney was prepared and the total protein concentration was adjusted to 29.2 mg/ml determined by the Bradford method. MsrA-blue (10 μM), DTT (5 mM) and 260 μl (29.2 mg/ml) lysate was dissolved in 3.5 ml TE buffer in centrifuge tube, and then incubation at 37 ℃. In certain time interval (1, 2, 4, 6, 8, 12h, 24h), 500 μl solution was sucked from the mixture and mixed with 1ml acetone depositing at -20℃. After 30 min, the supernatants were collected by centrifugation (1000 g for 5 min). Upon solvent evaporation, the residues were dissolved in 500μl methanol. All samples were loaded onto Agilent ZORBAX SB-C18, reversed-phase column (5 μm, 4.6×150 mm) on an Agilent 1100 series HPLC system.

Biological Assays

Live cell imaging. HL-60 cells ($4x10^5$) and 293T cells ($4x10^5$) were seeded in 6-well plates and allowed to grow overnight. Then Msr-blue (10 μM) was added to the plate and continued culture for 4 h. To inhibit the enzyme activity in cells, 0.2% (V/V) DMSO was added 30 minutes prior to the

addition of the probe. The cells were washed with PBS and visualized and photographed under a laser scanning confocal microscope (Olympus FV1000).

Measuring Msrs activity in mouse tissues. All animal experiments were carried out according to the Principles of Laboratory Animal Care (People's Republic of China) and the Guidelines of the Animal Investigation Committee, and approved by the Animal Care and Use Committee of Lanzhou University. Kunming Mice, purchased from the Laboratory Animal Center of Lanzhou University, were sacrificed by decapitation, and different organs, *i. e.*, heart, brain, kidney, liver and spleen were collected. These organs were washed with iced PBS and cut to small pieces. After addition of ice-cold RIPA buffer (50 mM Tris–HCl pH 7.5, 2 mM EDTA, 0.5% deoxycholate, 150 mM NaCl, 1% TritonX-100, 0.1% SDS, 1 mM Na 3 VO 4 and 1 mM PMSF), the samples were homogenized on ice with a glass tissue homogenizer. The protein extracts were prepared by collecting the supernatants after centrifuging the tissue homogenates at 14000 g for 10 minutes. The protein concentration was determined by the Bradford method, and all samples were adjusted to 15 mg/ml. Unless otherwise stated, the Msrs activity in samples was measured as the following description. Fifty microliter of samples were added to a cuvette containing Msr-blue (10 μM) and DTT (5 mM) in a final volume of 500 μL. The increase of fluorescence was monitored. The Msr A protein levels in different samples were determined by Western blots.

Immunoprecipitation. The protein extract from mouse kidney was prepared as described above, and the total protein concentration was adjusted to 15 mg/ml determined by the Bradford method. The samples were incubated with anti-Msr A antibody (Santa Cruz Biotechnology) for 8 h at room temperature and further incubated with Protein A/G beads (Santa Cruz Biotechnology) for 8 h at room temperature. The beads were pelleted by centrifugation (1000 g for 5 min), and the supernatants were collected and immediately used to measure Msrs activity with Msr-blue. The efficiency of immunoprecipitation was determined by Western blots analysis.

Imaging Msrs activity in the cellular model of PD. PC12 cells ($2x10^5$) were seeded in 12-well plates and cultured in DMEM supplemented with 10% FBS, 2 mM glutamine, penicillin (100 units/mL), streptomycin (100 units/mL) at 37 °C in a humidified atmosphere of 5% CO_2 overnight. The cells were treated with 6-OHDA for 8 h. Then Msr-blue (10 μM) was added and continued incubation for another 4 h. The cells were washed with PBS and visualized and photographed under a laser

scanning confocal microscope (Olympus FV1000).

Determining Msrs activity and expression in the cellular model of PD. PC12 cells ($8x10^5$) were seeded in 60 mm-plates and cultured in DMEM supplemented with 10% FBS, 2 mM glutamine, penicillin (100 units/mL), streptomycin (100 units/mL) at 37 °C in a humidified atmosphere of 5% CO_2 overnight. After the cells were treated with 6-OHDA for 12 h, the cells were harvested and lysed with RIPA buffer. The protein concentrations were adjusted to 12 mg/ml. Msr-blue (10 μM) was incubated with DTT (5 mM) and 80 μl of cell lysates at 37 ^{o}C in TE buffer in a final volume of 0.5 ml, and the folds of fluorescence increment (F/F_0) were determined (λ_{ex}=335 nm). The protein level of Msr A in the lysate was determined by Western blots.

References

[1] Y. Wu, G. Xie, Y. Xu, L. Ma, C. Tong, D. Fan, F. Du, H. Yu, *J Transl Med* **2015**, *13*, 316.

[2] C. Zhang, Z. Liu, Y. Li, W. He, X. Gao, Z. Guo, *Chem Commun (Camb)* **2013**, *49*, 11430-11432.

[3] J. F. Zhang, Y. Zhou, J. Yoon, Y. Kim, S. J. Kim, J. S. Kim, *Org Lett* **2010**, *12*, 3852-3855.

[4] L. W. Song, Y. H. Yang, Q. Zhang, H. Tian, W. H. Zhu, *J Phys Chem B* **2011**, *115*, 14648-14658.

[5] W. Jiang, J. Tang, Q. Qi, Y. M. Sun, H. Y. Ye, D. W. Fu, *Dyes Pigments* **2009**, *80*, 279-286.

[6] Y. Li, Y. Sun, J. Li, Q. Su, W. Yuan, Y. Dai, C. Han, Q. Wang, W. Feng, F. Li, *J Am Chem Soc* **2015**, *137*, 6407-6416.

[7] aT. Chen, Y. Zheng, Z. C. Xu, M. Zhao, Y. N. Xu, J. N. Cui, *Tetrahedron Lett* **2013**, *54*, 2980-2982; bT. Zhang, K. P. Guo, L. Qiu, Y. Q. Shen, *Synthetic Commun* **2006**, *36*, 1367-1372.

[8] aY. Zheng, M. Zhao, Q. L. Qiao, H. Y. Liu, H. J. Lang, Z. C. Xu, *Dyes Pigments* **2013**, *98*, 367-371; bW. Sun, J. Fan, C. Hu, J. Cao, H. Zhang, X. Xiong, J. Wang, S. Cui, S. Sun, X. Peng, *Chem Commun (Camb)* **2013**, *49*, 3890-3892.

[9] R. S. Kathayat, N. S. Finney, *J Am Chem Soc* **2013**, *135*, 12612-12614.

[10] K. G. Reddie, W. H. Humphries, C. P. Bain, C. K. Payne, M. L. Kemp, N. Murthy, *Org Lett* **2012**, *14*, 680-683.

[11] W. H. Wu, S. M. Ji, W. T. Wu, J. Y. Shao, H. M. Guo, T. D. James, J. Z. Zhao, *Chem-Eur J* **2012**, *18*, 4953-4964.

[12] H. S. A. Elzahabi, *Eur J Med Chem* **2011**, *46*, 4025-4034.

[13] P. Das, A. K. Mandal, N. B. Chandar, M. Baidya, H. B. Bhatt, B. Ganguly, S. K. Ghosh, A. Das, *Chem-Eur J* **2012**, *18*, 15382-15393.

[14] W. Lin, L. Long, L. Yuan, Z. Cao, B. Chen, W. Tan, *Org Lett* **2008**, *10*, 5577-5580.
